# Association of 37 markers of ultra-processing with all-cause mortality: a prospective cohort study in the UK Biobank

**DOI:** 10.1016/j.eclinm.2025.103448

**Published:** 2025-08-26

**Authors:** Kathrin Marie Krost, Gerrit Eichner, Mathias Fasshauer, Nathalie Judith Eise

**Affiliations:** a Institute of Nutritional Science, Justus-Liebig University of Giessen, Goethestr. 55, Giessen 35390, Germany; b Mathematical Institute, Justus-Liebig University of Giessen, Arndtstr. 2, Giessen 35392, Germany; c Centre for Sustainable Food Systems, Justus-Liebig University of Giessen, Senckenbergstr. 3, Giessen 35390, Germany

**Keywords:** Food additives, Markers of ultra-processing, Mortality, NOVA classification, Ultra-processed food

## Abstract

**Background:**

In contrast to ultra-processed foods (UPFs), the association between specific markers of ultra-processing (MUPs) and all-cause mortality remains unexplored. As MUPs such as flavour, emulsifier, and sweetener may contribute differently to health outcomes, evaluating their individual associations with mortality is crucial.

**Methods:**

This population-based, prospective, exploratory cohort study included 186,744 UK Biobank participants aged 40 to 75, recruited between 2006 and 2010, each of whom completed at least one Oxford WebQ. To assess intake of UPFs and specific MUPs, up to ten matching commercial products were researched for each Oxford WebQ item and analysed concerning 57 previously described MUPs grouped into nine MUP categories. Of these, 37 specific MUPs were identified in one or more products. A food item was regarded as UPF if it contained at least one MUP. Cox proportional hazard regression models for all-cause mortality were applied, incorporating gram intake from UPFs, as well as from foods containing each of the nine MUP categories and 37 specific MUPs, in relation to gram total food intake (expressed as percentage of total food intake (%TFI)), using penalised cubic splines. The hazard ratio (HR)-nadir was defined as the lowest HR on the %TFI axis and rescaled to 1.

**Findings:**

Over a mean follow-up of 11.0 years, 10,203 deaths occurred. Five MUP categories were significantly (linear P < 0.05) related to all-cause mortality and the following HRs (confidence interval (CI)) were observed relative to the HR-nadir: flavour (40% versus 10 %TFI (HR-nadir); HR (CI) 1.20 (1.08 to 1.33); P < 0.0011), flavour enhancer (2% versus 0 %TFI; 1.07 (1.00 to 1.16); P = 0.025), colouring agent (20% versus 3 %TFI; 1.24 (1.10 to 1.39); P < 0.0001), sweetener (20% versus 0 %TFI; 1.14 (1.06 to 1.23); P < 0.0043), and varieties of sugar (10% versus 4 %TFI; 1.10 (1.03 to 1.16); P = 0.025). Furthermore, 13 specific MUPs were significantly associated with all-cause mortality (linear and/or non-linear P < 0.05), i.e., glutamate, ribonucleotide, acesulfame, saccharin, sucralose, caking agent, firming agent, gelling agent, thickener, fructose, inverted sugar, lactose, and maltodextrin. Among these, gelling agent was inversely associated with mortality risk. UPF intake was significantly (P < 0.05) associated with all-cause mortality. Major findings remained robust in sensitivity analyses.

**Interpretation:**

Total MUP-based definition UPF, five MUP categories, and several specific MUPs within these categories were significantly associated with all-cause mortality. Thus, interventions targeting UPF may be most effective when focused on these candidates, which should also be prioritised for future mechanistic studies.

**Funding:**

10.13039/501100001659Deutsche Forschungsgemeinschaft (DFG, 10.13039/501100001659German Research Foundation) (Project: SFB 1052/2 C6 to MF).

**Research in context**

**Evidence before this study**

Ultra-processed food (UPF) consumption has been linked to adverse health outcomes and UPF sales per capita in kg increased in the period from 2006 to 2019 throughout the world. Within the NOVA classification system, UPF corresponds to group 4 and can be detected by pre-defined markers of ultra-processing (MUPs). No studies have yet investigated the association of these specific MUPs with all-cause mortality.

**Added value of this study**

Consumption of the MUP categories flavour, flavour enhancer, colouring agent, sweetener, and varieties of sugar were significantly related to all-cause mortality whereas no significant association was found for processing aid, modified oil, protein source, and fibre in a cohort of 186,744 UK Biobank participants. The following 13 specific MUPs were significantly associated with all-cause mortality: glutamate, ribonucleotide, acesulfame, saccharin, sucralose, caking agent, firming agent, gelling agent, thickener, fructose, inverted sugar, lactose, and maltodextrin. Among these, gelling agent was inversely related to mortality risk. UPF intake was significantly associated with all-cause mortality. Major findings remained robust in sensitivity analyses.

**Implications of all the available evidence**

The results indicate that only some MUPs are linked to all-cause mortality whereas others have no association with this endpoint. The findings provide an initial framework for identifying MUPs that should be prioritised in interventions targeting UPFs and for future mechanistic studies. Additional studies should replicate the observed associations between MUPs and mortality using the methodology applied in this study and explore links with other health outcomes, as well as analyse the underlying mechanisms.

## Introduction

Ultra-processed foods (UPFs) are ready-to-eat food items that are usually high in fat, salt, and sugar, as well as low in dietary fibre, protein, and micronutrients.^1^ These extensively processed products are designed to appeal to as many consumers as possible through attractive packaging and intensive marketing, offering convenience, taste, and profitability while often replacing more traditional food sources.^1^^,^^2^

A strong link between UPF consumption and adverse health outcomes has been described in a recent systematic umbrella review.^3^ Moreover, UPF consumption increased food intake and body weight in two randomised controlled cross-over trials.^4^^,^^5^ UPF sales per capita in kg increased in the period from 2006 to 2019 throughout the world.^6^ A recent systematic review found a wide range of UPF consumption with the United States of America and the United Kingdom (UK) exceeding 50 percentage of total energy intake (%E), and Italy at around 10 %E.^7^ Thus, limiting highly-processed food has been recommended in several nutrition guidelines, e.g., from Brazil,^8^ France,^9^ and Japan.^10^

There are several classification systems for categorising food items according to their degree of processing including the most widely used NOVA classification.^1^ In the NOVA classification, food items are assigned to four groups depending on the nature, scope, and purpose of their industrial processing.^1^^,^^2^ UPF corresponds to NOVA group 4.^1^^,^^2^ An important step forward is the use of pre-defined markers of ultra-processing (MUPs) to detect UPF as suggested by Monteiro et al. and Davidou et al.^2^^,^^11^ MUPs are food ingredients that include cosmetic additives, such as flavour, colouring agent, and sweetener, and non-culinary ingredients, such as fructose, modified oil, and isolated protein.^2^^,^^11^ If at least one MUP is found in a food item, it is considered UPF.^2^^,^^11^ This objective MUP-based method to identify UPF was recently used by Davidou and co-workers in a large food market analysis.^12^ Using a MUP-based approach, our group recently demonstrated that flavour, emulsifier, and colouring agent are the most frequent markers to detect UPF in the UK food market^13^ and that the percentage of UPF items was significantly higher in plant-based meat products as compared to their meat-based counterparts.^14^

In contrast to UPF,^3^ no studies have yet investigated the association between specific MUPs and all-cause mortality. This knowledge gap is particularly relevant, as certain MUPs, including flavour,^15^ colouring agent,^16^ sweetener,^17^ emulsifier,^18^ and gluten,^19^ may directly contribute to the adverse health effects of UPF, while other MUPs may have negligible or no adverse health effects. As these MUPs, therefore, may contribute differently to health outcomes, evaluating their individual associations with mortality is crucial. The present exploratory study is the first to systematically examine the association of gram intake from MUP-based definition UPF, as well as from foods containing each of the nine MUP categories and 37 specific MUPs, in relation to gram total food intake with all-cause mortality.

## Methods

### Study design, participants, and exclusion criteria

All analyses were based on the UK Biobank study, a prospective cohort study with more than 500,000 participants.^20^ Participants were recruited between 2006 and 2010 across the UK.^20^ For the current study, 210,839 participants were selected who completed at least one web-based 24-h dietary recall (Oxford WebQ) (Supplementary Fig. S1).^21^ The following exclusion criteria were applied resulting in 186,744 participants included in the primary analyses: (1) missing lifestyle factors, i.e., physical activity and smoking status, (2) implausible event/censoring data, (3) missing socio-economic factors, i.e., ethnic background, general health status, highest qualification, and Townsend deprivation index, (4) missing exam parameters, i.e., body mass index (BMI) and systolic blood pressure (SBP), (5) pre-existing malabsorption, (6) pre-existing diabetes mellitus, (7) implausible energy intake, i.e., total energy intake of 0 kcal or  <(1.1 × basal metabolic rate - 500 kcal) or >(2.5 × basal metabolic rate + 500 kcal) or belonging to the top 0.1 %E (Supplementary Fig. S1). Basal metabolic rate was defined according to the Oxford equation.^22^

### Exposure assessment

For assessing the intake of UPF and specific MUPs, ingredient lists, portion sizes in grams, and energy content in kJ per 100 g of up to ten matching commercial products from the two market leaders of groceries in the UK, i.e., Tesco and Sainsbury’s,^23^ were researched online for each Oxford WebQ item as described recently.^13^ Food products within the subcategories best matching the Oxford WebQ questions were sorted by relevance (Tesco) or by top sellers (Sainsbury’s). The threshold of ten commercial products was chosen to ensure sufficient representativeness while maintaining feasibility across the entire set of Oxford WebQ items. Using this approach, identification of ten relevant commercial products was feasible for the majority of items. In case of fewer than ten matching products found at Tesco and Sainsbury’s combined, all available products were included in the analysis. The Oxford WebQ provides information on the consumption of 206 food and 32 beverage items on the previous day, with some items including follow-up questions that are part of the respective item but require additional commercial products to accurately assess the intake of UPFs and MUPs. For instance, the assessment of coffee and tea intake is followed by questions regarding the addition of milk, sweetener, and sugar, which are not counted as separate items. In total, 2459 different products were analysed for the present study.

Definition of the 57 MUPs was based on publications by Monteiro and co-workers.^1^^,^^2^ The MUPs were grouped into nine categories, i.e., flavour, flavour enhancer, colouring agent, sweetener, processing aid, varieties of sugar, modified oil, protein source, and fibre (Supplementary Table S1). To maximise the identification of these MUPs within ingredient lists, the search terms were optimised as described in Supplementary Table S1. For example, for capturing the specific MUP glutamate, the ingredient lists were searched for the individual compounds E620 to E625, glutamate, glutamic acids, and MSG (Supplementary Table S1). All ingredient lists were analysed concerning the presence of these 57 MUPs and a food item was regarded as UPF if it contained at least one MUP. A total of 37 out of 57 MUPs were identified in the ingredient lists of at least one commercial product (Supplementary Table S1). For each Oxford WebQ item and each MUP, a Marker Likelihood Index (MLI) was calculated, based on the proportion of the commercial products containing this specific MUP. For example, for field 102260 of the Oxford WebQ (“chocolate bars”), six out of the ten ingredient lists contained flavour; thus, the MLI for flavour was 0.6 (Supplementary Table S2).

For each Oxford WebQ item, energy content was calculated as the mean energy content of the up to ten commercial products. The Oxford WebQ specifies portion size for each item. If portion sizes were reported in grams or millilitres, the reported values were used, converting millilitres into grams based on specific gravities. In case of other portion sizes given in the Oxford WebQ, such as “Mug/Cup”, the packaging information of the food item was used, and if packaging information was missing, the Food Standard Agency’s standard portion sizes were applied.^24^ The mean portion size for each Oxford WebQ item was then calculated based on the collected portion sizes of the up to ten commercial products. Mean portion sizes of each Oxford WebQ item were then multiplied with the MLI. For instance, since the portion size of the chocolate bars provided by the Oxford WebQ was 50 g, 30 g (50 g portion size ∗ 0.6 MLI for flavour) were regarded as flavoured. For each participant, the proportion of food intake containing a specific MUP relative to total food intake (%Total Food Intake = %TFI) was calculated by dividing the cumulative portion sizes of foods containing this MUP (in grams) by the total food intake (in grams). For example, if a participant consumed 1000 g of flavoured food and had a total food intake of 2000 g, the proportion of food containing flavour was 50 %TFI. The same method was applied to all other MUPs, as well as to calculate %TFI for UPF.

Each participant completed the Oxford WebQ up to five times with baseline assessments at the assessment centres conducted between April 2009 and September 2010 and follow-up online assessments taking place between February 2011 and June 2012. Participant counts by location of assessment centres are depicted in Supplementary Fig. S2. In case of more than one completed Oxford WebQ, the mean dietary intake was used for all primary and sensitivity analyses except for the sensitivity analysis in which only the first completed Oxford WebQ was evaluated.

### Outcome assessment

Mortality data, including dates of death, were supplied by the National Health Service (NHS) Information Centre for participants from England and Wales, and by the NHS Central Register, Scotland for participants from Scotland.^25^ The follow-up period was determined as the time from the last dietary assessment to either the date of death or of censoring induced by loss-to-follow-up or by the cut-off date of data acquisition (December 19, 2022), whichever event occurred first.

### Statistical analysis

All data analyses and graphical representations were performed using R (version: 4.4.2)^26^ together with the add-on packages readxl (1.4.3),^27^ tidyverse (2.0.0),^28^ survival (3.8.3),^29^ maps (3.4.3),^30^ VIM (6.2.2),^31^ and missRanger (2.6.1).^32^ Hazard ratio (HR) for mortality was calculated using Cox proportional hazard regression models. Intake of UPF or specific MUPs (%TFI), as well as total energy intake, were included as penalised cubic splines using eight base functions resulting in twelve knots. The placement of knots was determined automatically by the pspline function of the survival package, based on the range of the exposure variable. Models were adjusted for age (split by quintiles), alcohol intake (derived from the Oxford WebQ; <1, 1 to <8, 8 to <16, ≥16 g/day), BMI (<18.5, 18.5 to <25, 25 to <30, ≥30 kg/m^2^), ethnic background (White, group composed of Mixed, Asian, Black, Chinese, and other), general health status (poor, fair, good, excellent), highest qualification (none of the below, national exams at age 16 years, vocational qualifications or optional national exams at ages 17–18 years, professional, college or university), history of psychiatric disease (i.e., depression, anxiety/panic attacks, nervous breakdown, schizophrenia, deliberate self-harm/suicide attempt, mania/bipolar disorder/manic depression, alcohol dependency, opioid dependency, other substance abuse/dependency, post-traumatic stress disorder, anorexia/bulimia/other eating disorder, post-natal depression, stress, obsessive compulsive disorder, or insomnia), household income (<18, 18 to <31, 31 to <52, 52 to <100, ≥100 k£, unknown), physical activity (Metabolic Equivalent of Task (MET) per week derived from the Oxford WebQ; split by quintiles), SBP (split by quintiles), sex (female, male), smoking status (never, previous, current occasional, current <10, 10 to 14, 15 to 19, ≥20 cigarettes per day), and Townsend deprivation index (split by quintiles). Covariates were assessed for violations of the proportional hazard assumption using the cox.zph function from the survival package, which evaluates scaled Schoenfeld residuals. All covariates showing statistically significant violations after Holm adjustment for multiple testing were stratified in the final models using the strata function. In all analyses, the HR-nadir was defined as the lowest HR value on the %TFI axis between zero and the 99th percentile of the observed intakes. This HR-nadir was rescaled to 1 to simplify comparison of different levels of MUP intake. All HRs were given with their pointwise 95% confidence intervals (CI). Furthermore, analyses of penalised cubic spline terms were split into their linear and non-linear effects. P-values of <0.05 were regarded as statistically significant.

### Sensitivity analyses

The following sensitivity analyses were performed to evaluate the robustness of the results: (1) Exclusion of participants with a follow-up period of less than 2 years (landmark analysis) to address reverse causation. (2) Exclusion of participants who reported unintentional weight loss during the touchscreen questionnaire assessment at baseline compared with one year ago since this might indicate disease states including cancer, chronic organ failure, and frailty. (3) Exclusion of participants who reported at least once that their previous day’s diet was not typical to exclude non-representative dietary data. (4) Exclusion of participants with one completed Oxford WebQ to address potential lower reproducibility in UPF and MUP intake based on a single Oxford WebQ. (5) Exclusion of participants with a history of cardiovascular disease or cancer since they might be prone to premature death. (6) Calculation of UPF and MUP intake based on the first Oxford WebQ only since it was filled out most adjacent to the baseline assessment. (7) Further adjustment for diet quality score to assess whether overall diet quality might act as a confounder or a mediator. (8) Adjustment for waist-to-hip ratio and height instead of BMI to better control for fat distribution. (9) Removal of energy intake as covariate since it might be a mediator. (10, 11) Application of no exclusion criteria except implausible event/censoring data and using (10) k-nearest neighbours or (11) random forrest imputation methods to decrease the number of excluded participants and missing values in covariates. (12) Further adjustment for cholesterol lowering medication, blood pressure medication, and insulin therapy to assess the potential influence of cardiometabolic medication use. (13) Modelling the two continuous covariates BMI and SBP showing indications of non-linearity based on martingale residual diagnostics (data not shown) using penalised cubic splines to better capture their association with the log hazard. (14) Further adjustment for assessment centre to consider the potential influence of geographical factors. (15) Analysis performed using an MLI calculated based on the first eight, six, and four commercial products for assessing the robustness of the MLI for UPF and MUP categories showing significant associations in the main analysis. (16) Assessment of cumulative specific MUP intake as an additional marker for MUP exposure. Cumulative specific MUP intake was defined as the sum of all specific MUPs in %TFI, e.g., if a participant had a %TFI intake for flavour of 3%, acesulfame of 2%, emulsifier of 5%, and no consumption of other specific MUPs apart from these three, the cumulative specific MUP intake was 10. (17) Association of MUP categories and specific MUPs with all-cause mortality in the main and all sensitivity analyses further adjusted for multiple testing using the Benjamini-Hochberg method for the MUP categories and specific MUPs separately and considering results with a false discovery rate adjusted P-value < 0.05 as statistically significant.

### Ethics

The UK Biobank study received approval from the North West Multicentre Research Ethics Committee and all participants provided written informed consent at baseline.^20^

### Role of the funding source

This work was supported by a grant of the Deutsche Forschungsgemeinschaft (DFG, German Research Foundation) (SFB 1052/2 C6) to MF. The funder had no role in the design, analysis, or writing of this article, and the decision to submit.

## Results

### Baseline data of UK Biobank participants

The baseline characteristics of the overall study population and the subgroups based on quintiles of UPF intake are presented in Table 1, Supplementary Fig. S3 and Table S3. Baseline characteristics of the included and excluded participants are compared in Supplementary Table S4. The mean (standard deviation (SD)) age of the study cohort was 58 (8) years and UPF intake (%TFI) was 20.0 (8.9) (Table 1). Within the MUP categories, mean (SD) %TFI was highest for flavour (13.6 (8.3)) followed by processing aid (7.6 (3.5)), and colouring agent (5.6 (4.1)) (Table 1). Over a follow-up period of 11.0 (1.6) years and 2.0 million person-years, a total of 10,203 deaths occurred.

**Table 1:** Baseline characteristics of the UK Biobank cohort.^a^

Parameters	Total cohort (n = 186,744)	Quintiles of UPF intake (%TFI)				
		0.4 to 12.9 (n = 37,349)	12.9 to 16.5 (n = 37,349)	16.5 to 20.3 (n = 37,348)	20.3 to 26.0 (n = 37,349)	26.0 to 98.7 (n = 37,349)
Age (years)	58 (8)	58 (8)	59 (8)	59 (8)	59 (8)	57 (8)
Alcohol (g/day)						
<1	66,643 (35.7)	9077 (24.3)	10,863 (29.1)	12,459 (33.4)	14,805 (39.6)	19,439 (52.0)
1 to <8	19,958 (10.7)	2405 (6.4)	3747 (10.0)	4514 (12.1)	4891 (13.1)	4401 (11.8)
8 to <16	26,317 (14.1)	4247 (11.4)	5615 (15.0)	5806 (15.5)	5839 (15.6)	4810 (12.9)
≥16	73,826 (39.5)	21,620 (57.9)	17,124 (45.8)	14,569 (39.0)	11,814 (31.6)	8699 (23.3)
BMI (kg/m^2^)						
<18.5	1071 (0.6)	224 (0.6)	209 (0.6)	212 (0.6)	240 (0.6)	186 (0.5)
18.5 to <25	72,940 (39.1)	15,404 (41.2)	15,389 (41.2)	15,010 (40.2)	14,380 (38.5)	12,757 (34.2)
25 to <30	78,358 (42.0)	15,727 (42.1)	15,626 (41.8)	15,698 (42.0)	15,655 (41.9)	15,652 (41.9)
≥30	34,375 (18.4)	5994 (16.0)	6125 (16.4)	6428 (17.2)	7074 (18.9)	8754 (23.4)
Energy (kJ)	8915 (2248)	8355 (2152)	8798 (2116)	9016 (2166)	9200 (2253)	9203 (2426)
Ethnic background						
White	180,017 (96.4)	35,949 (96.3)	36,157 (96.8)	36,173 (96.9)	36,030 (96.5)	35,708 (95.6)
Group composed of Mixed, Asian, Black, Chinese, and other	6727 (3.6)	1400 (3.7)	1192 (3.2)	1175 (3.1)	1319 (3.5)	1641 (4.4)
General health status						
Poor	4536 (2.4)	754 (2.0)	757 (2.0)	764 (2.0)	921 (2.5)	1340 (3.6)
Fair	29,915 (16.0)	5287 (14.2)	5450 (14.6)	5649 (15.1)	6186 (16.6)	7343 (19.7)
Good	113,277 (60.7)	22,485 (60.2)	22,791 (61.0)	23,002 (61.6)	22,868 (61.2)	22,131 (59.3)
Excellent	39,016 (20.9)	8823 (23.6)	8351 (22.4)	7933 (21.2)	7374 (19.7)	6535 (17.5)
Highest qualification						
None of the below	14,993 (8.0)	2634 (7.1)	2816 (7.5)	2883 (7.7)	3132 (8.4)	3528 (9.4)
National exams at age 16 years	28,129 (15.1)	5384 (14.4)	5361 (14.4)	5372 (14.4)	5705 (15.3)	6307 (16.9)
Vocational qualifications or optional national exams at ages 17–18 years	33,080 (17.7)	6389 (17.1)	6249 (16.7)	6386 (17.1)	6689 (17.9)	7367 (19.7)
Professional	29,031 (15.6)	5527 (14.8)	5608 (15.0)	5887 (15.8)	6049 (16.2)	5960 (16.0)
College or university	81,511 (43.7)	17,415 (46.6)	17,315 (46.4)	16,820 (45.0)	15,774 (42.2)	14,187 (38.0)
History of psychiatric disease						
Yes	12,290 (6.6)	2176 (5.8)	2237 (6.0)	2319 (6.2)	2561 (6.9)	2997 (8.0)
No	174,454 (93.4)	35,173 (94.2)	35,112 (94.0)	35,029 (93.8)	34,788 (93.1)	34,352 (92.0)
Household income/year (k£)						
<18	25,012 (13.4)	4169 (11.2)	4542 (12.2)	4897 (13.1)	5363 (14.4)	6041 (16.2)
18 to <31	40,844 (21.9)	7248 (19.4)	7956 (21.3)	8320 (22.3)	8642 (23.1)	8678 (23.2)
31 to <52	48,463 (26.0)	9460 (25.3)	9656 (25.9)	9874 (26.4)	9733 (26.1)	9740 (26.1)
52 to <100	41,711 (22.3)	9388 (25.1)	8702 (23.3)	8364 (22.4)	7875 (21.1)	7382 (19.8)
≥100	12,407 (6.6)	3592 (9.6)	2840 (7.6)	2297 (6.2)	2014 (5.4)	1664 (4.5)
Unknown	18,307 (9.8)	3492 (9.3)	3653 (9.8)	3596 (9.6)	3722 (10.0)	3844 (10.3)
Physical activity (MET per week)	4133 (2653)	4193 (2727)	4188 (2606)	4146 (2583)	4106 (2600)	4034 (2743)
SBP (mmHg)	138.5 (19.4)	139.2 (19.8)	138.8 (19.4)	138.7 (19.3)	138.4 (19.3)	137.6 (19.2)
Sex						
Female	106,958 (57.3)	21,496 (57.6)	21,791 (58.3)	21,430 (57.4)	21,035 (56.3)	21,206 (56.8)
Male	79,786 (42.7)	15,853 (42.4)	15,558 (41.7)	15,918 (42.6)	16,314 (43.7)	16,143 (43.2)
Smoking status						
Never	107,346 (57.5)	19,556 (52.4)	21,233 (56.9)	21,830 (58.5)	22,255 (59.6)	22,472 (60.2)
Previous	65,959 (35.3)	14,548 (39.0)	13,479 (36.1)	13,084 (35.0)	12,687 (34.0)	12,161 (32.6)
Current occasional	4474 (2.4)	1127 (3.0)	929 (2.5)	832 (2.2)	801 (2.1)	785 (2.1)
<10 cigarettes per day	2306 (1.2)	558 (1.5)	437 (1.2)	443 (1.2)	413 (1.1)	455 (1.2)
10–14 cigarettes per day	2010 (1.1)	492 (1.3)	372 (1.0)	356 (1.0)	361 (1.0)	429 (1.1)
15–19 cigarettes per day	1793 (1.0)	397 (1.1)	371 (1.0)	315 (0.8)	318 (0.9)	392 (1.0)
≥20 cigarettes per day	2856 (1.5)	671 (1.8)	528 (1.4)	488 (1.3)	514 (1.4)	655 (1.8)
Townsend deprivation index	−1.7 (2.8)	−1.6 (2.9)	−1.7 (2.8)	−1.8 (2.8)	−1.7 (2.8)	−1.5 (2.9)
UPF intake (%TFI)	20.0 (8.9)	10.2 (2.0)	14.8 (1.0)	18.4 (1.1)	22.9 (1.6)	33.9 (7.9)
MUP category intake (%TFI)						
Flavour	13.6 (8.3)	5.7 (2.0)	8.9 (2.0)	11.8 (2.4)	15.7 (3.0)	26.1 (8.4)
Flavour enhancer	0.3 (0.5)	0.2 (0.4)	0.3 (0.4)	0.3 (0.5)	0.4 (0.6)	0.4 (0.7)
Colouring agent	5.6 (4.1)	2.4 (1.2)	3.7 (1.5)	4.9 (1.9)	6.5 (2.5)	10.6 (5.3)
Sweetener	4.6 (6.0)	0.8 (1.1)	1.8 (1.8)	3.0 (2.6)	5.0 (3.7)	12.2 (8.6)
Processing aid	7.6 (3.5)	4.6 (1.7)	6.4 (2.0)	7.6 (2.4)	8.8 (3.0)	10.6 (4.4)
Varieties of sugar	4.7 (2.5)	2.8 (1.2)	3.7 (1.4)	4.5 (1.7)	5.3 (2.1)	7.0 (3.2)
Modified oil	0.0 (0.1)	0.0 (0.0)	0.0 (0.1)	0.0 (0.1)	0.1 (0.1)	0.1 (0.1)
Protein source	2.7 (1.9)	1.6 (1.0)	2.3 (1.2)	2.7 (1.5)	3.2 (1.8)	3.8 (2.6)
Fibre	1.0 (0.9)	0.6 (0.5)	0.8 (0.7)	1.0 (0.8)	1.2 (1.0)	1.4 (1.3)

%TFI, Percentage total food intake; BMI, Body mass index; MET, Metabolic equivalent of task; MUP, Marker of ultra-processing; SBP, Systolic blood pressure; SD Standard deviation; UK, United Kingdom; UPF, Ultra-processed food.

a Categorical variables are summarised as frequencies (percentages) and continuous variables as mean (SD).

### Main analyses

#### Association of UPF and nine MUP categories with all-cause mortality

**Fig. 1:**
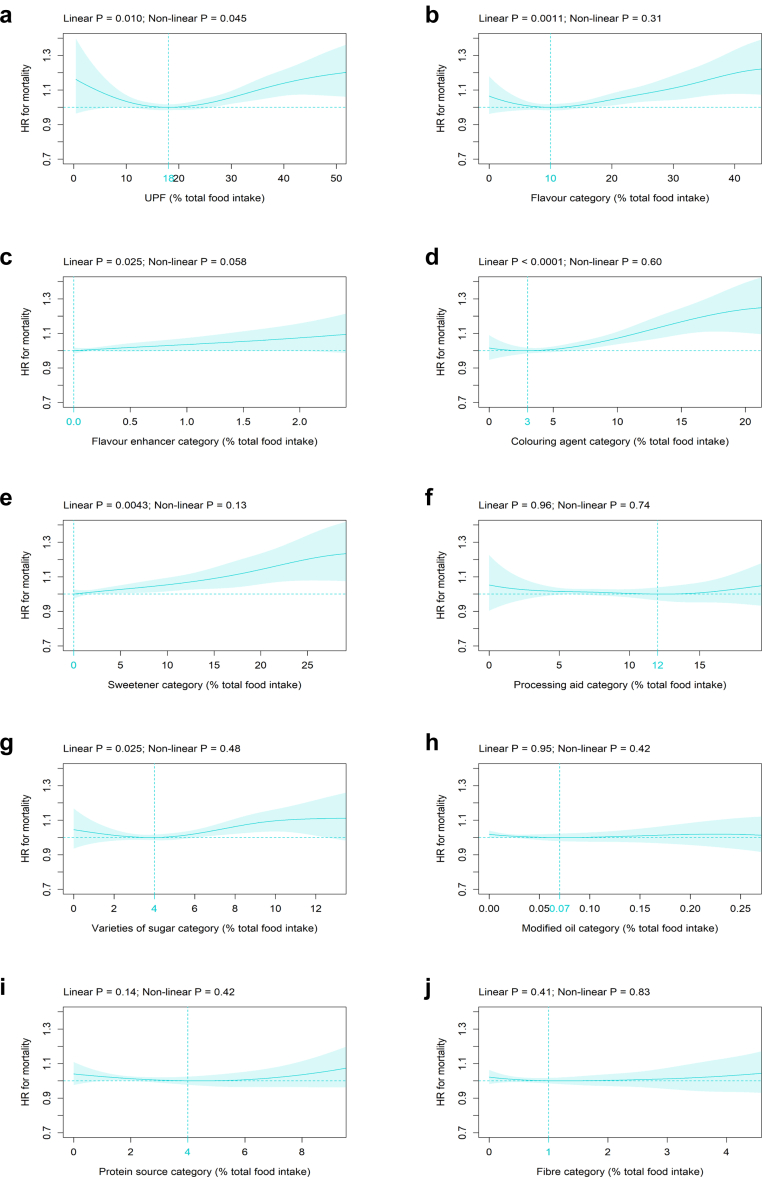
Association of MUP categories with all-cause mortality. Association of (all %TFI) (a) UPF, as well as the MUP categories (b) flavour, (c) flavour enhancer, (d) colouring agent, (e) sweetener, (f) processing aid, (g) varieties of sugar, (h) modified oil, (i) protein source, and (j) fibre, with all-cause mortality (n = 186,744; number of events = 10,203). Models are adjusted for age, alcohol intake, BMI, energy intake, ethnic background, general health status, highest qualification, history of psychiatric disease, household income, physical activity, SBP, sex, smoking status, and Townsend deprivation index as indicated in the Methods section. Abbreviations: %TFI, Percentage total food intake; BMI, Body mass index; HR, Hazard ratio; MUP, Marker of ultra-processing; SBP, Systolic blood pressure; UPF, Ultra-processed food.

UPF consumption was significantly associated with all-cause mortality with the HR-nadir observed at 18 %TFI and an increase in risk beyond that intake (Fig. 1a). In comparison to intake at the HR-nadir, mean (pointwise 95% CI) HR increased to 1.06 (1.02 to 1.09), 1.14 (1.07 to 1.22), and 1.19 (1.06 to 1.34) at 30, 40, and 50 %TFI, respectively (Fig. 1a). For the flavour category, a significant linear increase in all-cause mortality risk was seen beyond the HR-nadir at 10 %TFI with the HR increased to 1.04 (1.01 to 1.08), 1.11 (1.04 to 1.18), and 1.20 (1.08 to 1.33) at 20, 30, and 40 %TFI, respectively (Fig. 1b). A significant linear association with all-cause mortality and a HR-nadir at 0 %TFI was found for the flavour enhancer category (Fig. 1c). For the colouring agent category, a significant linear increase in all-cause mortality risk was found beyond the HR-nadir at 3 %TFI with the HR increased to 1.07 (1.04 to 1.11) and 1.24 (1.10 to 1.39) at 10 and 20 %TFI, respectively (Fig. 1d). A significant linear relation with all-cause mortality and a HR-nadir at 0 %TFI was found for the sweetener category and the HR increased to 1.05 (1.02 to 1.09) and 1.14 (1.06 to 1.23) at 10 and 20 %TFI, respectively (Fig. 1e). For the varieties of sugar category,a significant linear increase in all-cause mortality risk was found beyond the HR-nadir at 4 %TFI with the HR increased to 1.10 (1.03 to 1.16) at 10 %TFI (Fig. 1g).

In contrast to these MUP categories, no significant association with all-cause mortality was seen for the following categories: processing aid (Fig. 1f), modified oil (Fig. 1h), protein source (Fig. 1i), and fibre (Fig. 1j).

#### Association of specific MUPs with all-cause mortality

**Fig. 2:**
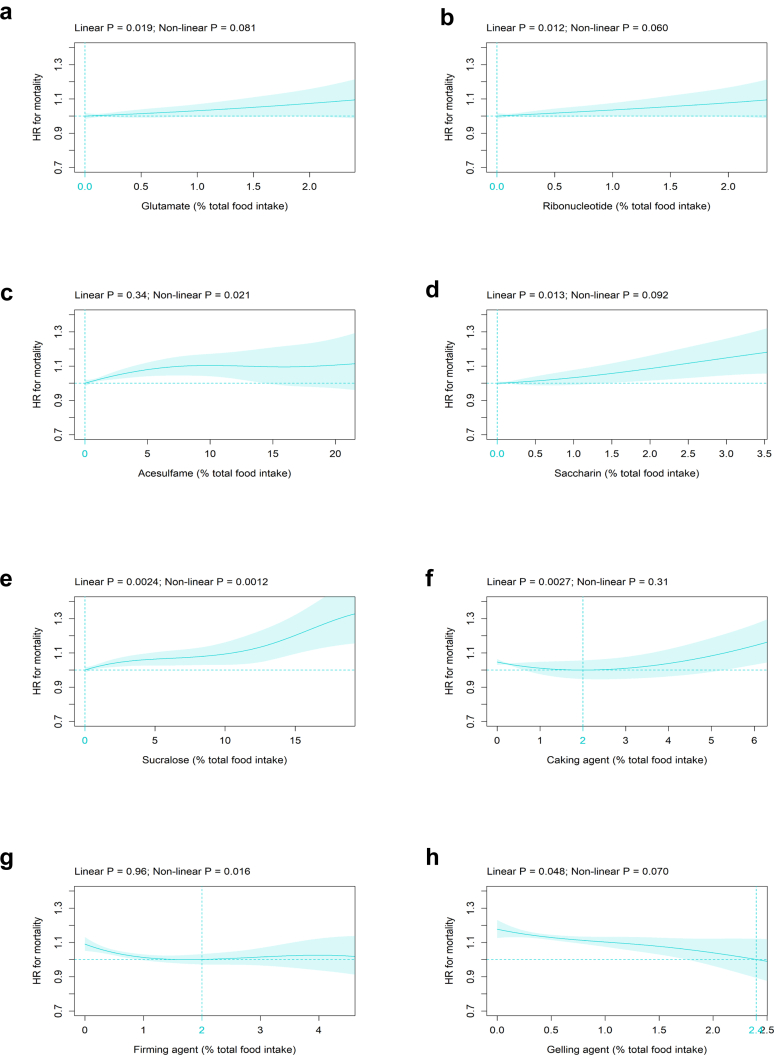

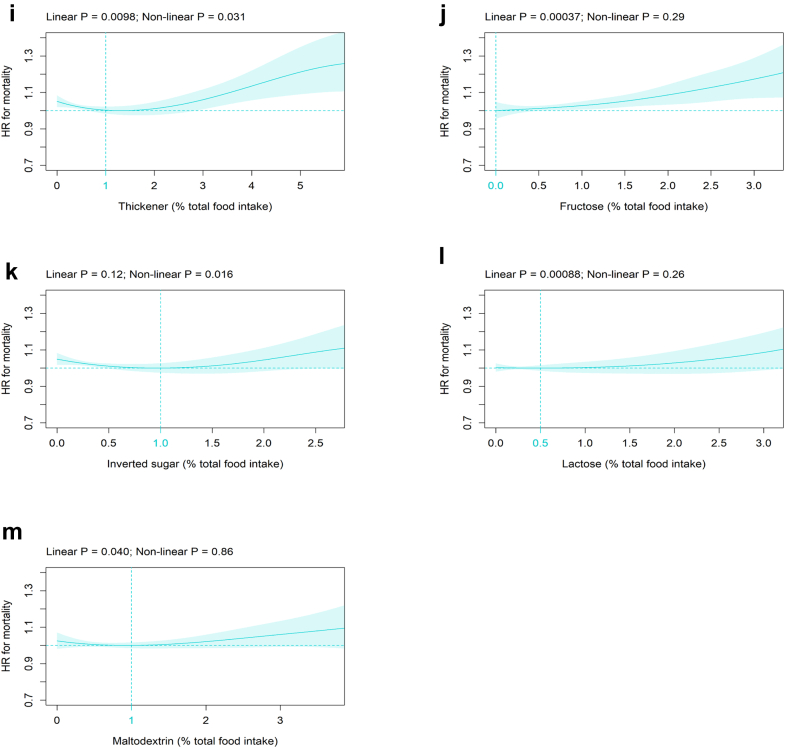
Association of specific MUPs with all-cause mortality. Association of the specific MUPs (all %TFI) (a) glutamate, (b) ribonucleotide, (c) acesulfame, (d) saccharin, (e) sucralose, (f) caking agent, (g) firming agent, (h) gelling agent, (i) thickener, (j) fructose, (k) inverted sugar, (l) lactose, and (m) maltodextrin with all-cause mortality (n = 186,744; number of events = 10,203). Models are adjusted as defined in Fig. 1 and abbreviations are indicated in Fig. 1.

Of the 57 MUPs, 37 MUPs were present in at least one food item. Since the four specific MUPs flavour, colour, hydrogenated oil, and fibre were identical to their respective MUP categories (Supplementary Table S1), the results have already been presented in Fig. 1b (flavour category = flavour), 1d (colouring agent category = colour), 1h (modified oil category = hydrogenated oil), and 1j (fibre category = fibre). Of the remaining 33 specific MUPs, 13 MUPs were significantly associated with all-cause mortality (Fig. 2). Within the flavour enhancer category, glutamate (Fig. 2a) and ribonucleotide (Fig. 2b) showed a significant linear relation with all-cause mortality and a HR-nadir at 0 %TFI. In the sweetener category, three of the ten specific MUPs, i.e., acesulfame (Fig. 2c), saccharin (Fig. 2d), and sucralose (Fig. 2e), were significantly related to all-cause mortality with a HR-nadir at 0 %TFI. Among the processing aid category, four out of ten specific MUPs showed a significant association with all-cause mortality, i.e., caking agent (Fig. 2f), firming agent (Fig. 2g), gelling agent (Fig. 2h) and thickener (Fig. 2i) with a HR-nadir between 1 and 2.4 %TFI. Among these, gelling agent was inversely associated with mortality risk (Fig. 2h). Within the varieties of sugar category, four of the five specific MUPs, i.e., fructose (Fig. 2j), inverted sugar (Fig. 2k), lactose (Fig. 2l), and maltodextrin (Fig. 2m) were significantly related to all-cause mortality with a HR-nadir between 0 and 1 %TFI. The remaining 20 MUPs were not significantly associated with all-cause mortality (Supplementary Fig. S4).

### Sensitivity analyses

Major results of all sensitivity analyses are summarised in Supplementary Table S5.

The associations between UPF intake and mortality risk (Supplementary Figs. S5a–S18a), as well as those for the categories flavour (Supplementary Figs. S5b–S18b), colouring agent (Supplementary Figs. S5d–S18d), and sweetener (Supplementary Figs. S5e–S18e), remained significant in all sensitivity analyses. For the categories flavour enhancer (Supplementary Figs. S5c–S18c) and varieties of sugar (Supplementary Figs. S5g–S18g) most associations remained significant. Of the 13 specific MUPs which were significantly associated with mortality risk in the main analyses (Fig. 2), saccharin (Supplementary Figs. S5n–S18n), sucralose (Supplementary Figs. S5o–S18o), and fructose (Supplementary Figs. S5t–S18t) remained significant in all sensitivity analyses. Associations of all other MUPs were significant in some but not all sensitivity analyses (Supplementary Figs. S5–S18 and Table S5). All associations remained significant for UPF, as well as the MUP categories flavour, colouring agent, and sweetener if the MLI calculation was based on eight, six, or four commercial products (Supplementary Fig. S19). Furthermore, there was a significant association between cumulative specific MUP intake and mortality risk (Supplementary Fig. S20a). This association remained significant in all sensitivity analyses (Supplementary Fig. S20b–f). Non-significant associations in the main analysis remained non-significant in the majority of sensitivity analyses (Supplementary Table S6). Association with mortality risk in the main analysis remained significant after adjustment for multiple testing for the MUP categories flavour, flavour enhancer, colouring agent, sweetener, and varieties of sugar, as well as the specific MUPs sucralose, caking agent, fructose, and lactose (Supplementary Table S7).

## Discussion

The present exploratory study is the first to assess the association between the intake of foods containing MUP categories, specific MUPs, and MUP-based definition UPF with all-cause mortality. It is shown that flavour, flavour enhancer, colouring agent, sweetener, and varieties of sugar are the MUP categories which are significantly and positively associated with the risk of death. Moreover, consumption of UPF is also strongly associated with all-cause mortality. Furthermore, 13 specific MUPs including glutamate, saccharin, sucralose, and fructose are significantly linked with all-cause mortality. Intake of foods containing the specific MUP gelling agent is inversely associated with mortality risk. This negative association may be partly explained by the use of pectin, a fibre-based gelling agent that may have potential health benefits.^33^

To the best of our knowledge, no prior study has assessed associations of such a broad range of MUP categories and specific MUPs with all-cause mortality. However, adverse health effects have been described for flavour, flavour enhancer, colouring agent, sweetener, and varieties of sugar: Thus, our group has recently suggested that flavour is an important contributor to body weight gain and obesity via two independent mechanisms, i.e., it promotes hedonic eating and disrupts flavour-nutrient learning.^15^ These mechanistic studies are supported by animal data showing that added flavour increases feed intake and body weight as compared to non-flavoured control diets.^15^ Moreover, the flavour enhancer monosodium glutamate has been linked to increased food intake and body weight gain in some but not all animal and human studies.^34^ Mechanisms for adverse health effects of monosodium glutamate include impaired hypothalamic leptin signalling and changes in the gut microbiota.^34^ Synthetic food colouring agent influences the sensory appeal and encourages preference for certain foods.^16^ Furthermore, colouring agent is directly linked to a series of adverse health outcomes including behavioural changes such as Attention Deficit Hyperactivity Disorder and Autism Spectrum Disorder, as well as carcinogenic and allergenic effects.^16^ Sweetener interferes with basic learned, predictive relations between sweet taste and the post-ingestive consequences.^17^ Animal studies in both the agricultural sector and the laboratory indicate that this interference leads to impaired regulation of food intake with compensatory overeating and body weight gain contributing to an increased risk for obesity and associated negative health outcomes.^17^ Specific MUPs of the sweetener category also drive the development of glucose intolerance through induction of compositional and functional alterations to the intestinal microbiota in human subjects.^35^ The varieties of sugar category consists of free sugars which have been linked to body weight gain and obesity.^36^

For some specific MUPs which have been linked to adverse health outcomes including emulsifier^18^ and gluten,^19^ no significant association with all-cause mortality is found in the current analysis. It is important to note in this context, that MUP-related health risks may not be solely attributable to the specific MUPs themselves but rather to their interactions within the broader food and processing procedures. Thus, the adverse effects of UPF may reflect a combination of factors including matrix degradation, certain processing methods, nutrient and energy content, as well as behavioural aspects such as increased portion sizes and low costs.^37^ Further studies are needed to clarify these mechanisms.

Our study is the first to define UPF with a MUP-based approach. The significant linear association between UPF consumption and mortality found in the current study is broadly consistent with various reports in which UPF is defined based on published food lists. Thus, Lane and co-workers demonstrate convincingly in an umbrella review of existing meta-analyses that UPF intake is not only associated with a broad range of metabolic, vascular, and psychological diseases but also with an increased risk of all-cause mortality.^3^

This study has several limitations. These include potential residual confounding, measurement errors in exposure assessment, and the influence of unmeasured variables that may have biased the results. In addition, there is potential for confounding by correlated food intake. However, adjustment for diet quality does not substantially alter the results. As no formal sample size calculation was conducted prior to this exploratory analysis, statistical power for less common specific MUPs may be limited despite the overall large cohort size. Furthermore, all consumption data are self-reported and not independently verified. The Oxford WebQ does not assess food ingredient lists and, therefore, the MLI is based on the probability of a MUP being present in each of the 206 food and 32 beverage items. Nevertheless, this MUP-based approach offers greater objectivity and reproducibility compared to the classification of foods based on predefined food lists, as commonly employed in epidemiological studies on UPF to date. Moreover, a selection bias known as the “healthy volunteer” bias might exist as the cohort does not accurately represent the demographic composition of the general UK population.^38^ However, a representative population is not a prerequisite for establishing the relationships between exposure and disease.^38^

Taken together, the current study is the first to comprehensively investigate the association of foods containing MUP categories and specific MUPs, as well as a MUP-based definition of UPF, with all-cause mortality. The results indicate that MUP-based definition UPF, as well as some MUPs including flavour, flavour enhancer, colouring agent, sweetener, and varieties of sugar, are positively linked to all-cause mortality whereas processing aid, modified oil, protein source, and fibre have no significant association with this endpoint. The findings provide an initial framework for identifying MUPs that should be prioritised in interventions targeting UPFs and for future mechanistic studies. Future studies should replicate the observed associations between MUPs and mortality using the MLI applied in this study and explore links with other health outcomes, as well as analyse the underlying mechanisms.

## Contributors

KMK, GE, MF, and NJE conceived the research. NJE researched commercial products from the two market leaders of groceries in the UK and wrote the first draft of the manuscript. Statistical analyses were performed by all authors. All authors have read and approved the final version of the manuscript. All authors have verified the underlying data and take responsibility for the integrity of the data and the accuracy of the data analysis. The corresponding author attests that all listed authors meet authorship criteria and that no others meeting the criteria have been omitted.

## Data sharing statement

The data used in this study were accessed under institutional data use agreements with the UK Biobank (application number 53438). These agreements do not permit public sharing of the data. However, researchers can apply for access directly through the UK Biobank (www.ukbiobank.ac.uk) in accordance with their access procedures.

## Declaration of interests

Non declared.
