# Supplementary material for: Association of 37 markers of ultra-processing with all-cause mortality: a prospective cohort study in the UK Biobank
Source: eClinicalMedicine. 2025 Aug 26;88:103448. doi: 10.1016/j.eclinm.2025.103448 (PMC12572789; doi:10.1016/j.eclinm.2025.103448)
Supplement: Supplementary Figs. S1–S20 and Tables S1–S7 [file mmc1.pdf]

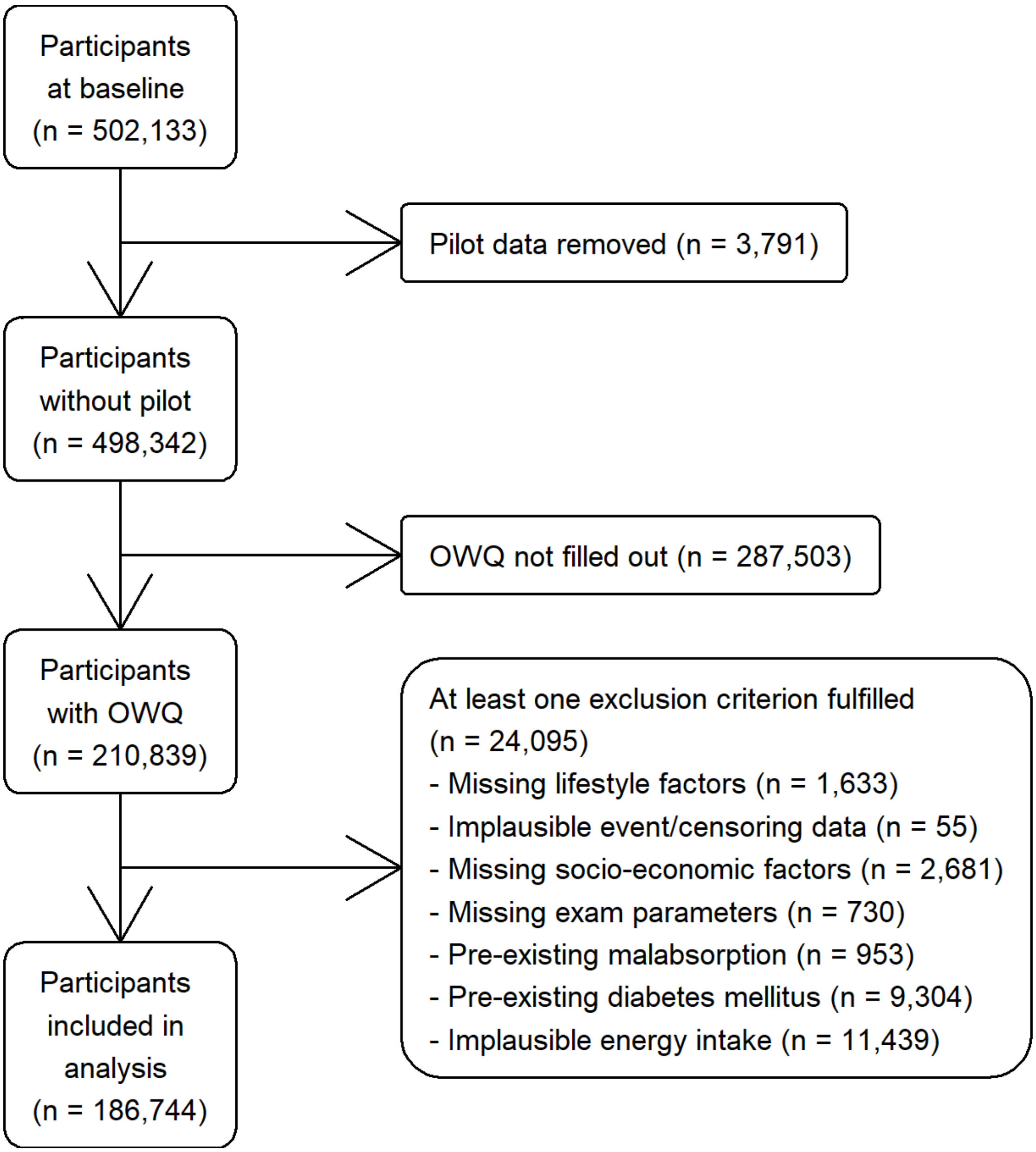

Fig. S1 - Flowchart of participant selection

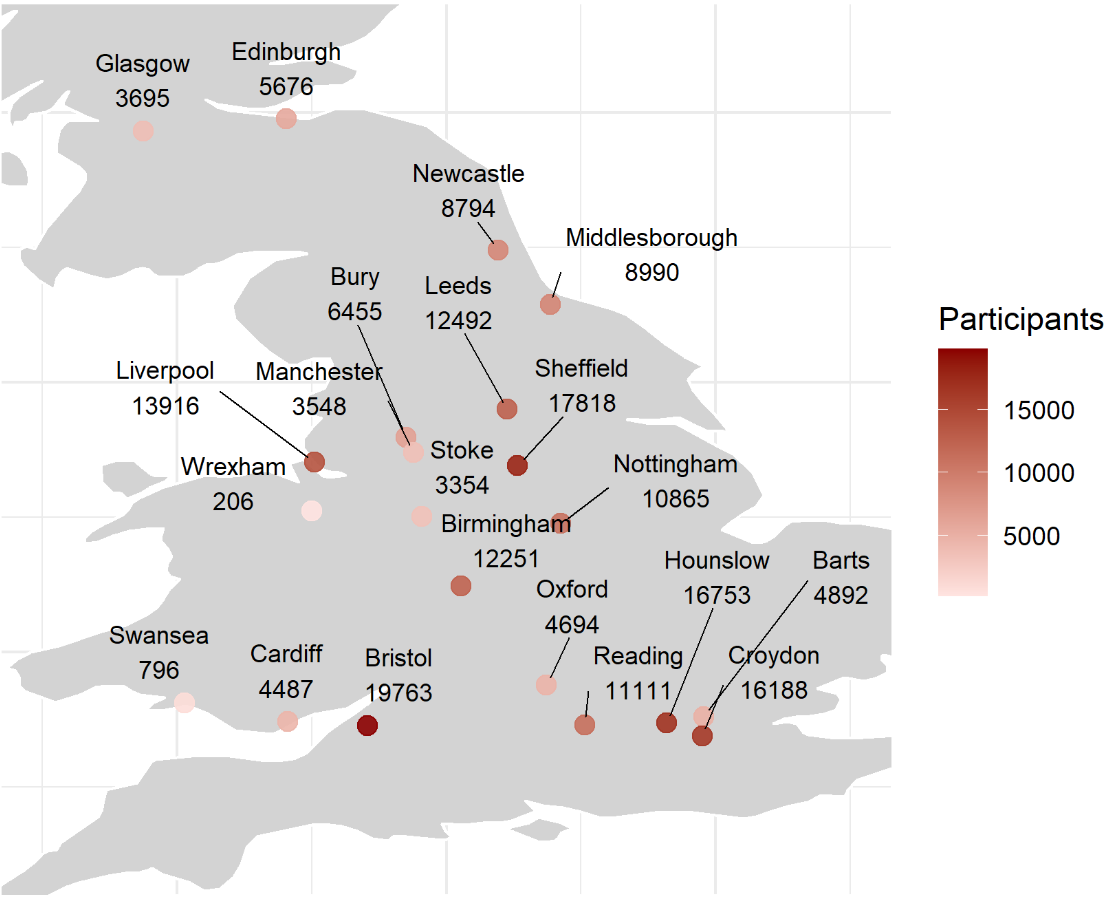

Fig. S2 - Participant counts by location of assessment centre

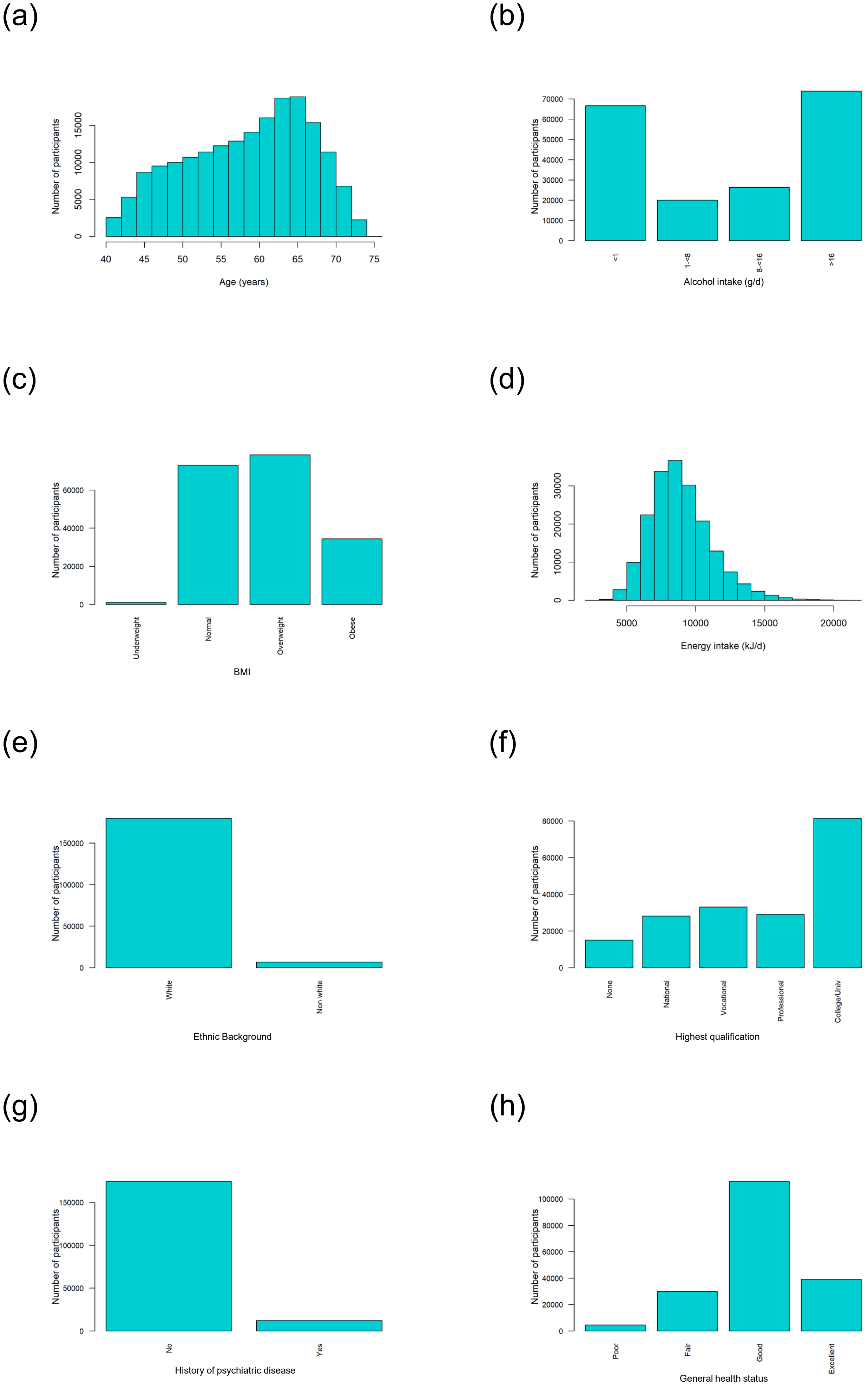

Fig. S3 - Distribution of covariates

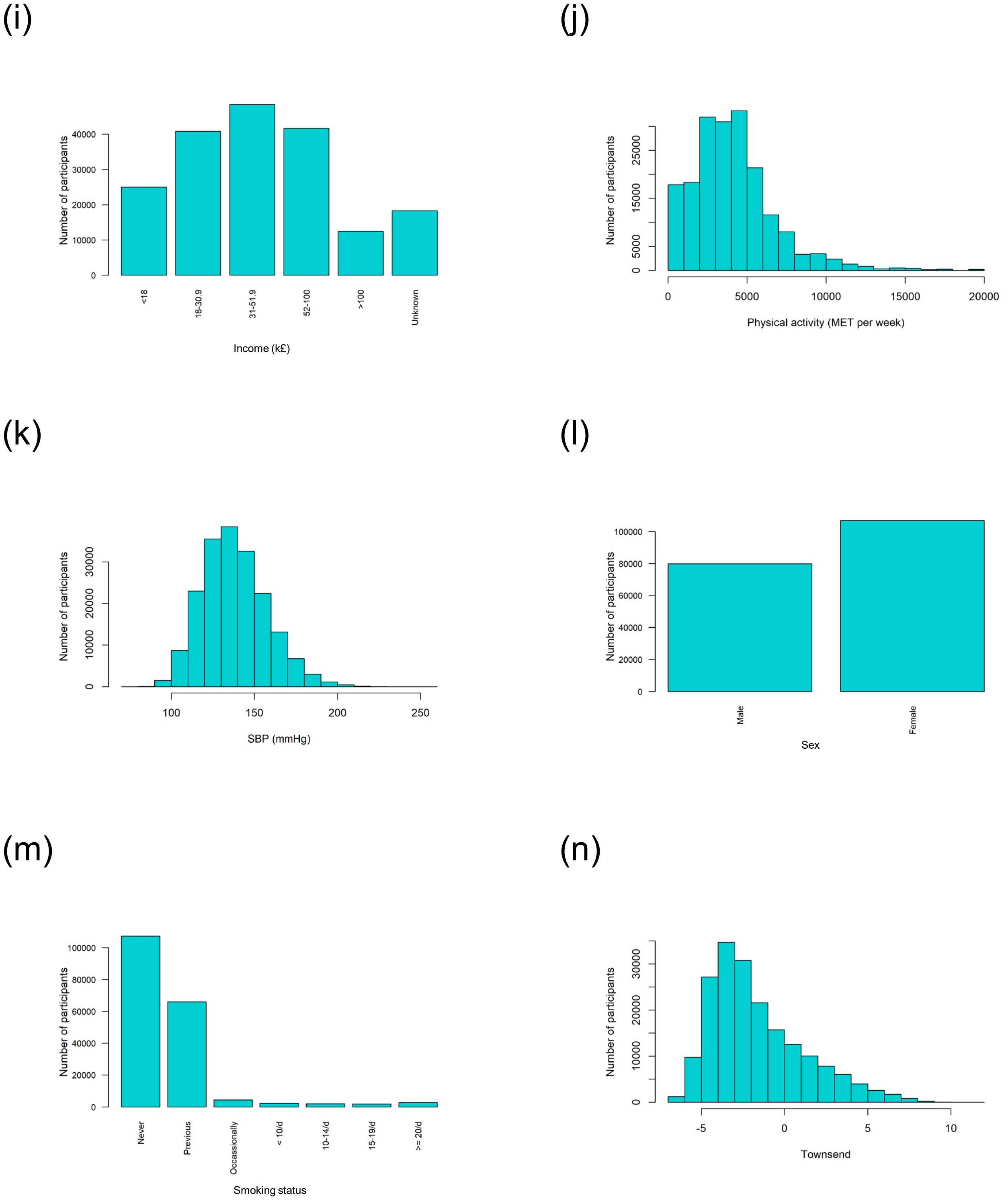

Fig. S3 - Distribution of covariates

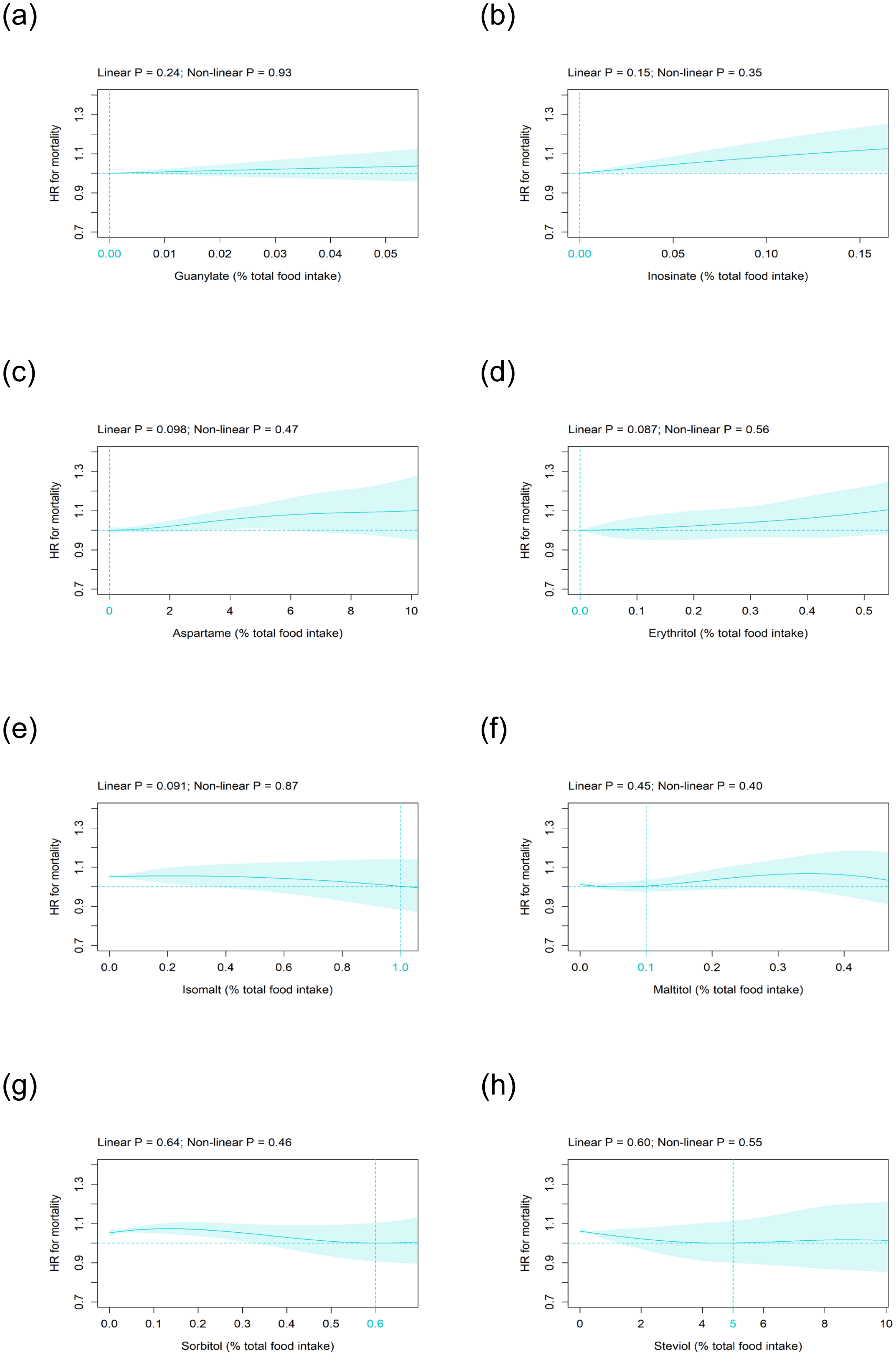

Fig. S4 - Non-significant associations of specific MUPs

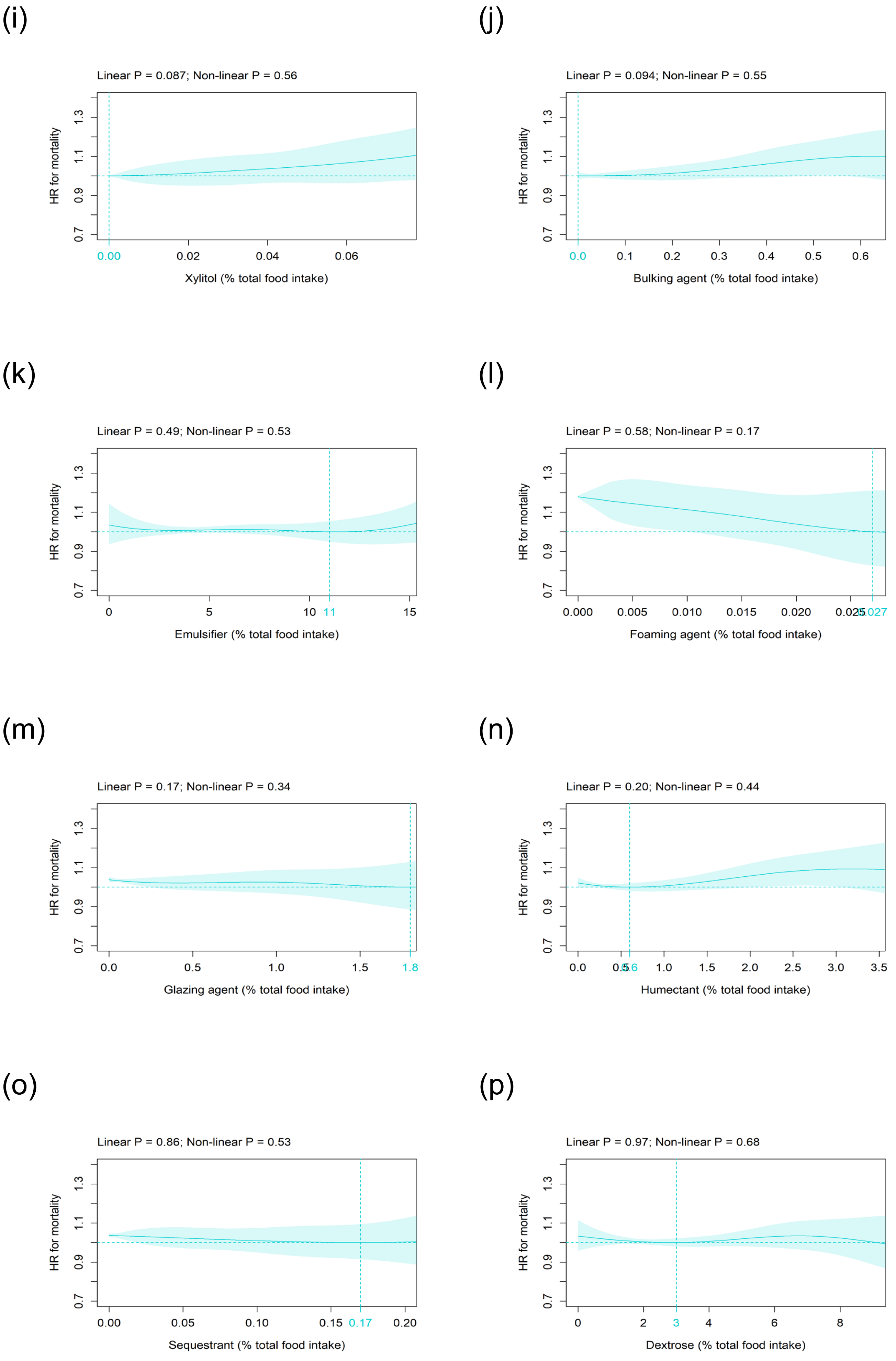

Fig. S4 - Non-significant associations of specific MUPs

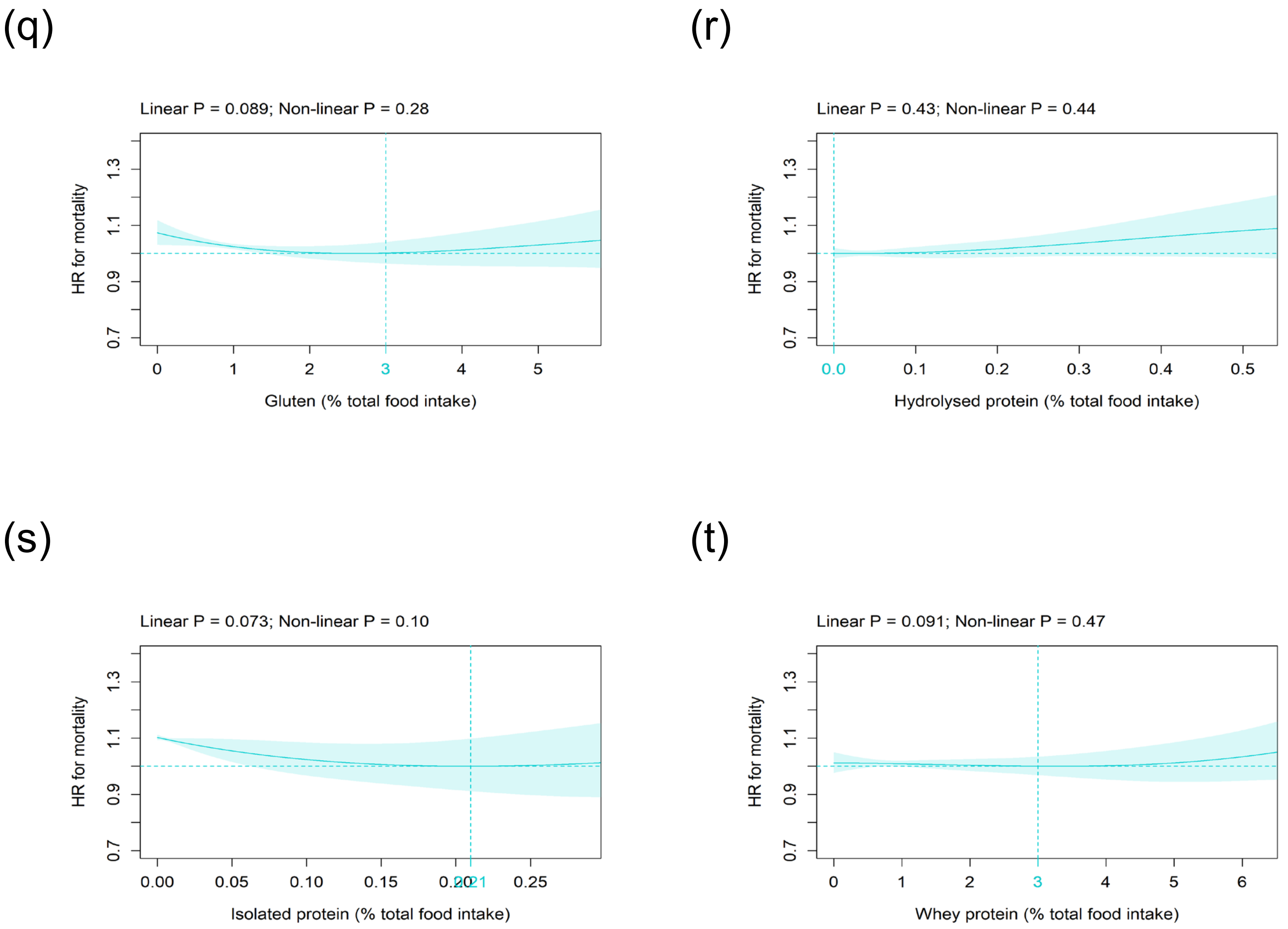

Fig. S4 - Non-significant associations of specific MUPs

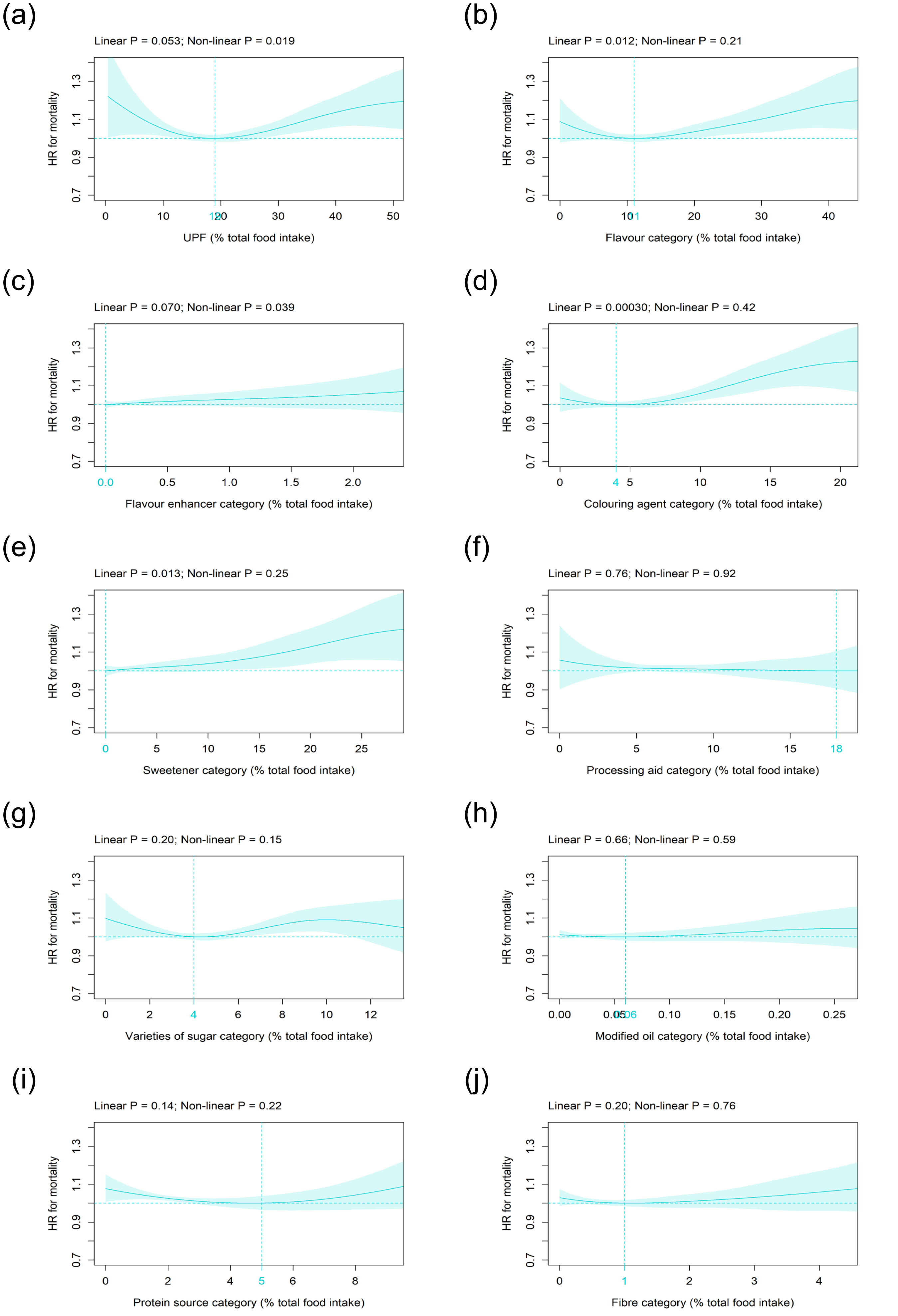

Fig. S5 - Landmark analysis

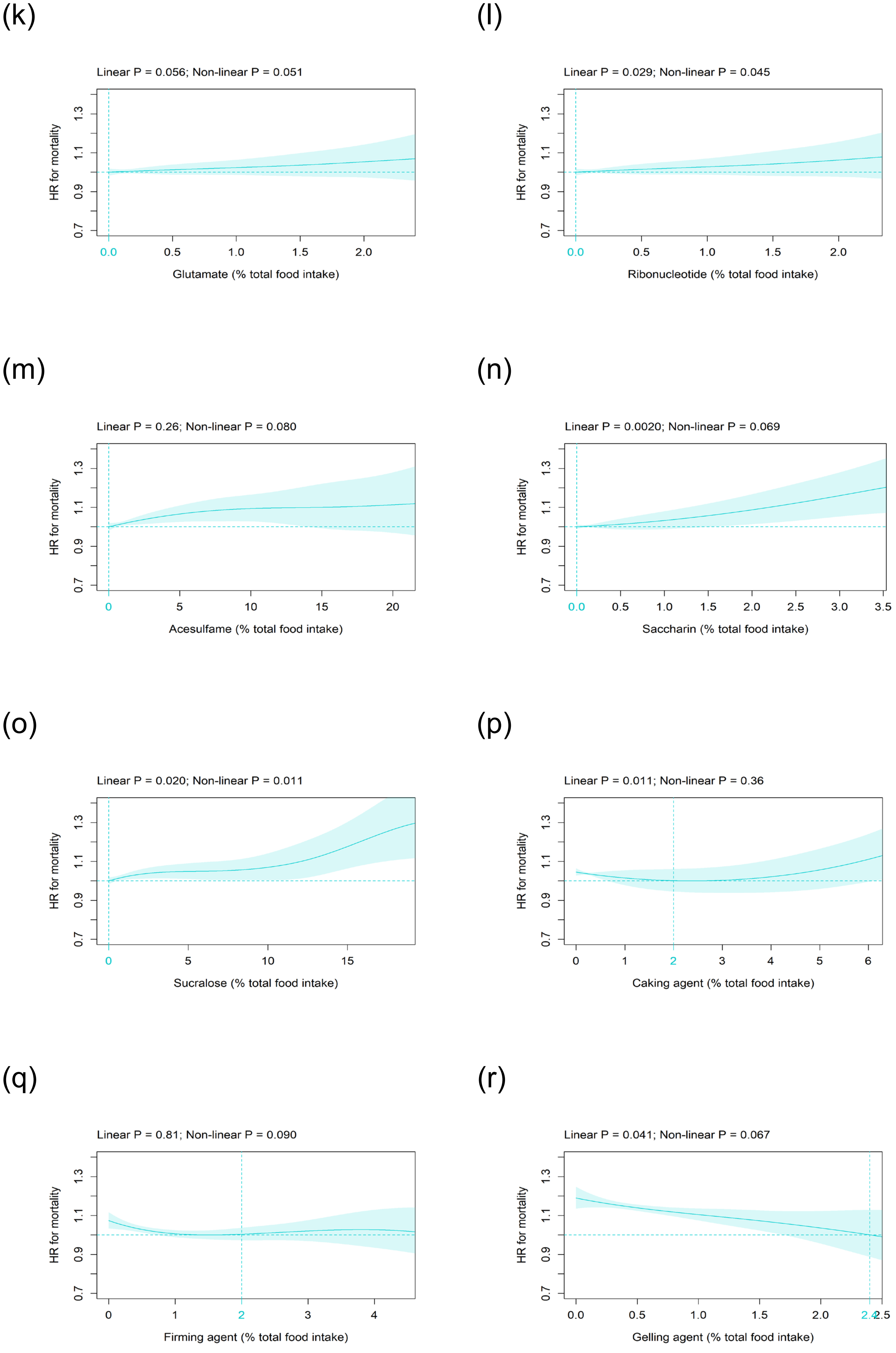

Fig. S5 - Landmark analysis

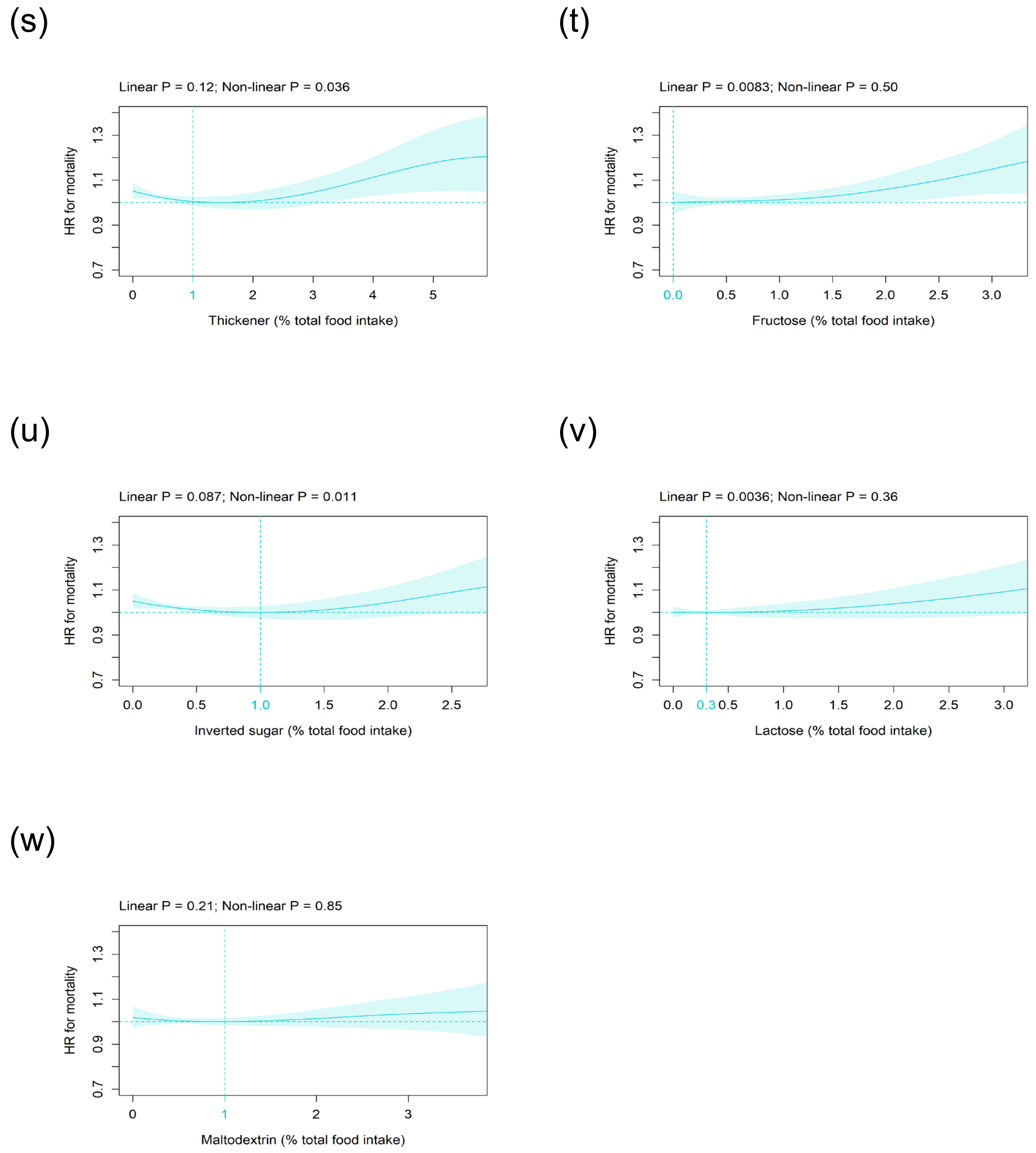

Fig. S5 - Landmark analysis

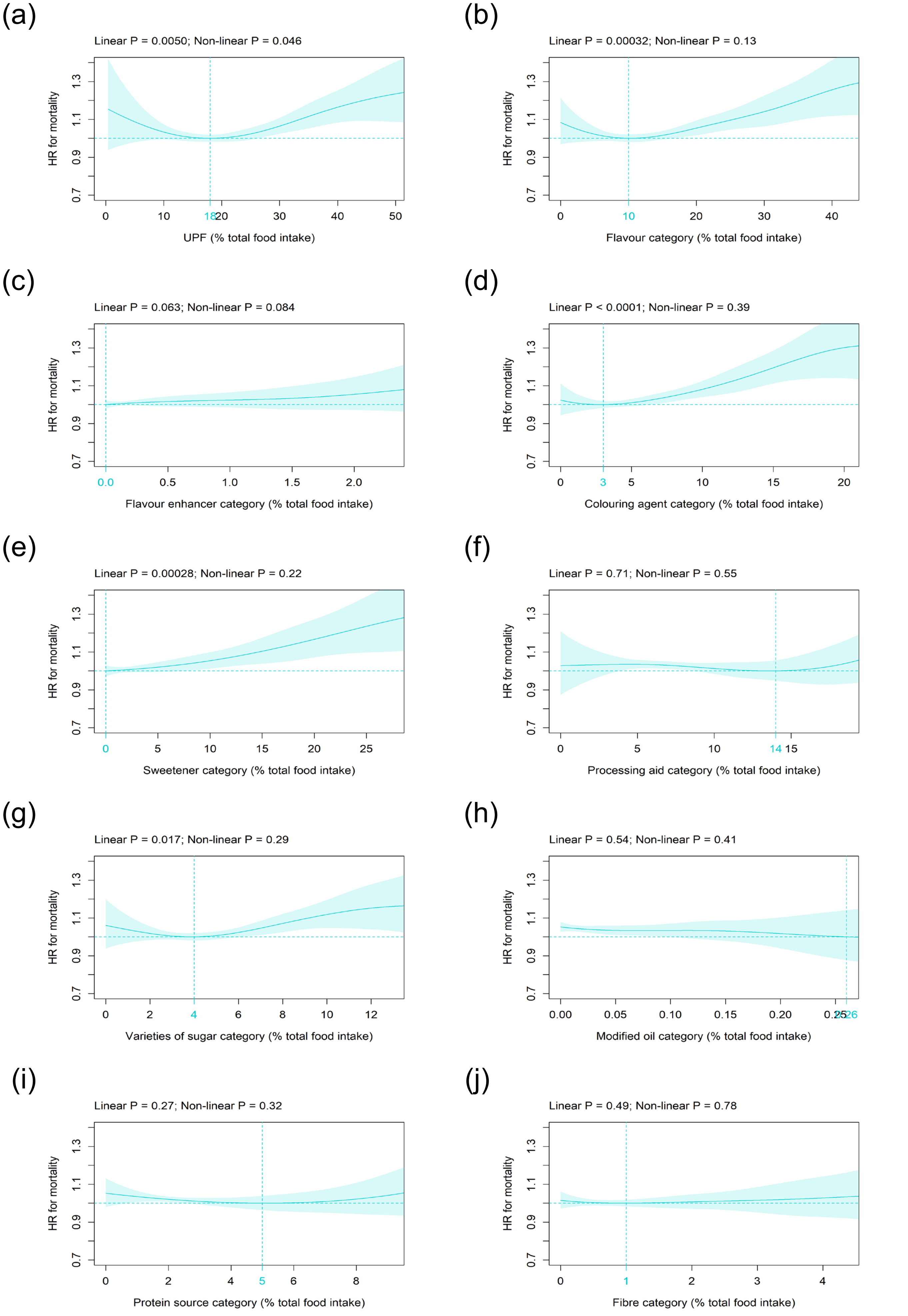

Fig. S6 - Unintentional weight loss removed

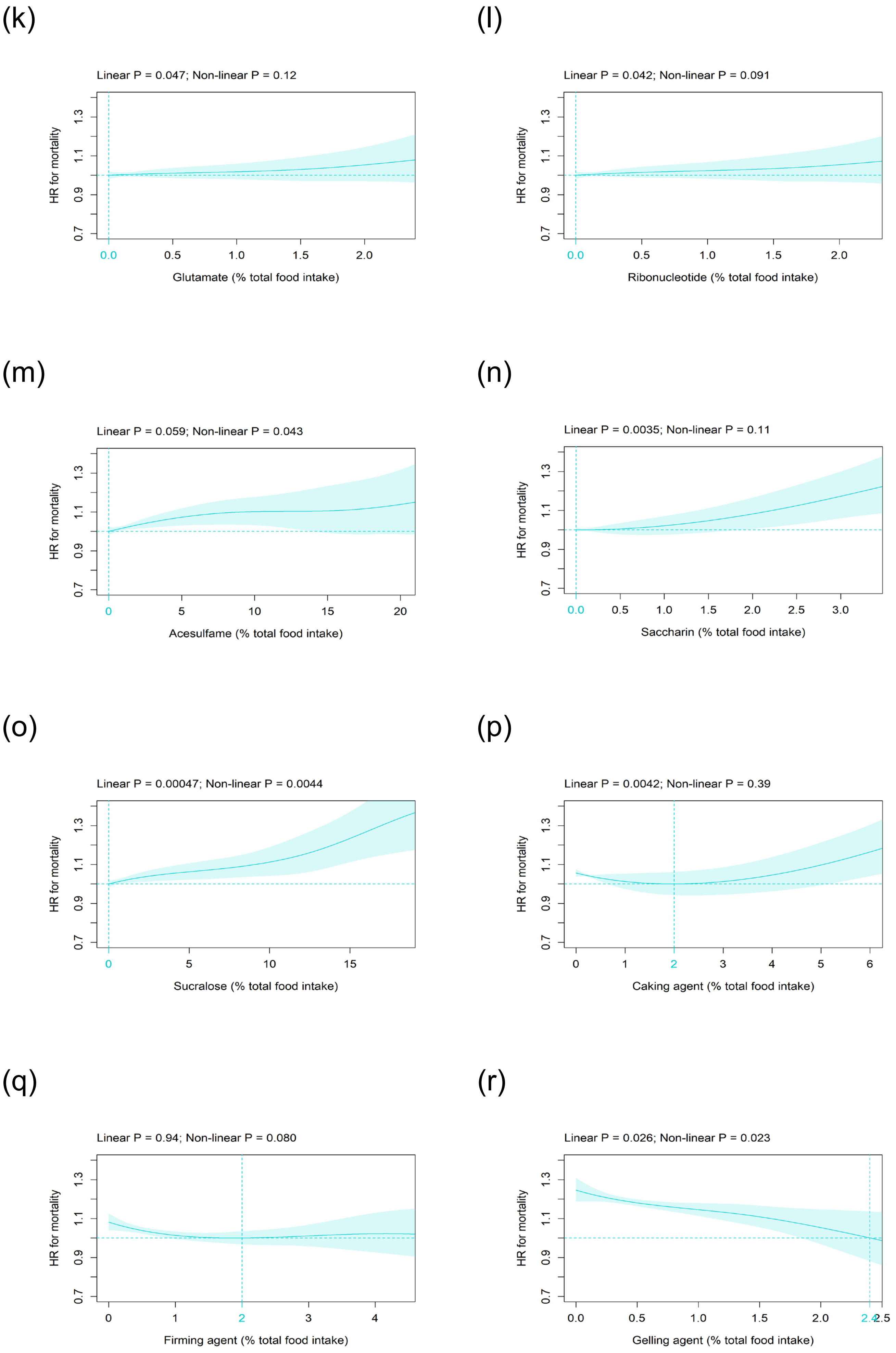

Fig. S6 - Unintentional weight loss removed

(s)

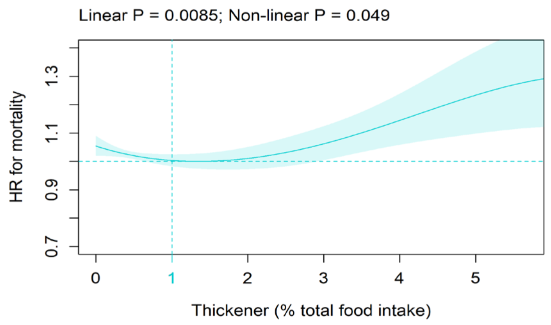

(t)

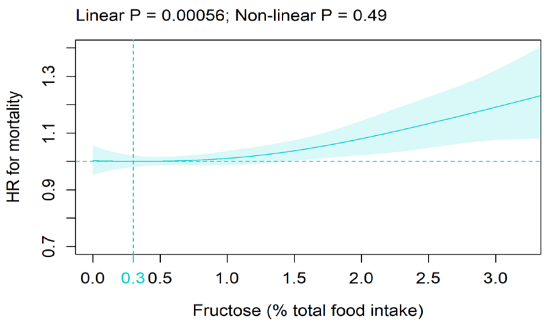

(u)

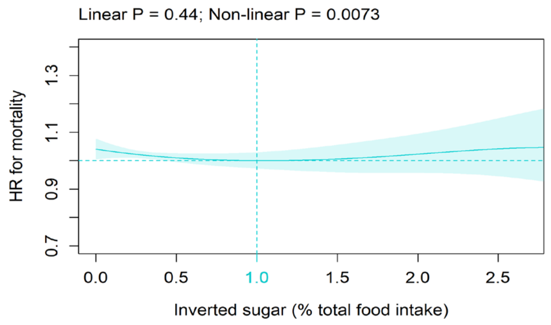

(v)

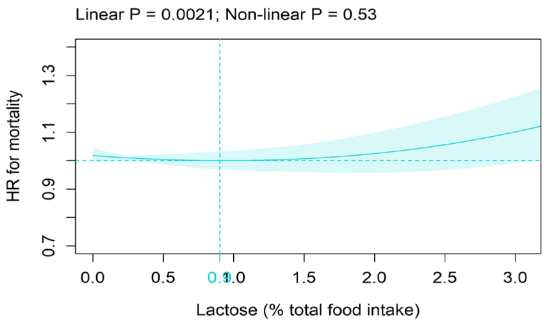

(w)

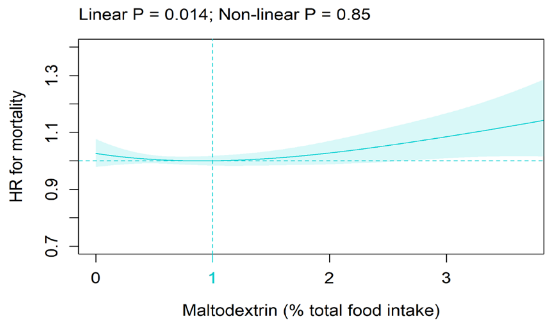

Fig. S6 - Unintentional weight loss removed

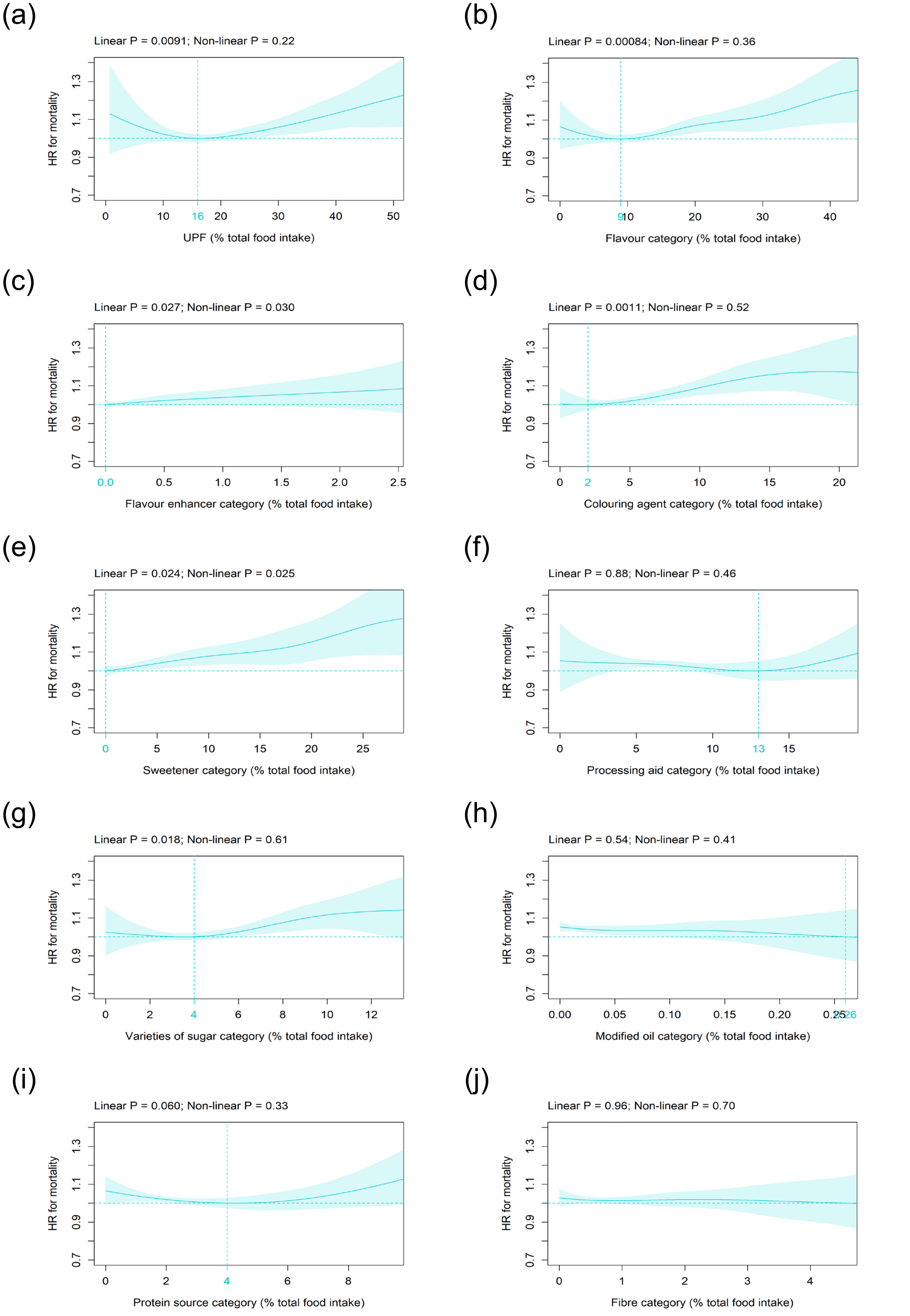

Fig. S7 - Atypical diet excluded

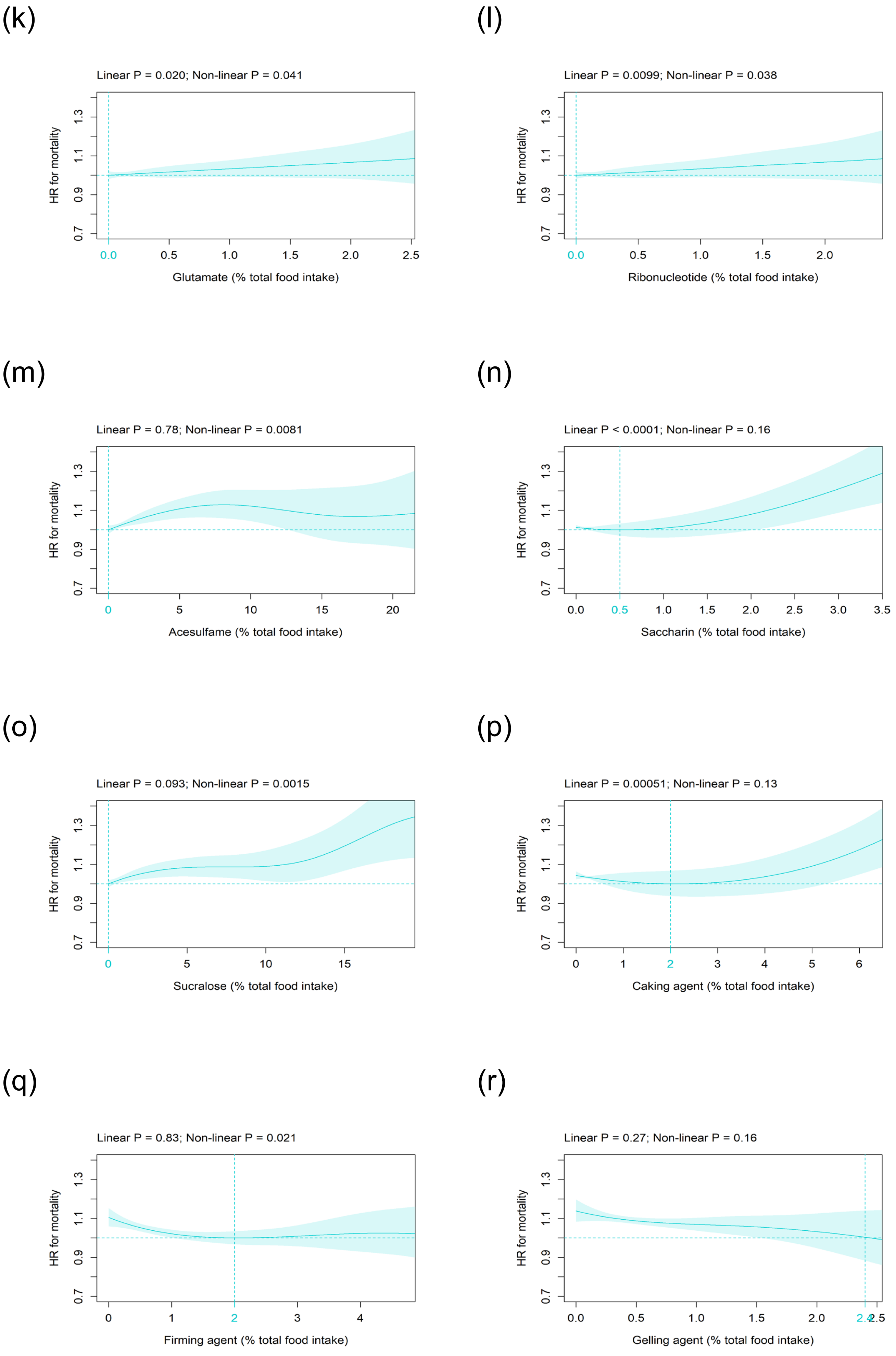

Fig. S7 - Atypical diet excluded

(s)

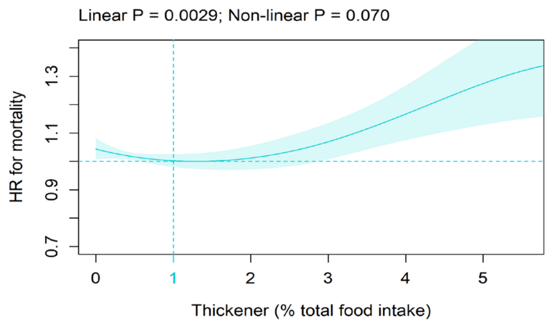

(t)

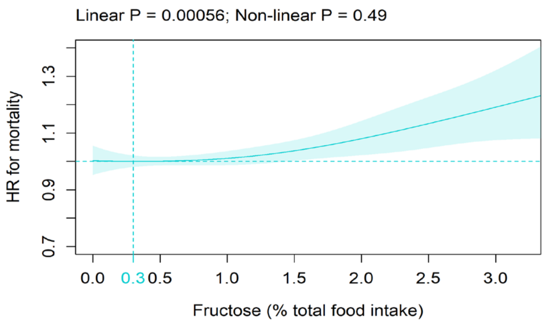

(u)

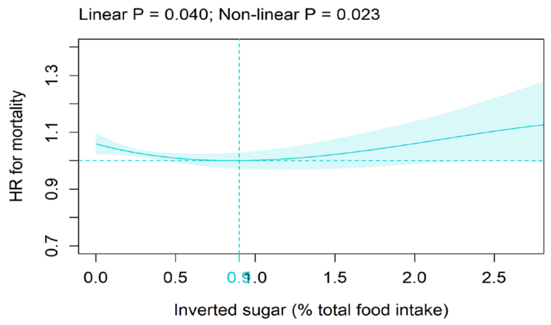

(v)

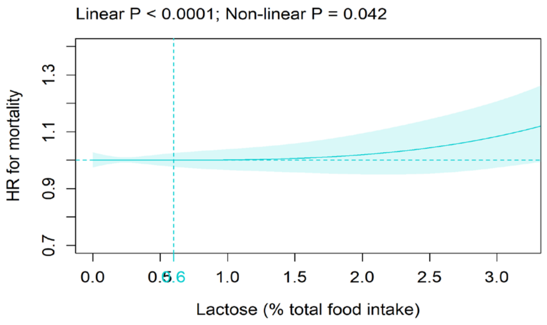

(w)

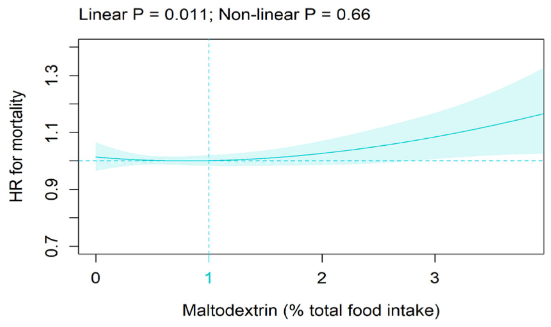

Fig. S7 - Atypical diet excluded

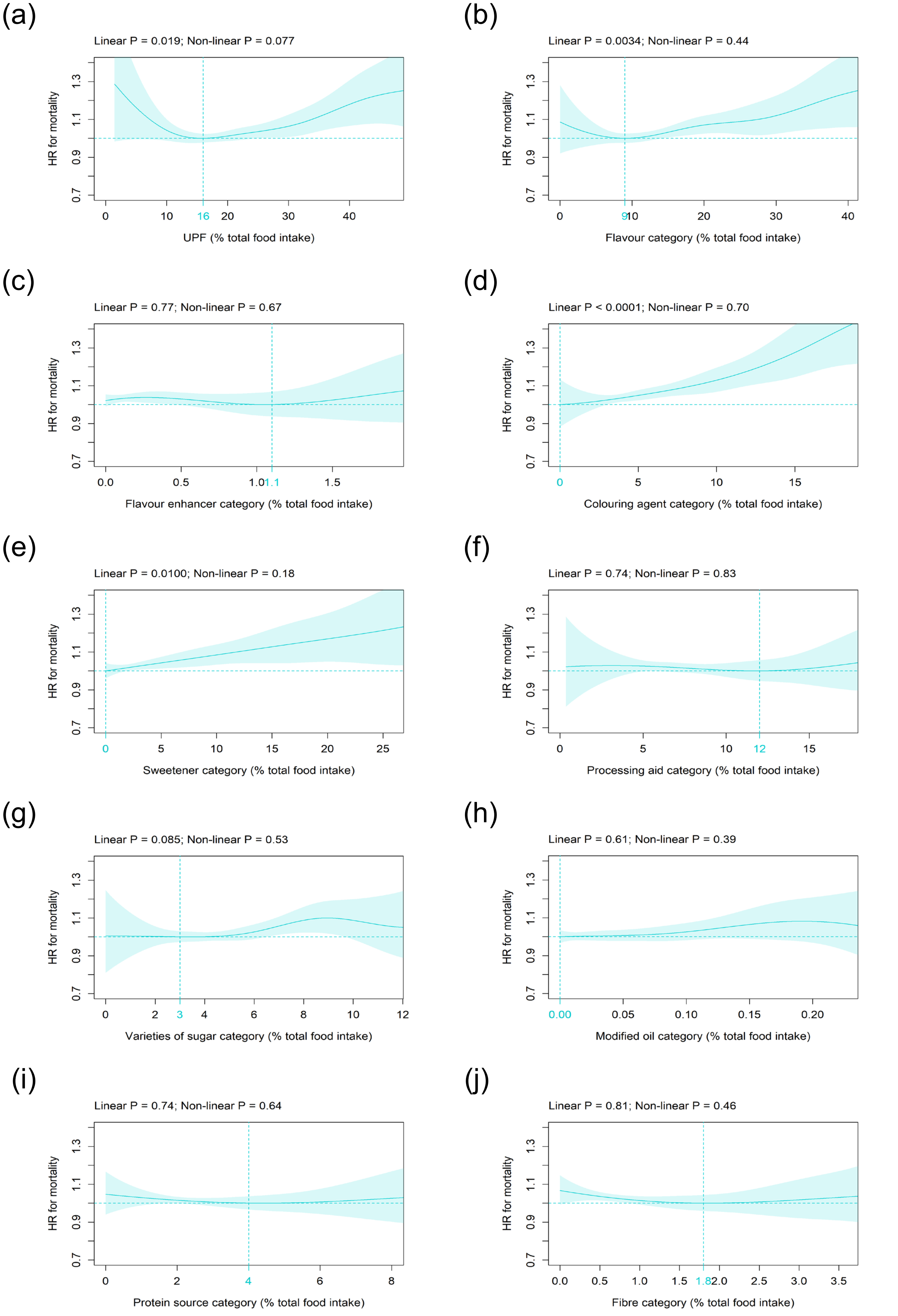

Fig. S8 - More than one Oxford WebQ

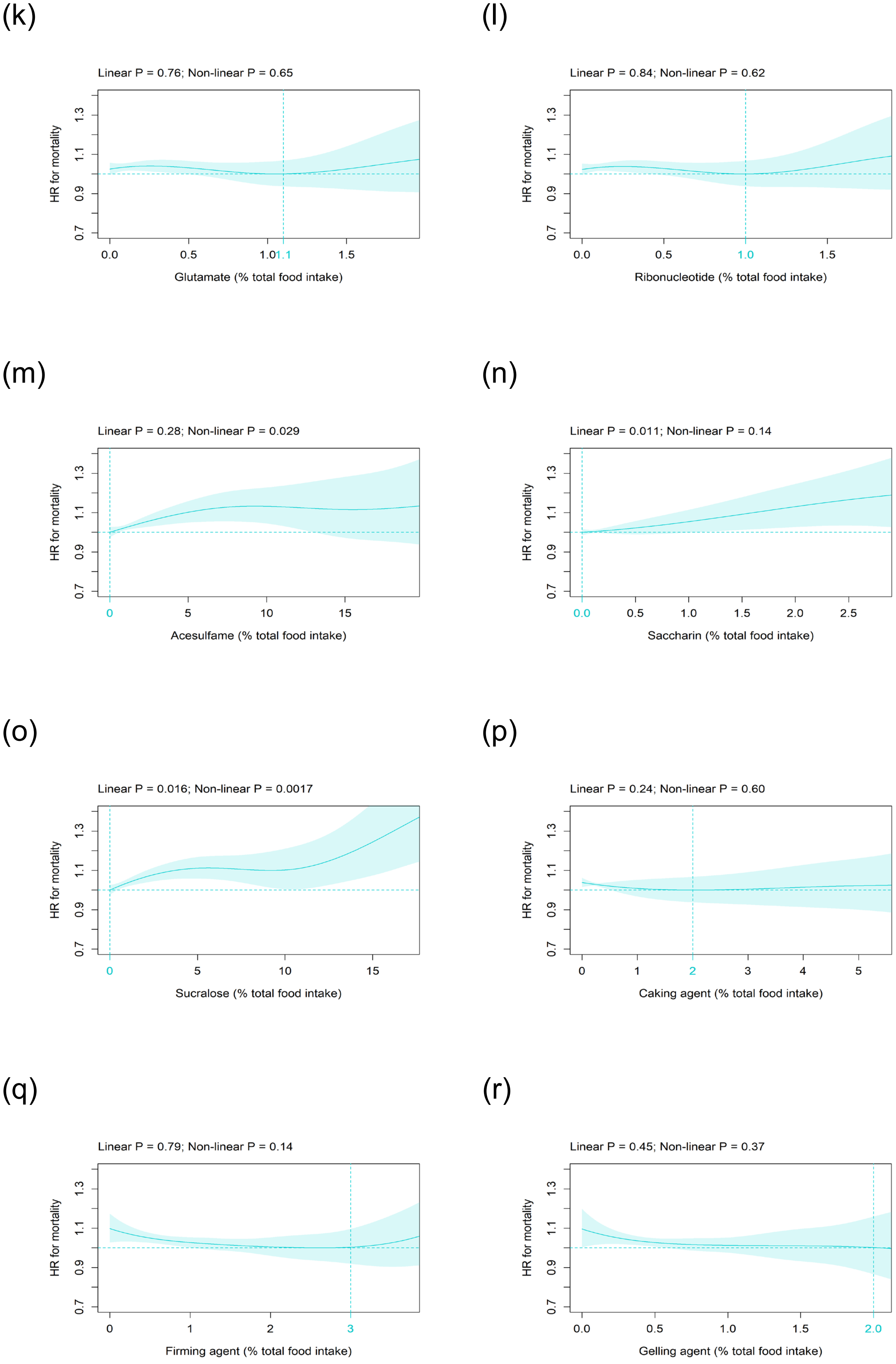

Fig. S8 - More than one Oxford WebQ

(s)

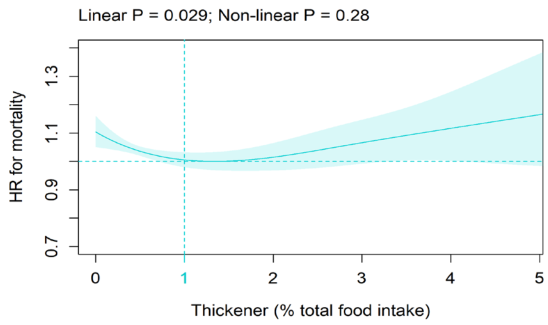

(t)

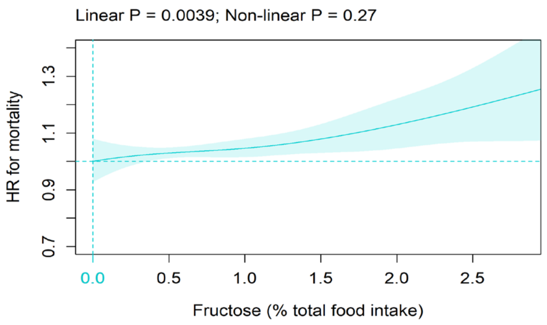

(u)

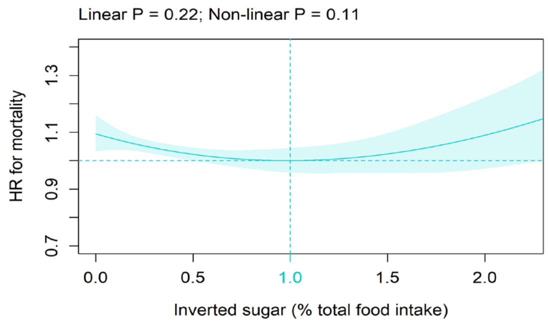

(v)

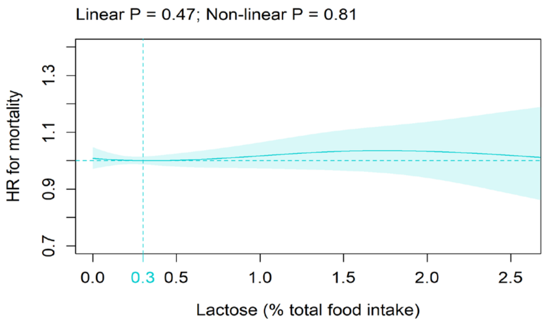

(w)

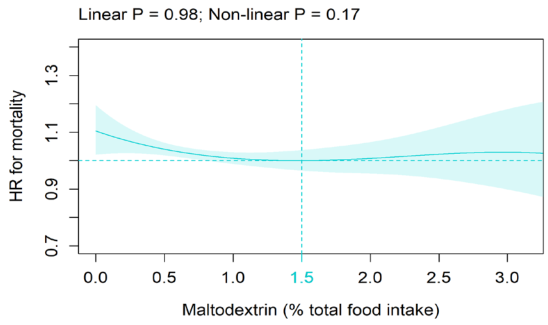

Fig. S8 - More than one Oxford WebQ

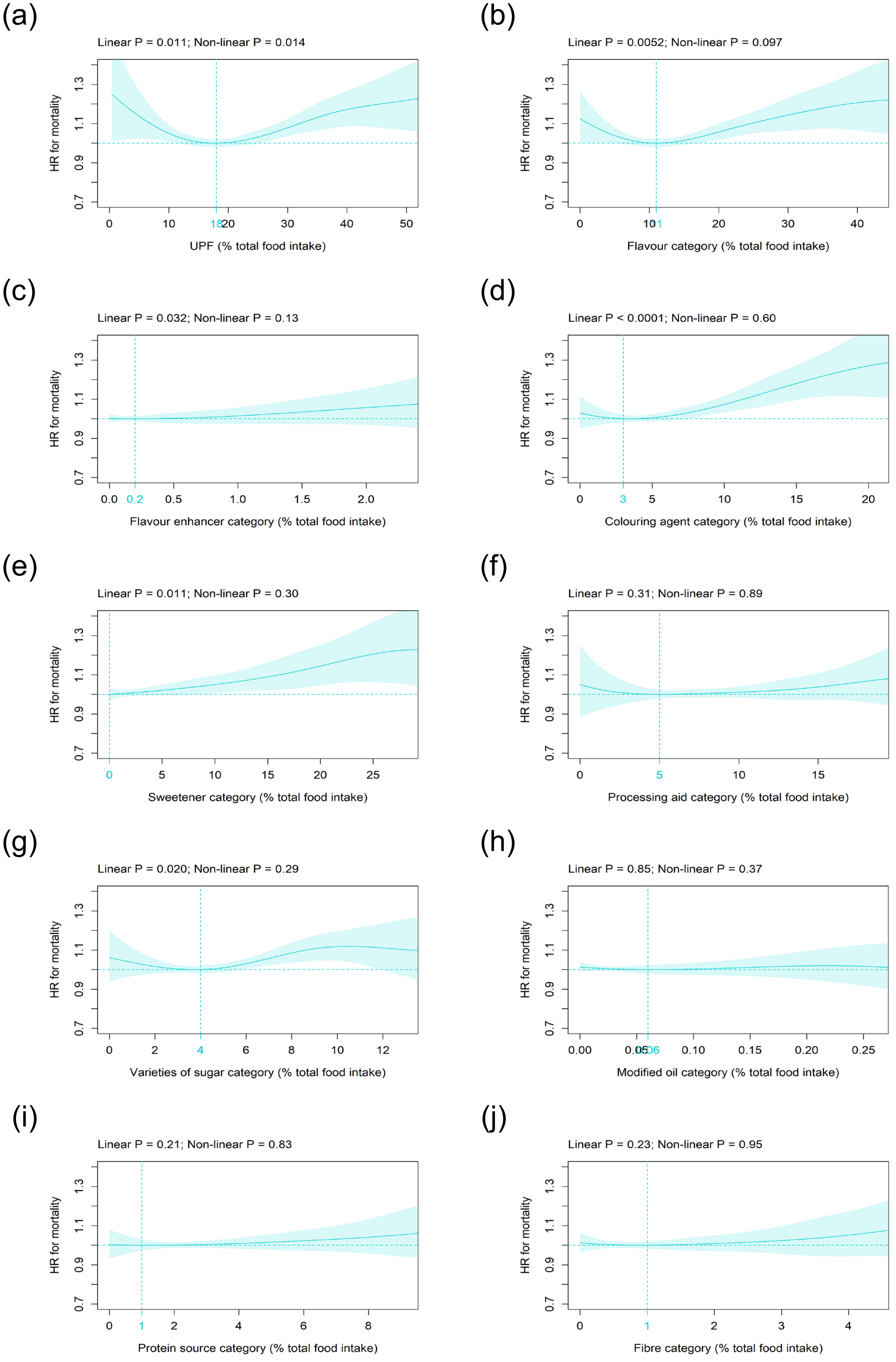

Fig. S9 - History of CVD and cancer excluded

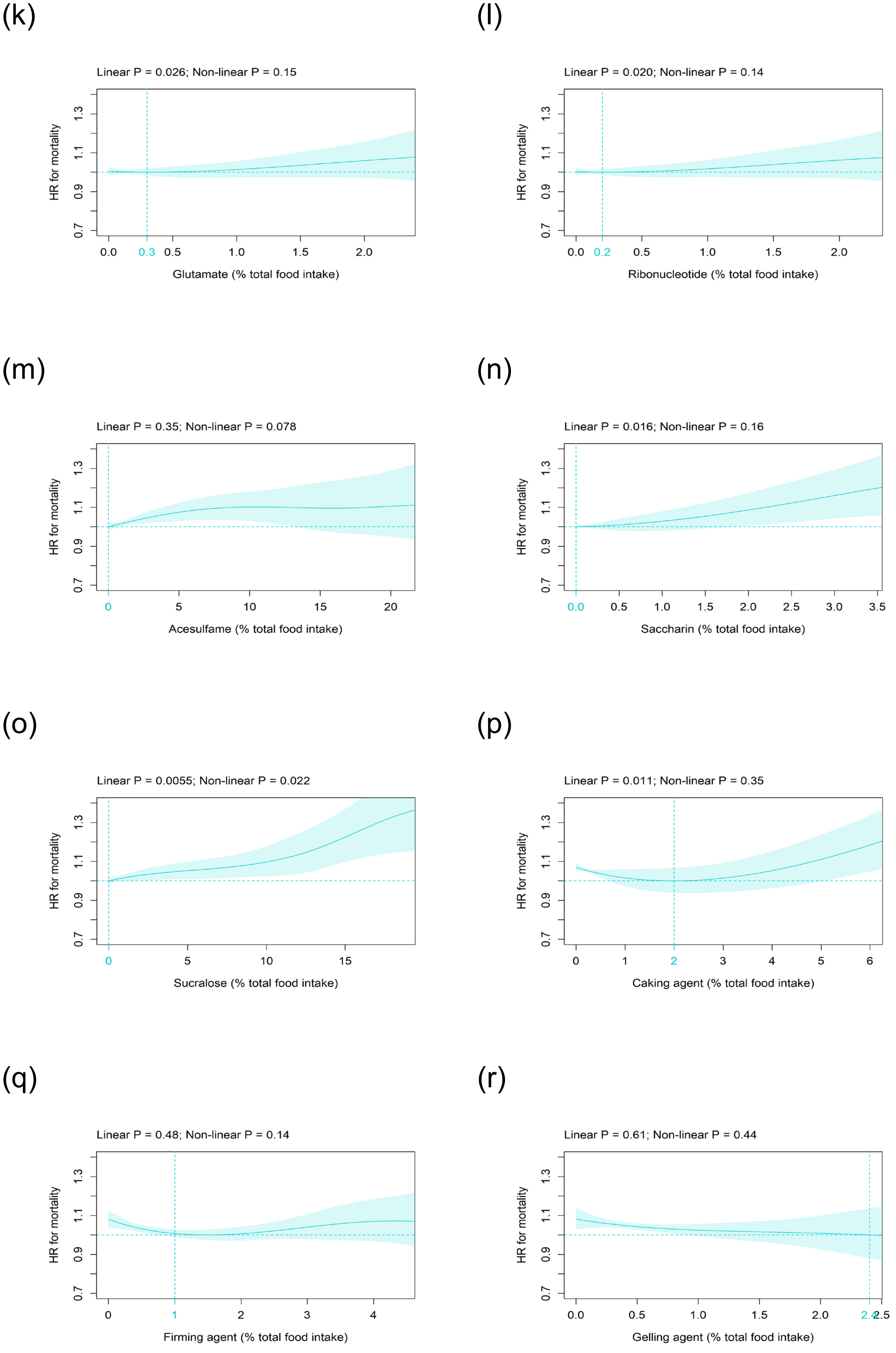

Fig. S9 - History of CVD and cancer excluded

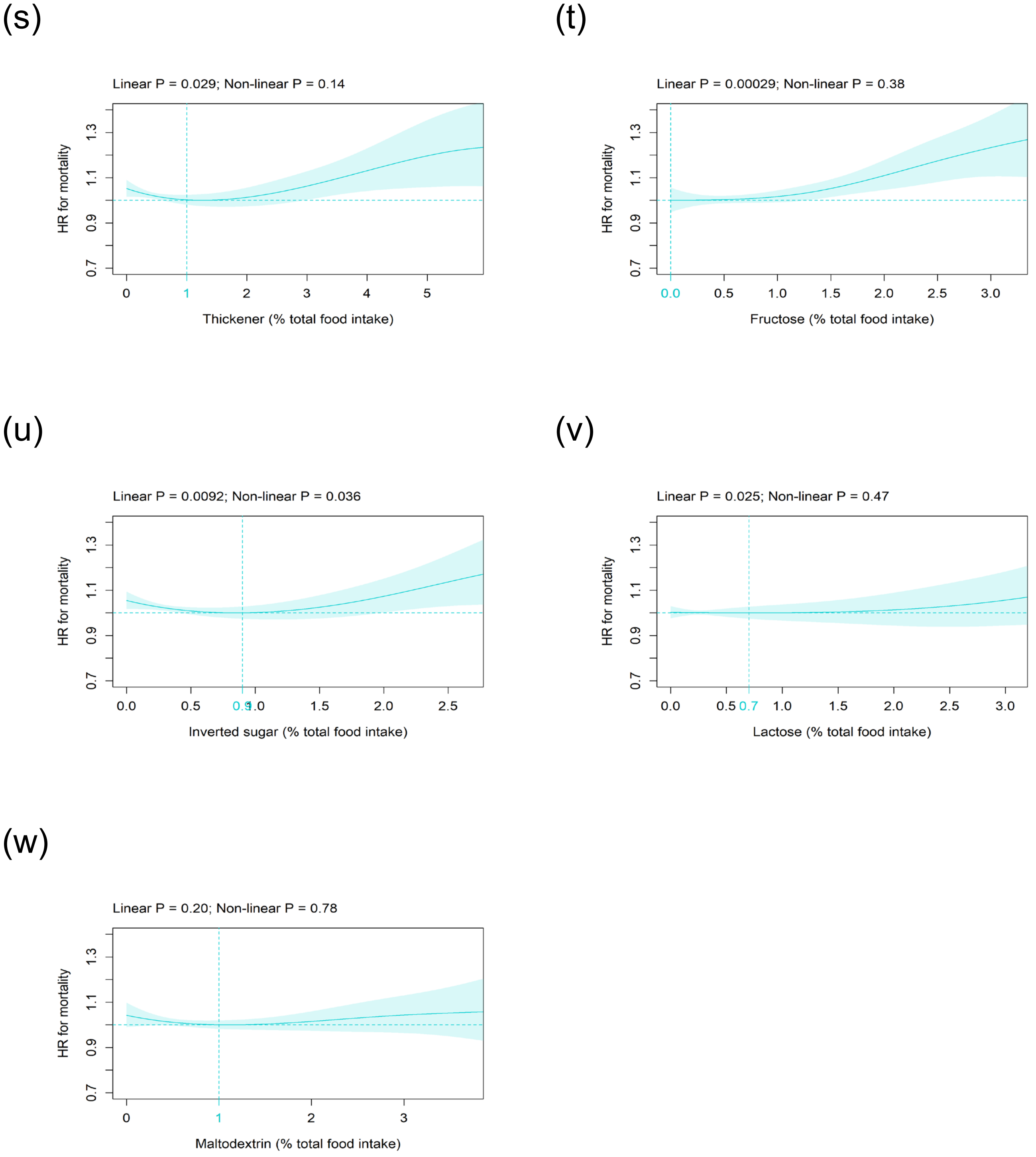

Fig. S9 - History of CVD and cancer excluded

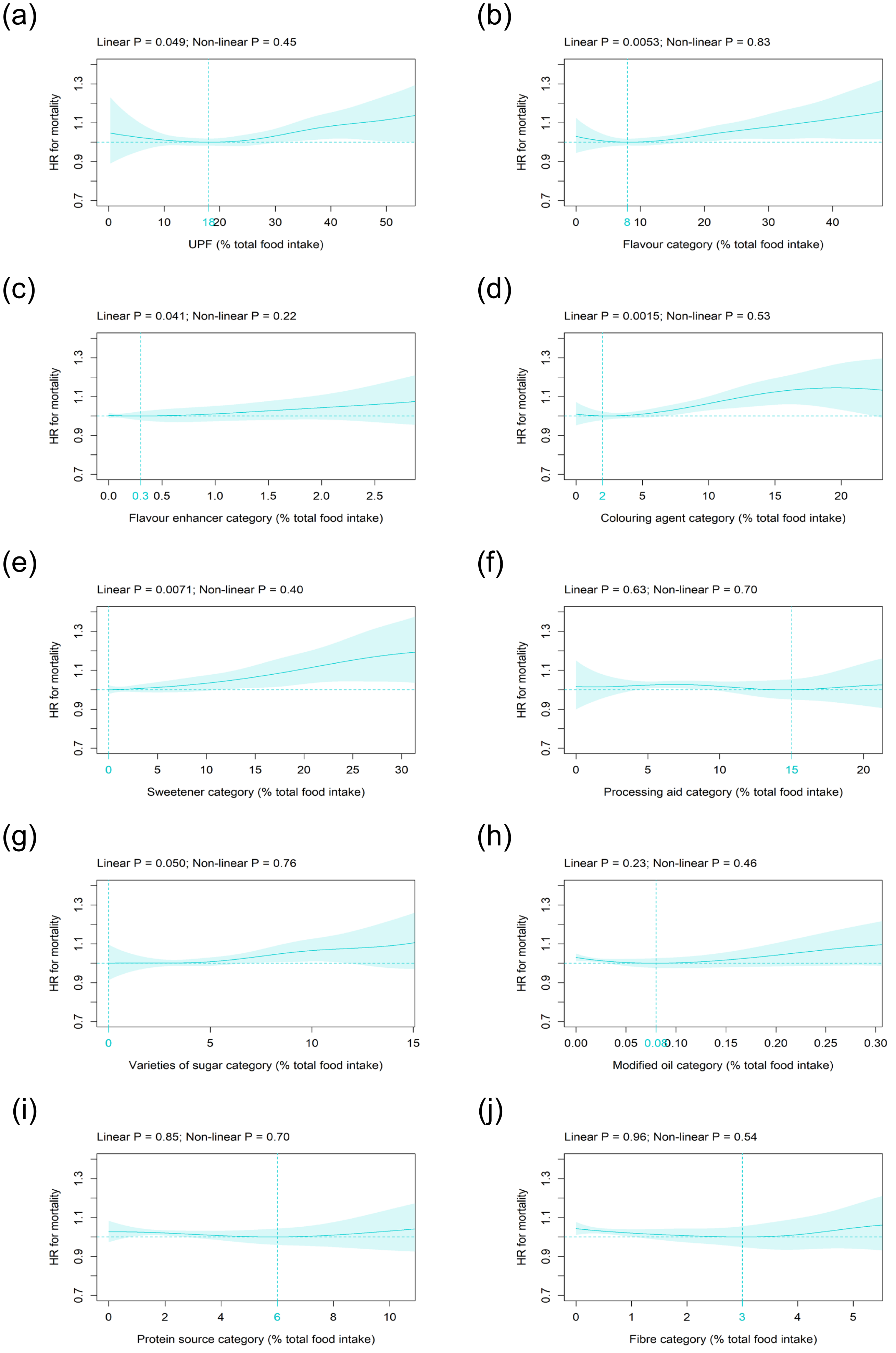

Fig. S10 - First Oxford WebQ only

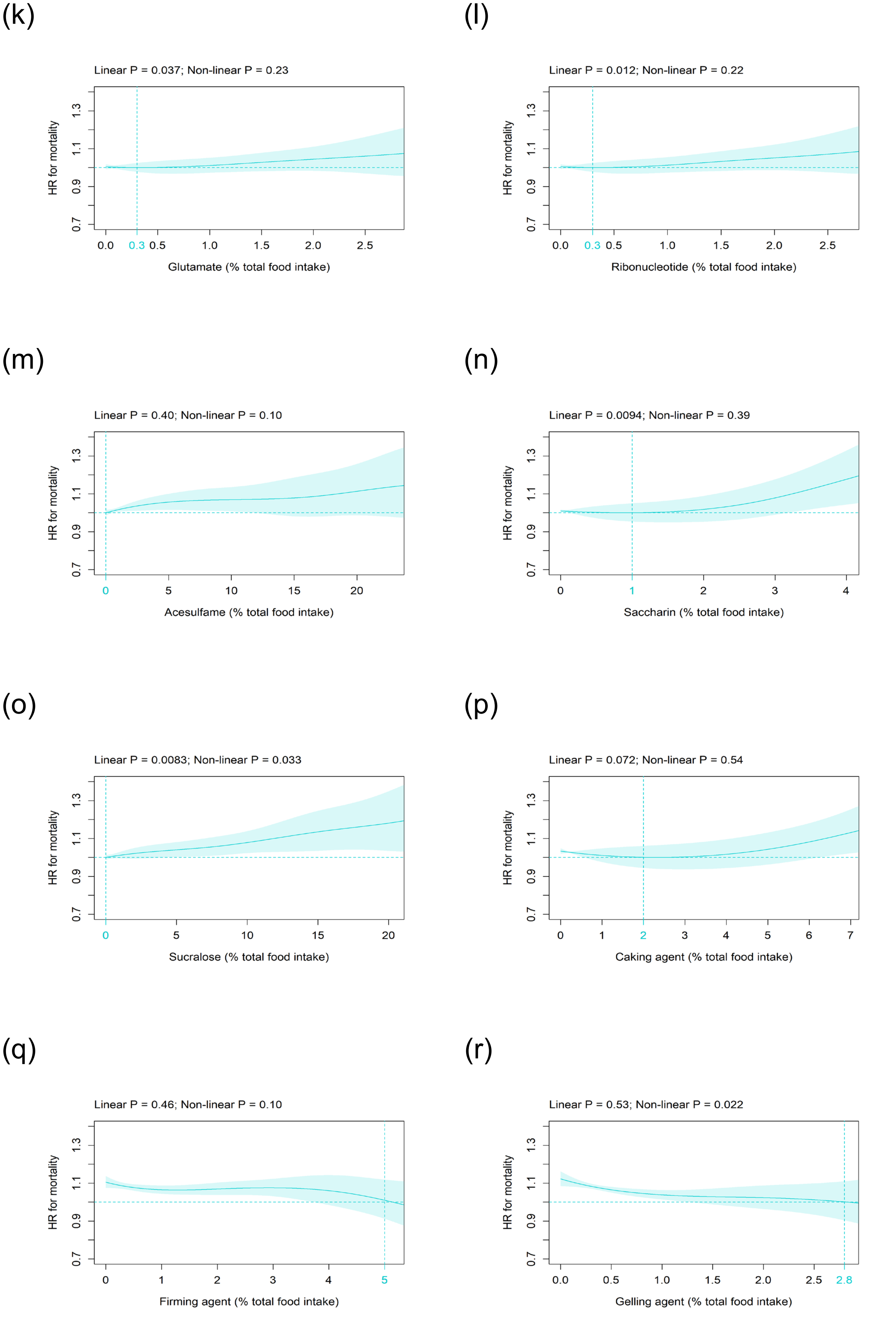

Fig. S10 - First Oxford WebQ only

(s)

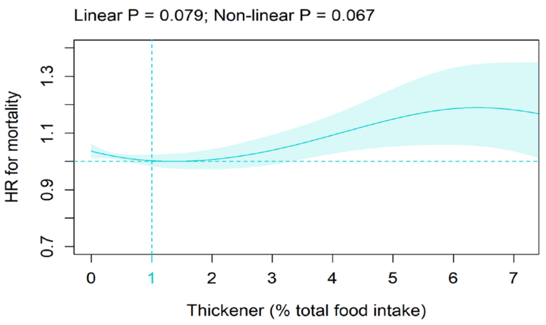

(t)

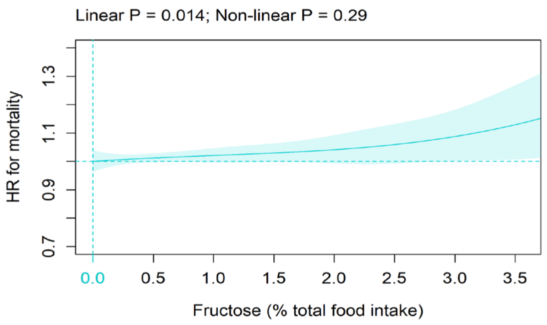

(u)

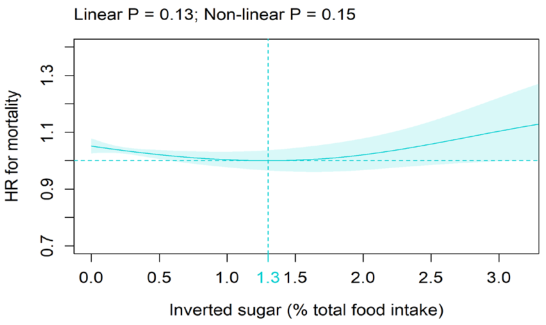

(v)

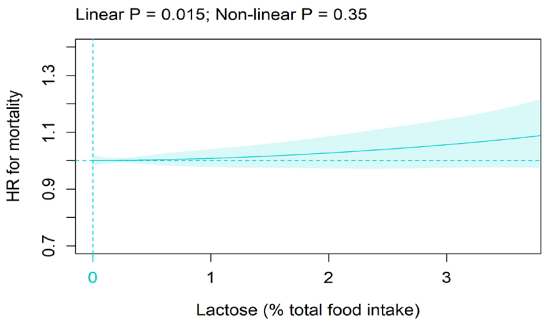

(w)

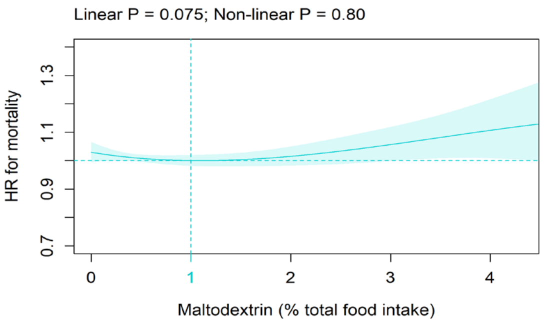

Fig. S10 - First Oxford WebQ only

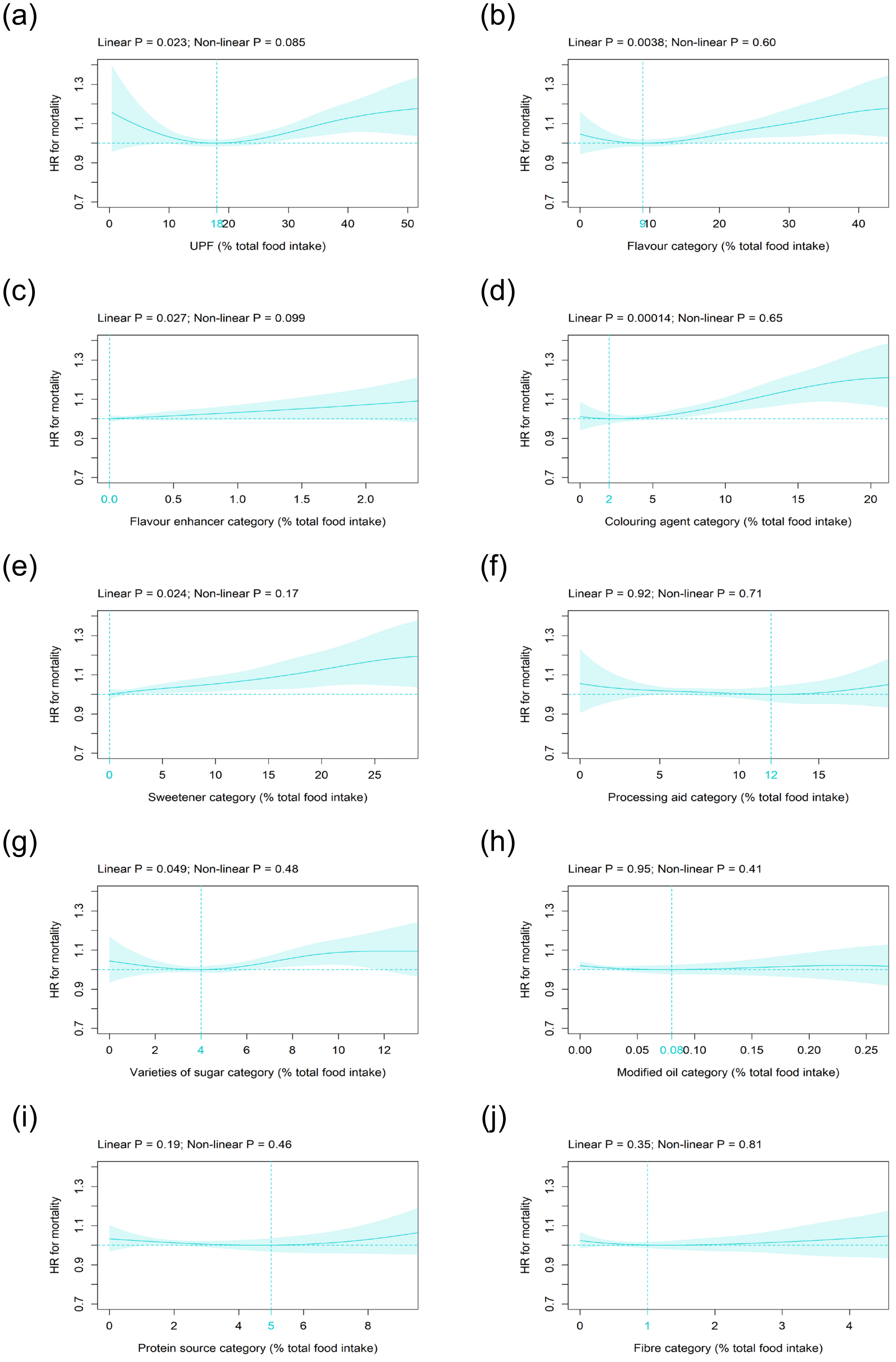

Fig. S11 - Further adjusted for diet quality

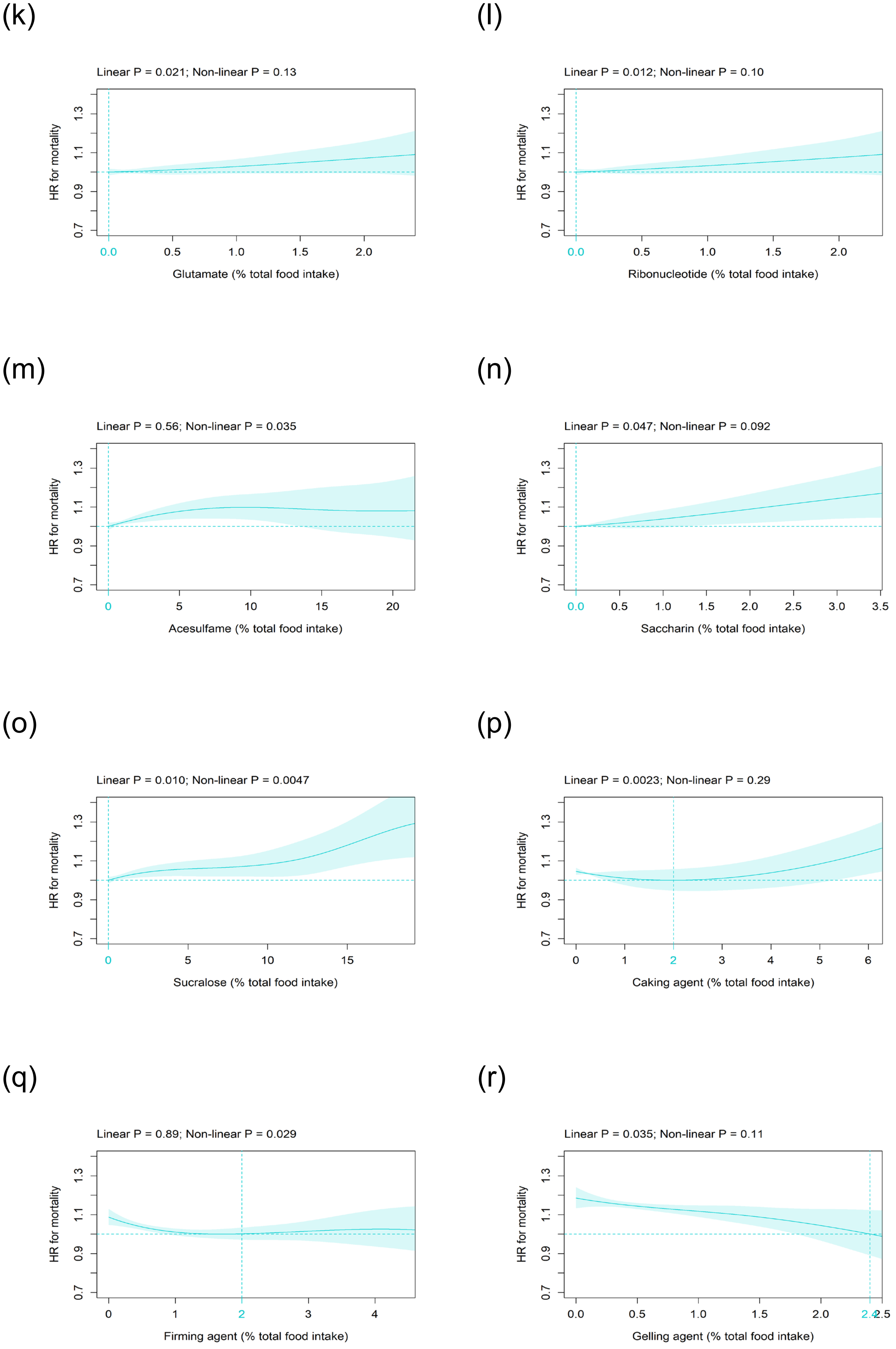

Fig. S11 - Further adjusted for diet quality

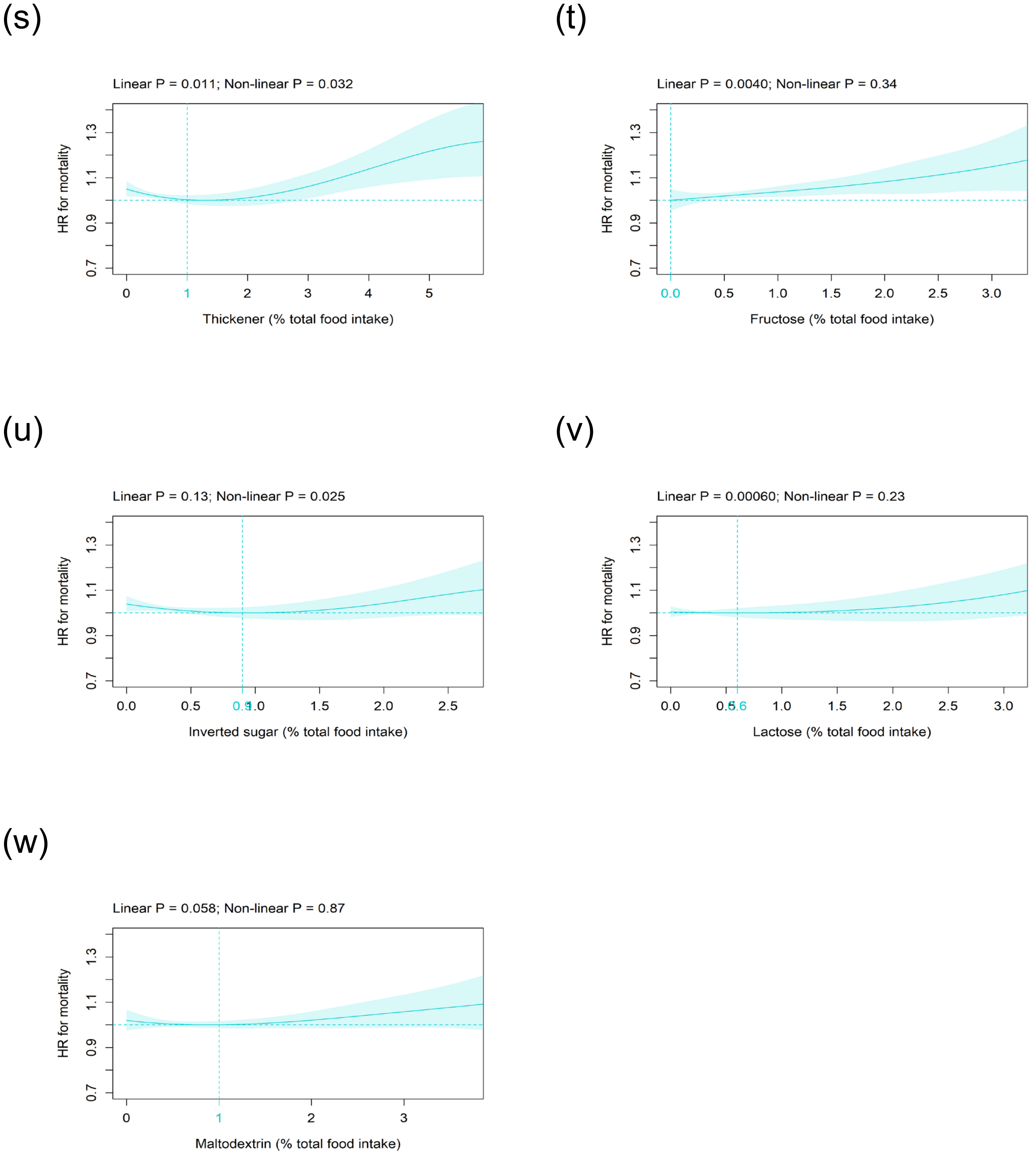

Fig. S11 - Further adjusted for diet quality

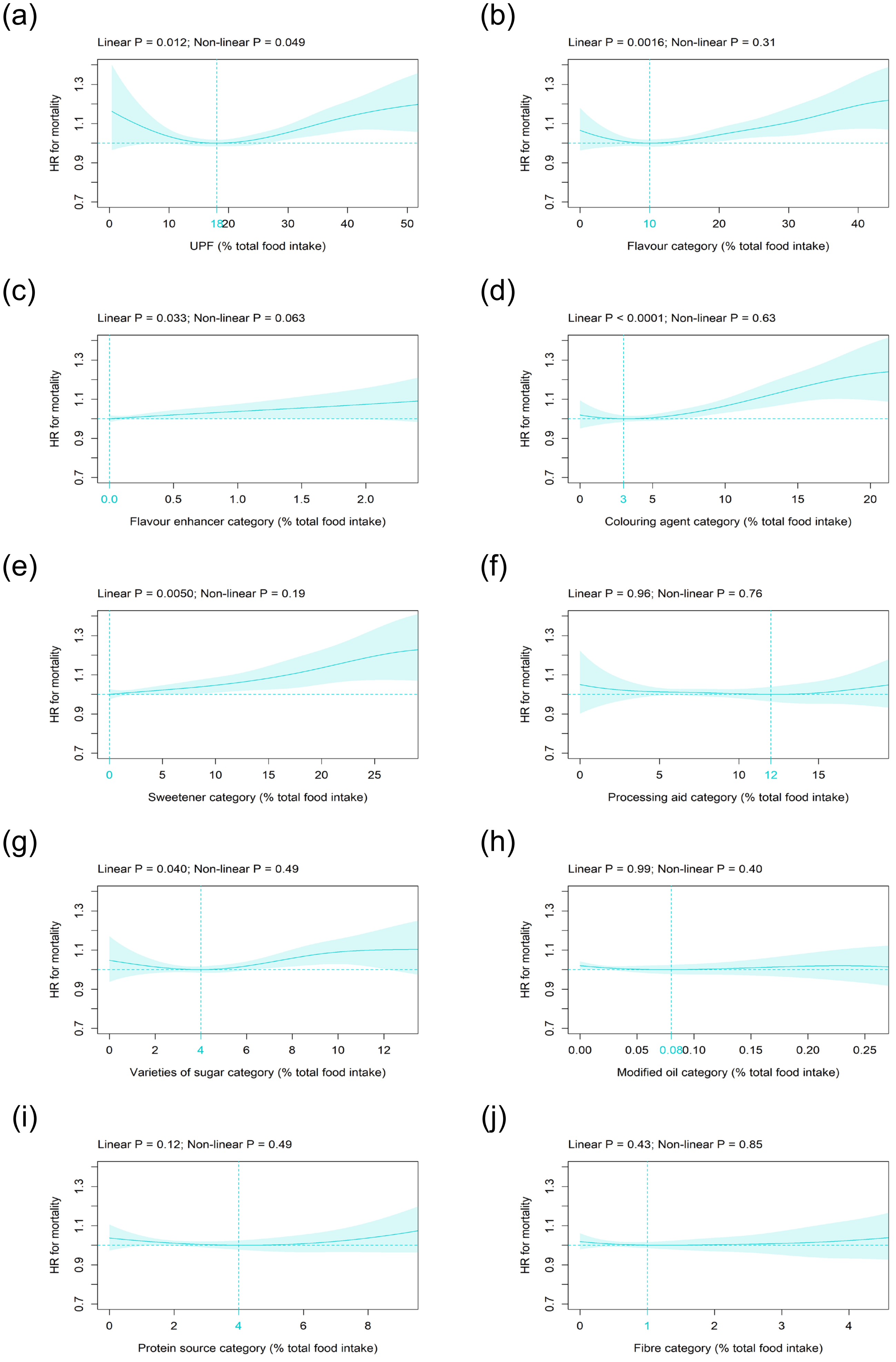

Fig. S12 - WHR and height instead of BMI

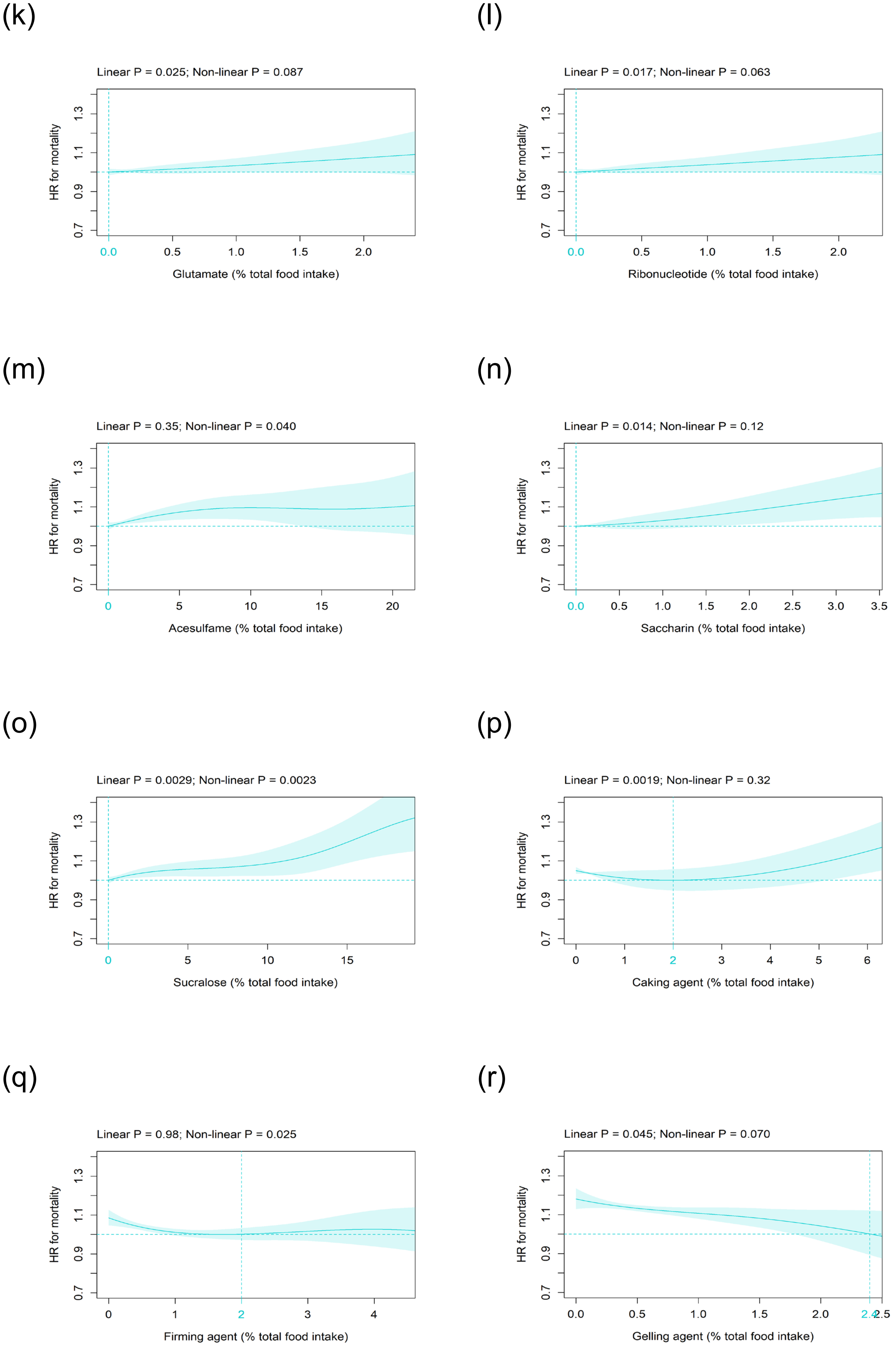

Fig. S12 - WHR and height instead of BMI

(s)

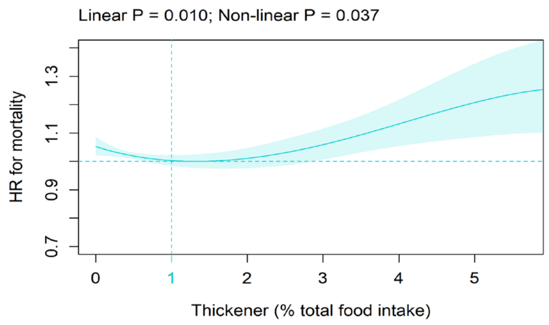

(t)

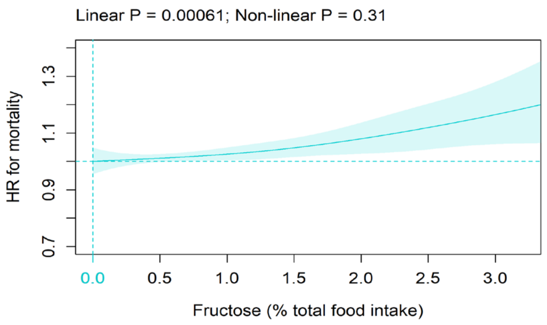

(u)

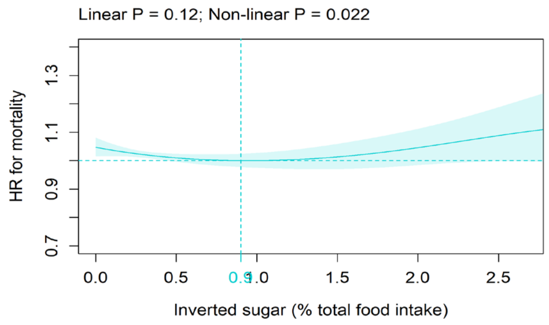

(v)

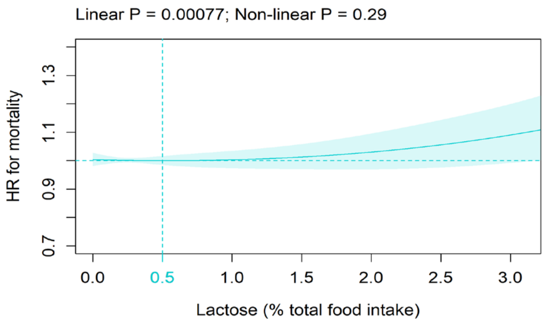

(w)

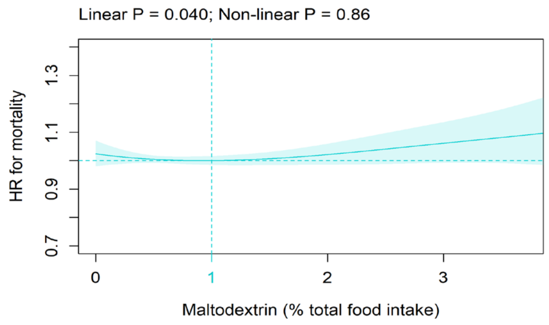

Fig. S12 - WHR and height instead of BMI

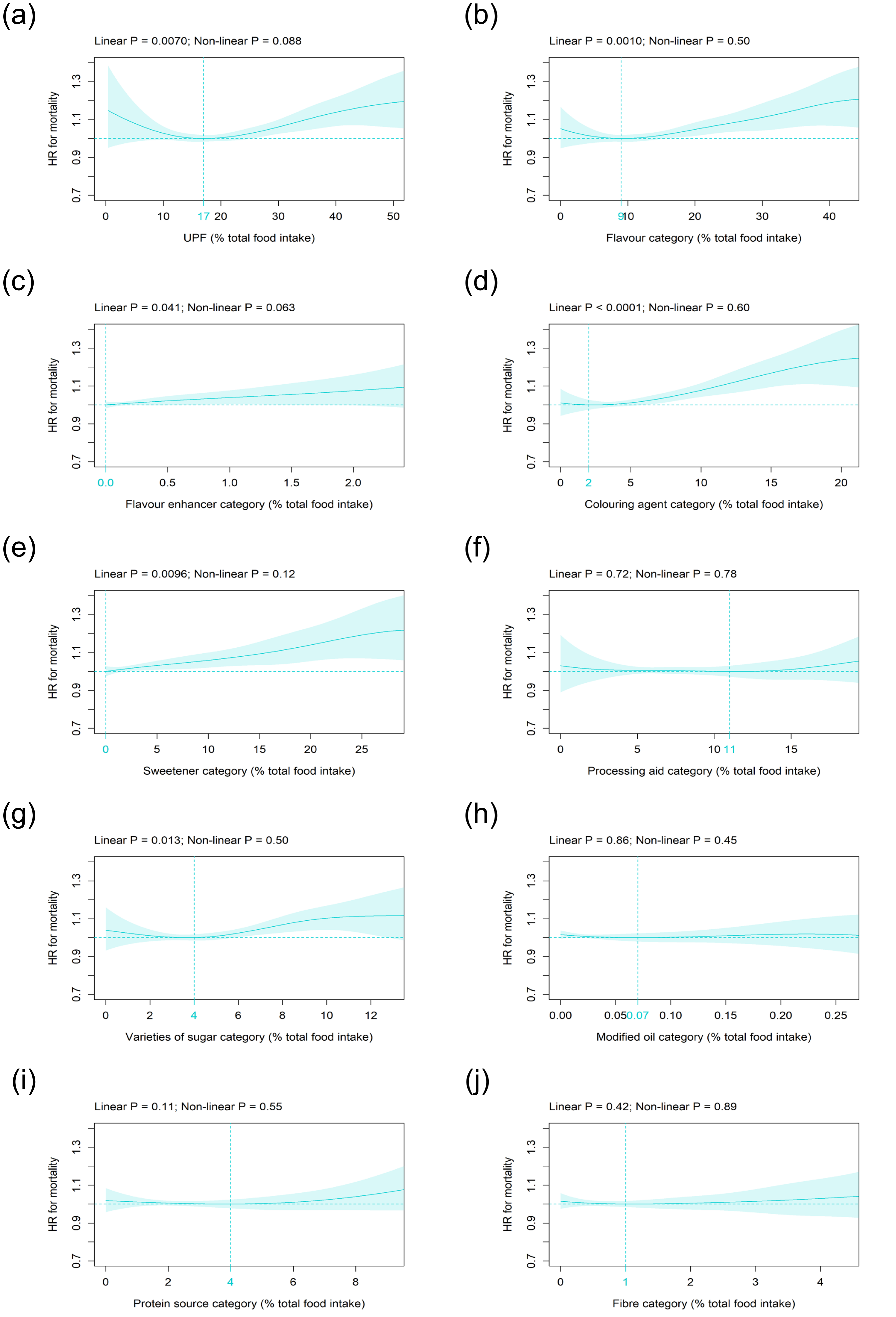

Fig. S13 - Energy intake removed

(k)

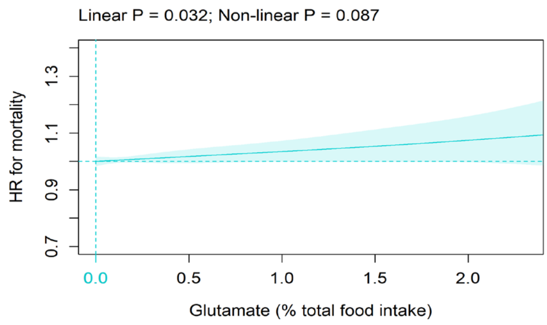

(l)

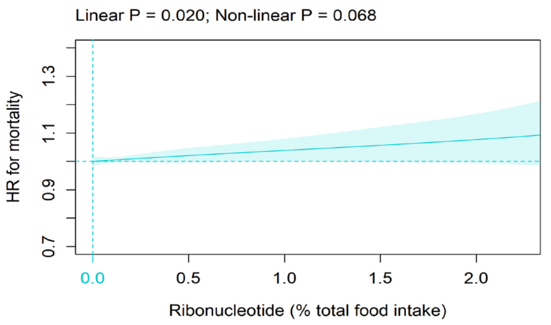

(m)

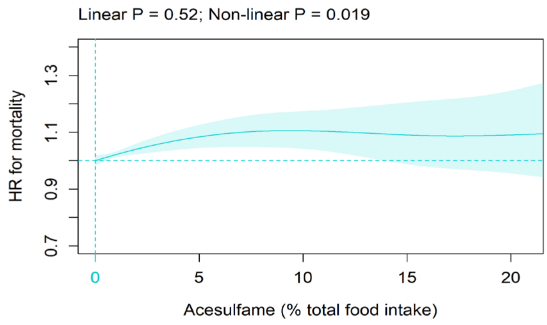

(n)

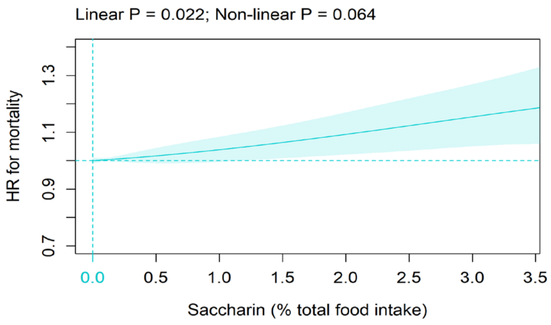

(o)

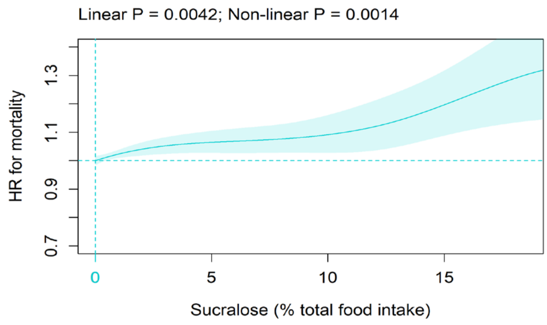

(p)

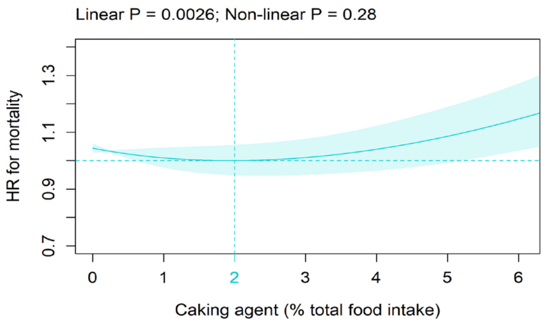

(q)

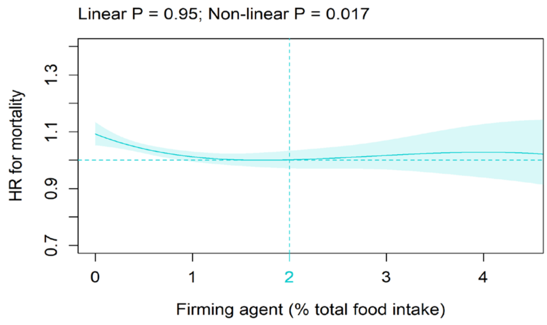

(r)

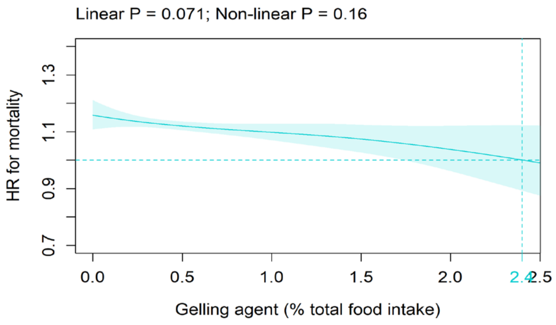

Fig. S13 - Energy intake removed

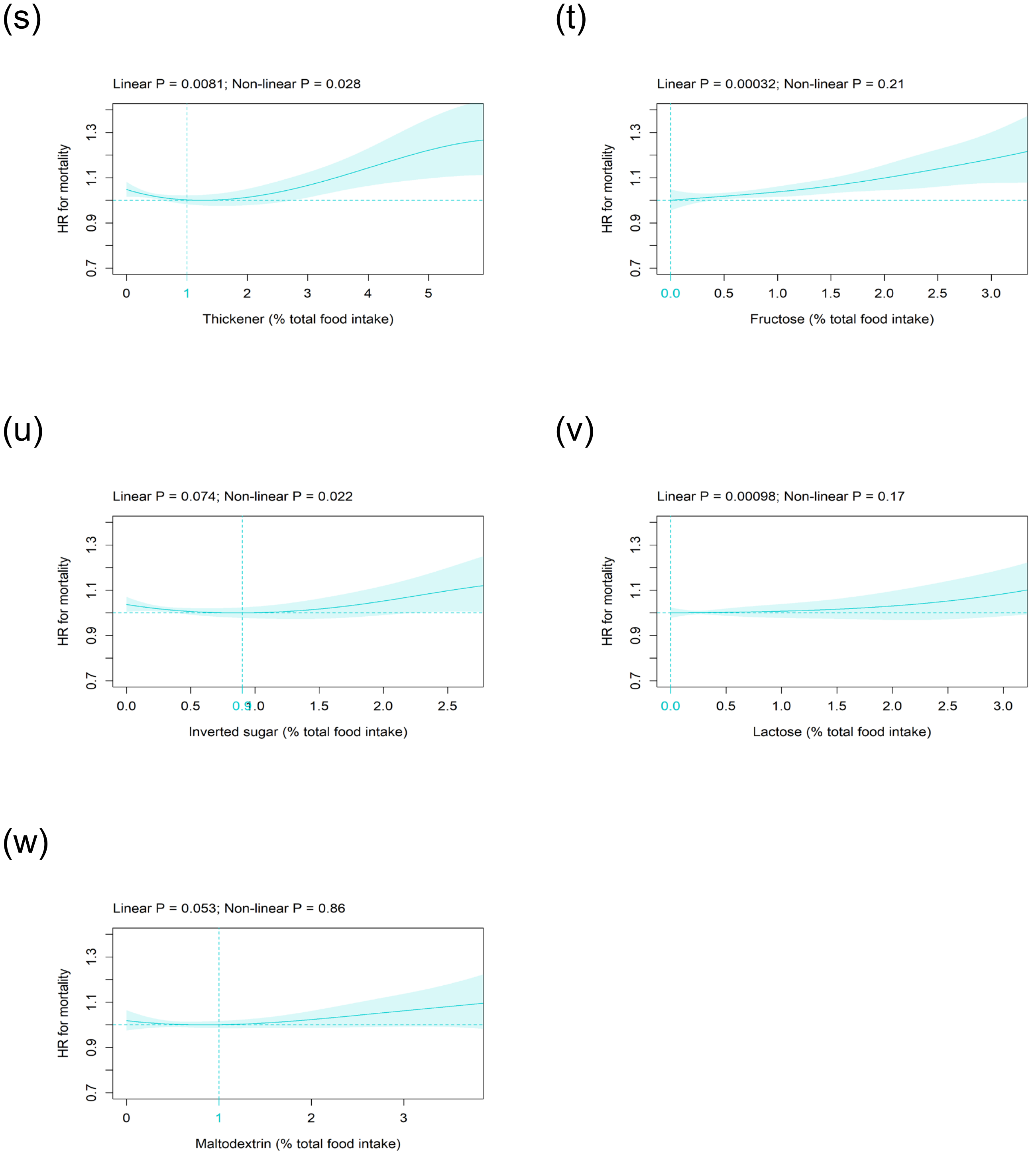

Fig. S13 - Energy intake removed

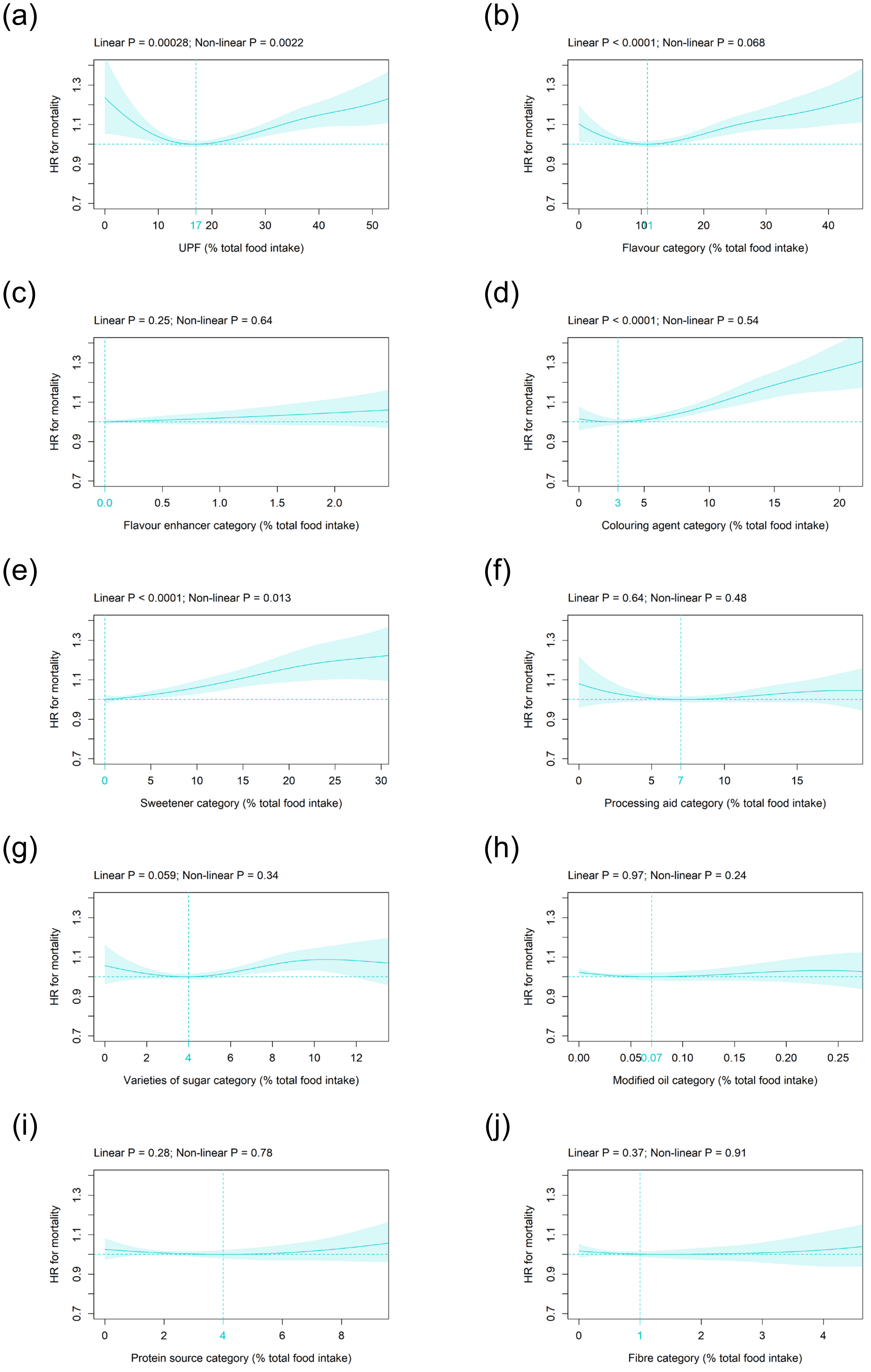

Fig. S14 - Imputation k nearest neighbour

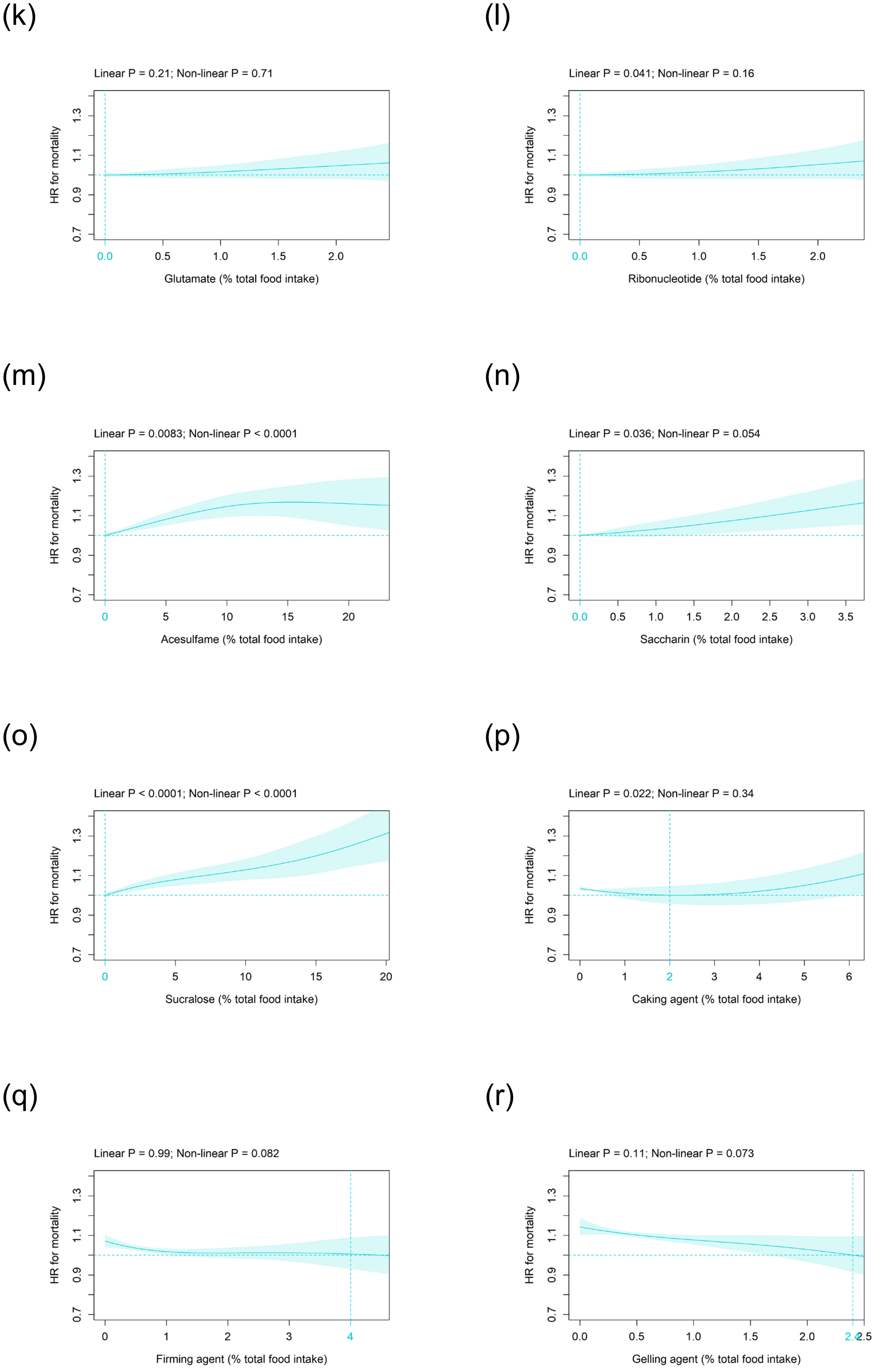

Fig. S14 - Imputation k nearest neighbour

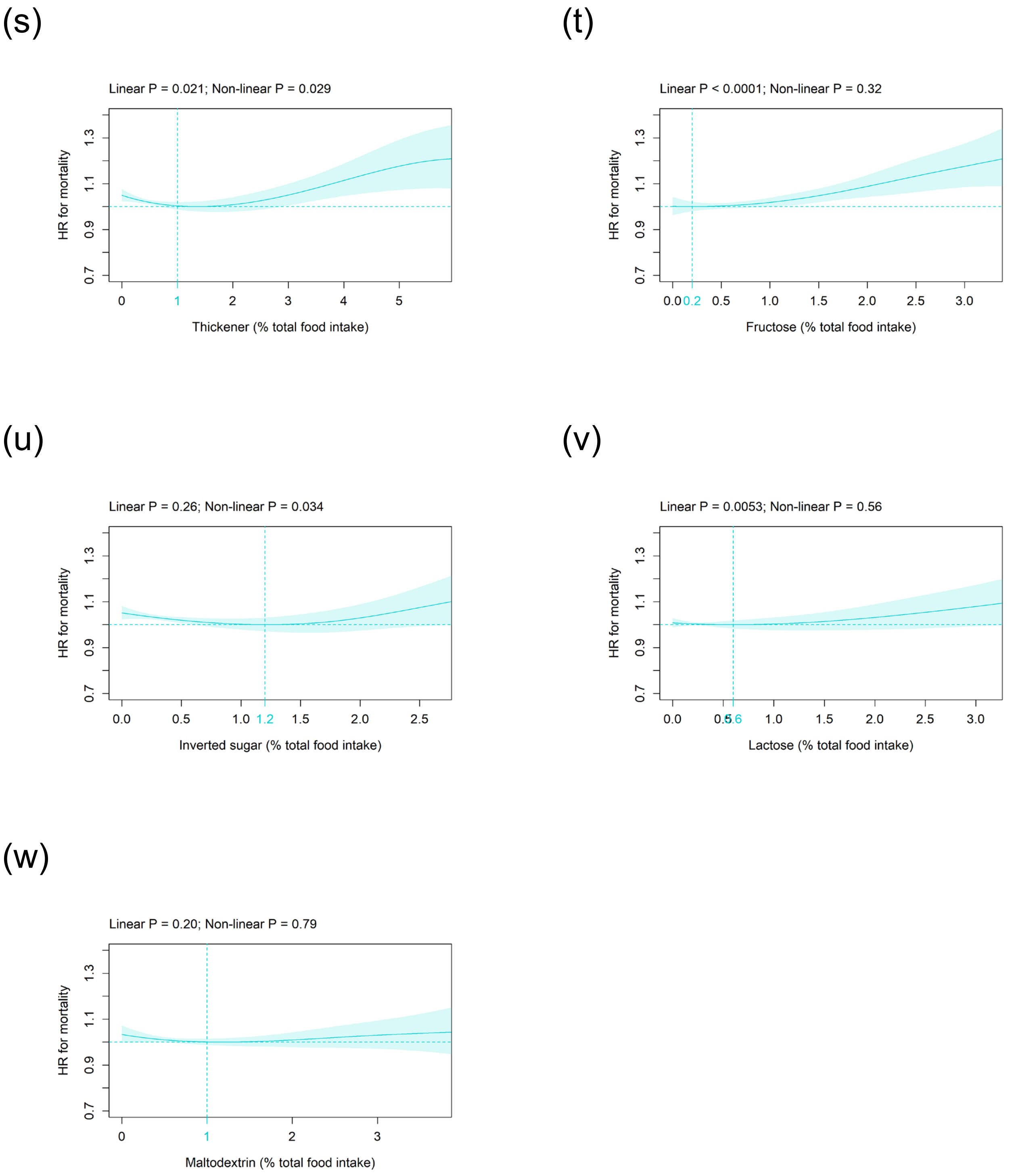

Fig. S14 - Imputation k nearest neighbour

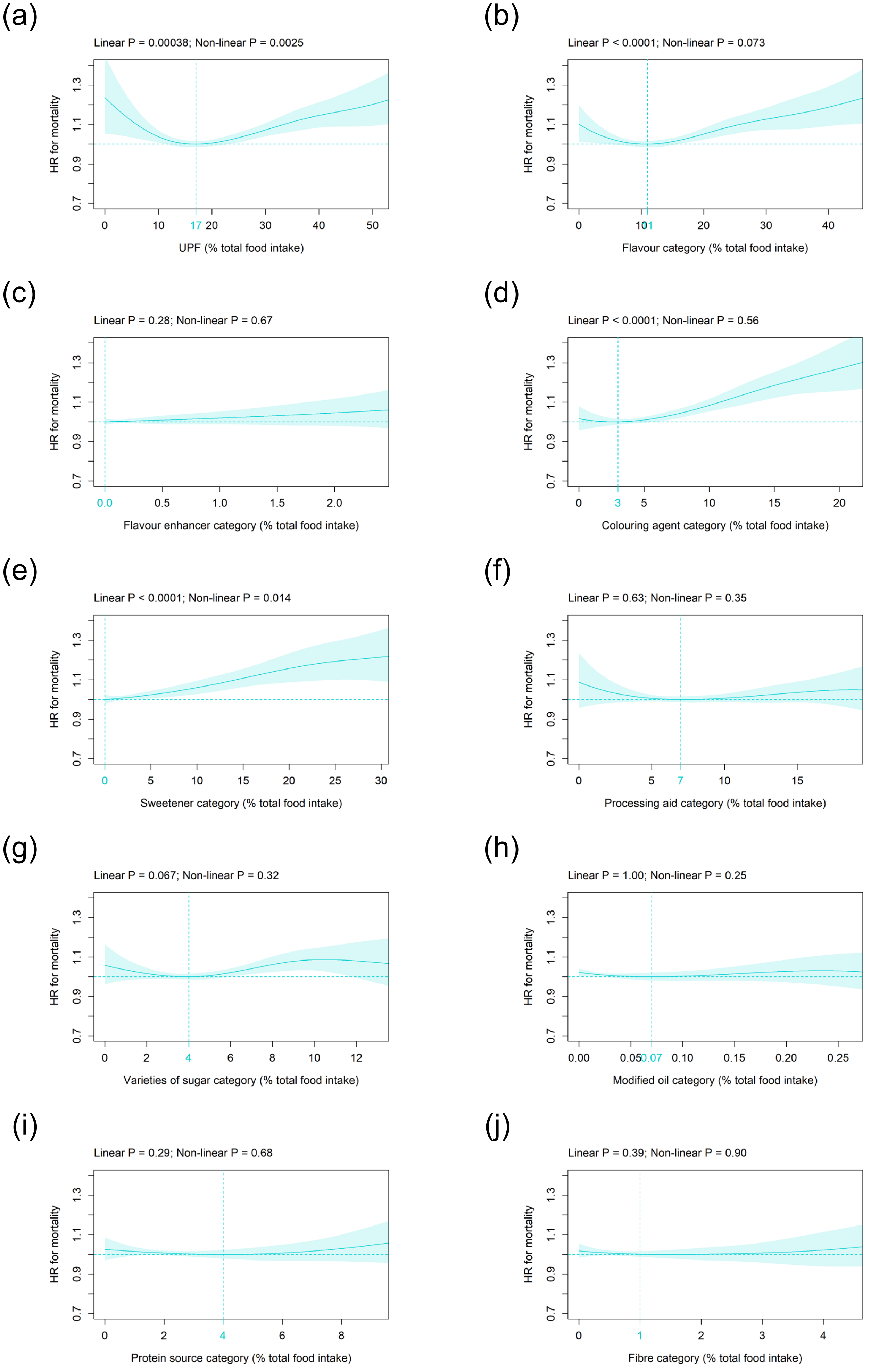

Fig. S15 - Imputation random forrest

(k)

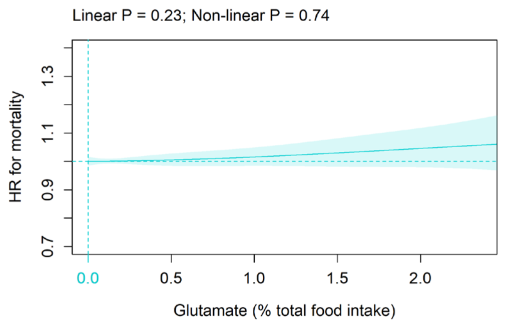

(l)

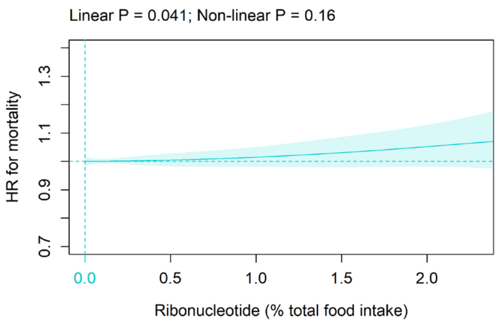

(m)

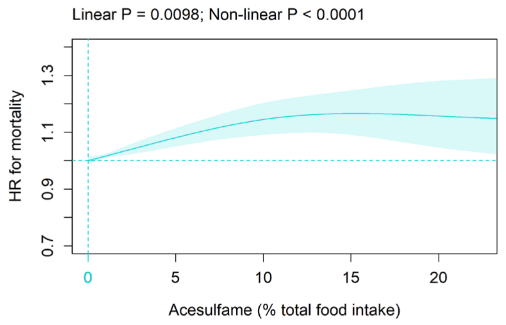

(n)

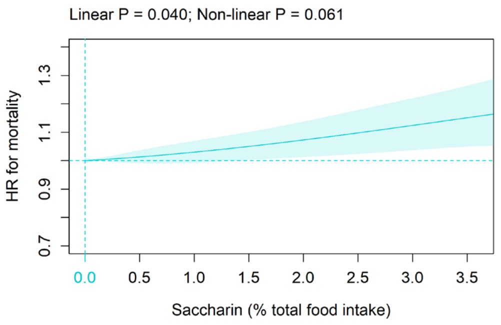

(o)

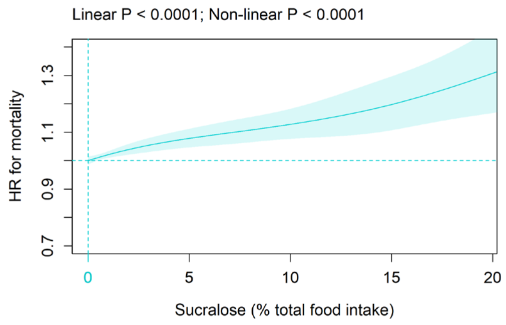

(p)

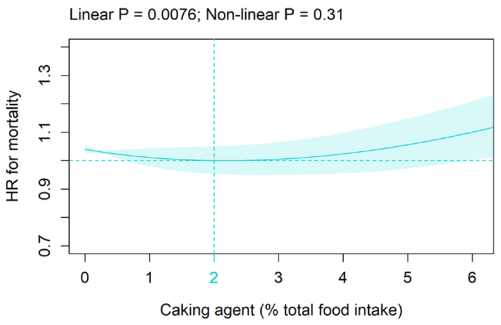

(q)

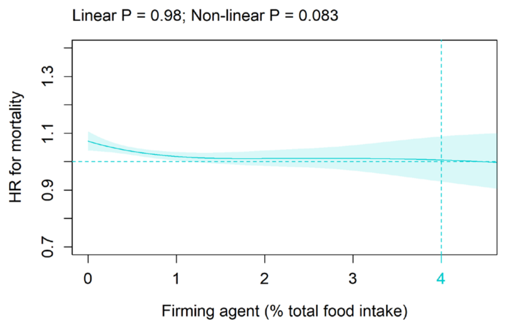

(r)

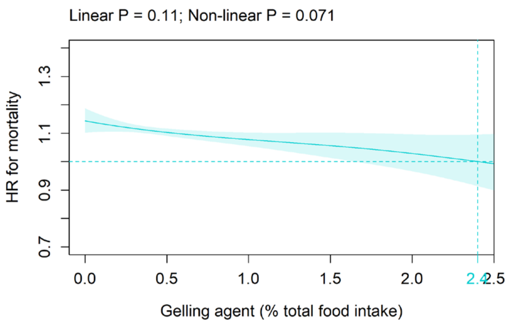

Fig. S15 - Imputation random forrest

(s)

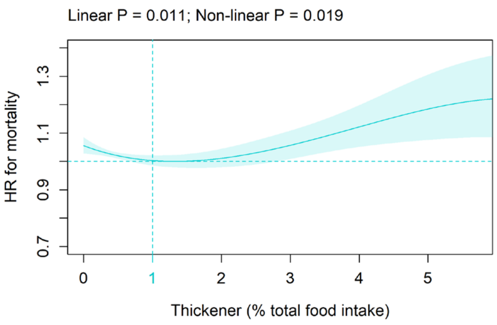

(t)

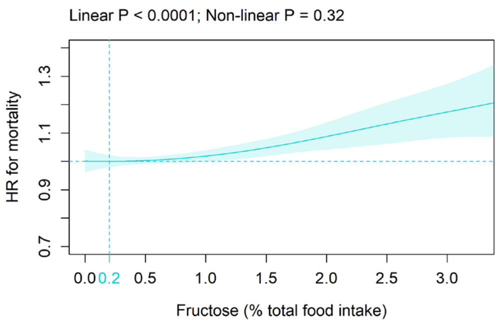

(u)

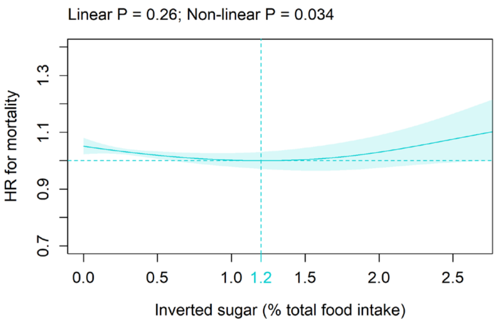

(v)

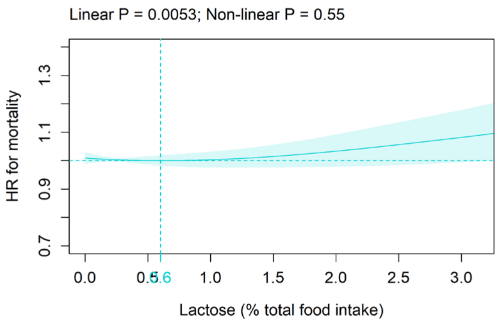

(w)

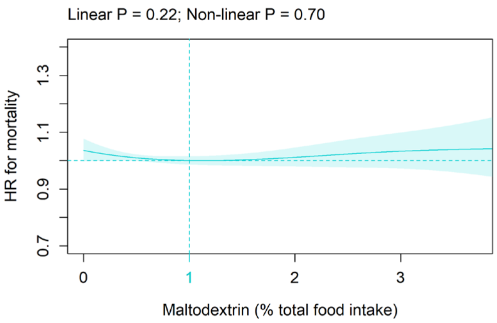

Fig. S15 - Imputation random forrest

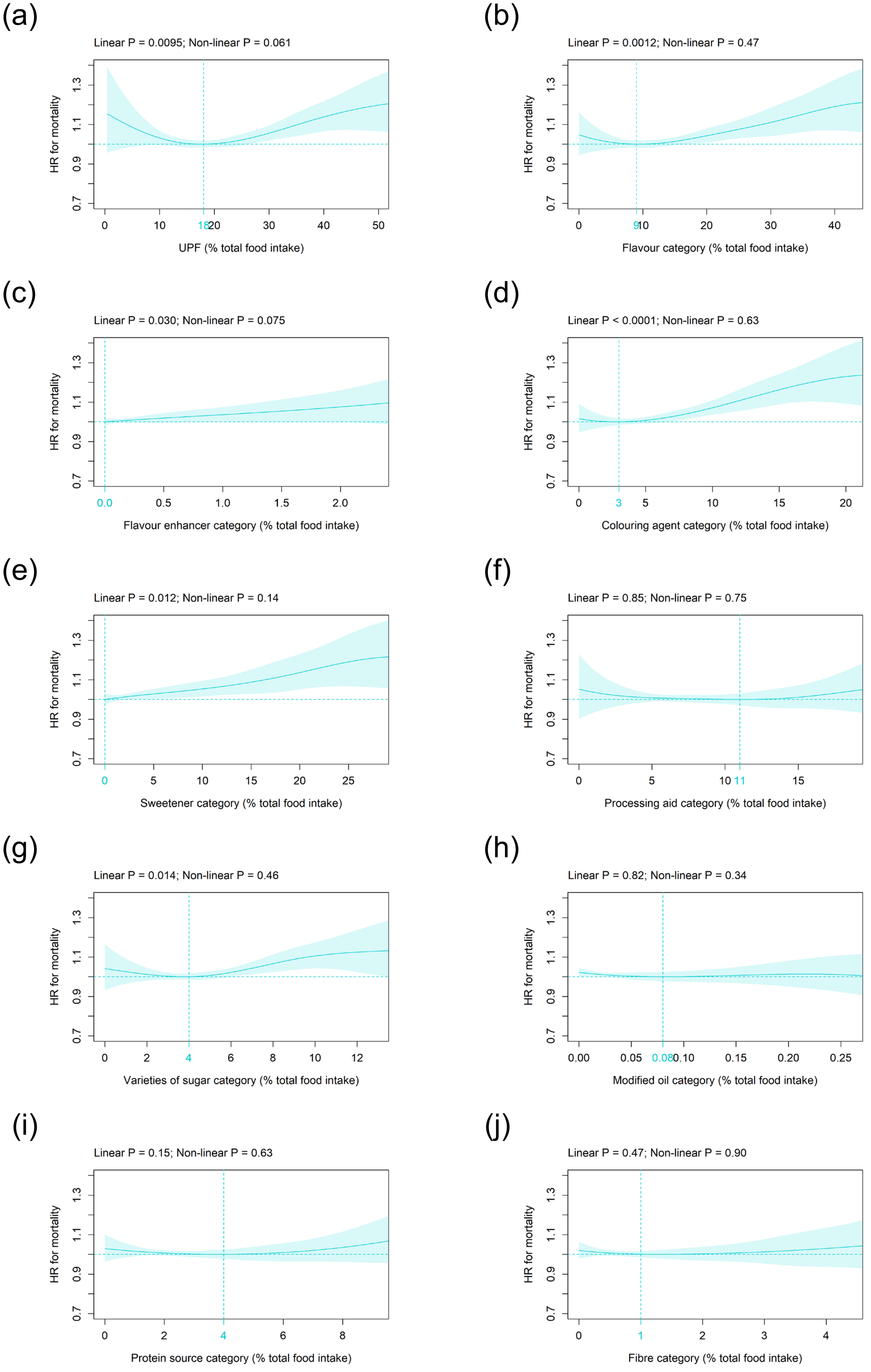

Fig. S16 - Further adjusted for cardiometabolic disease medication

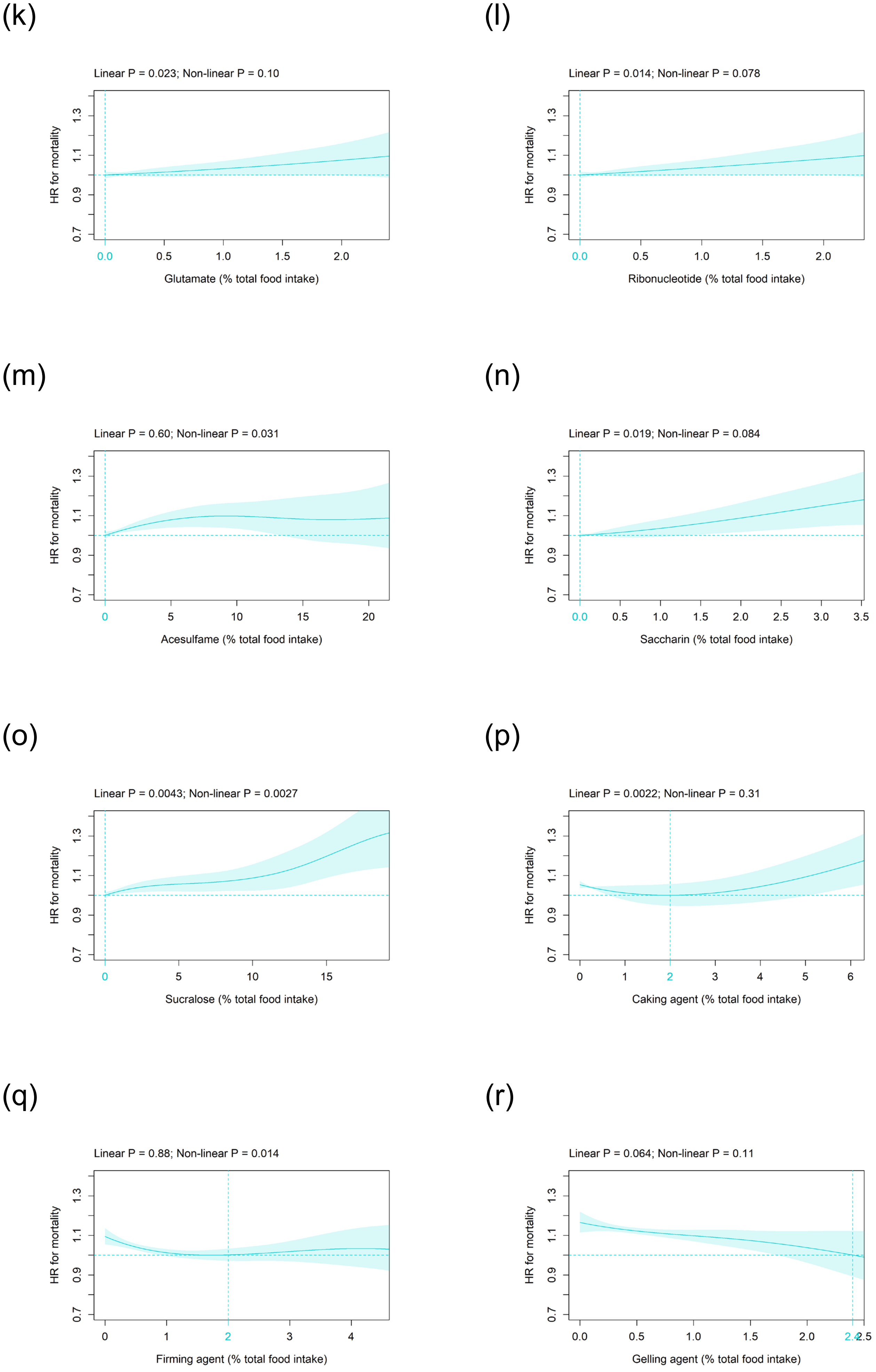

Fig. S16 - Further adjusted for cardiometabolic disease medication

(s)

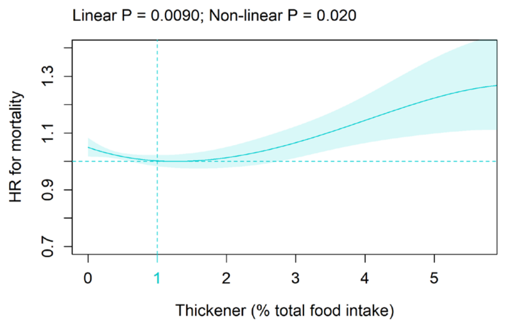

(t)

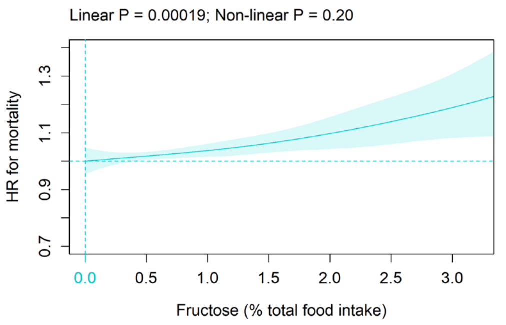

(u)

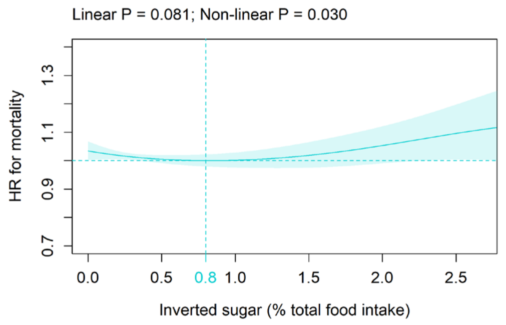

(v)

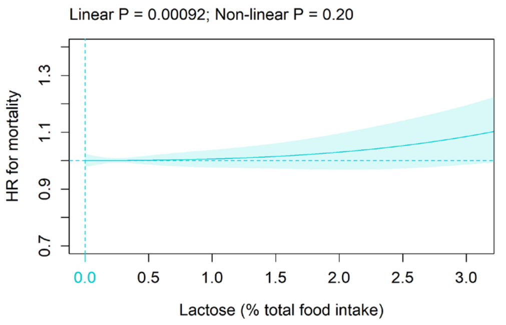

(w)

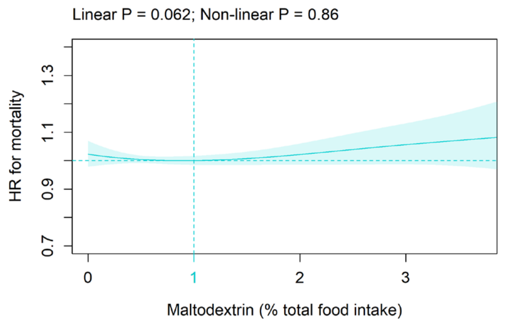

Fig. S16 - Further adjusted for cardiometabolic disease medication

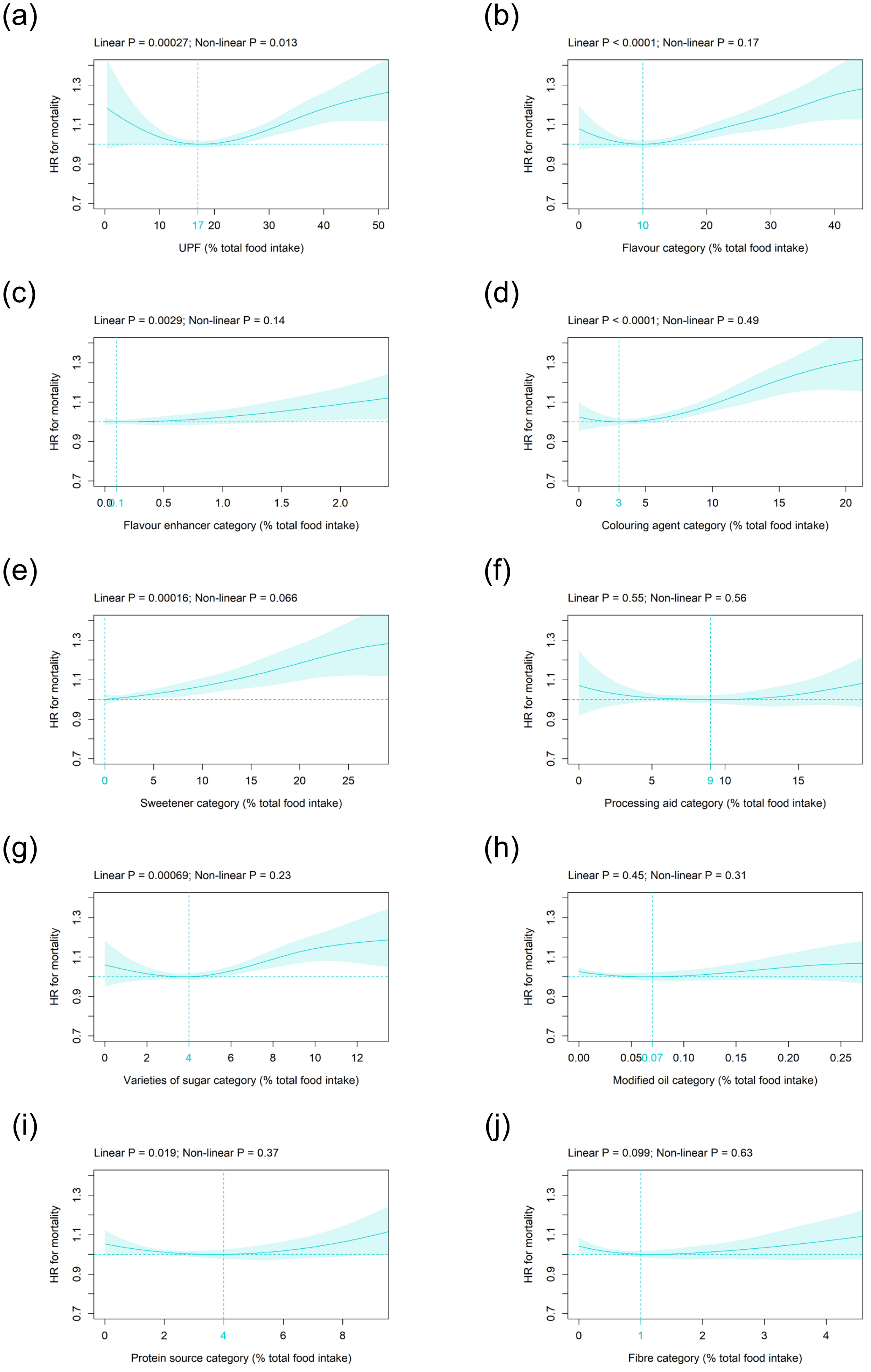

Fig. S17 - Include BMI and SBP as spline terms

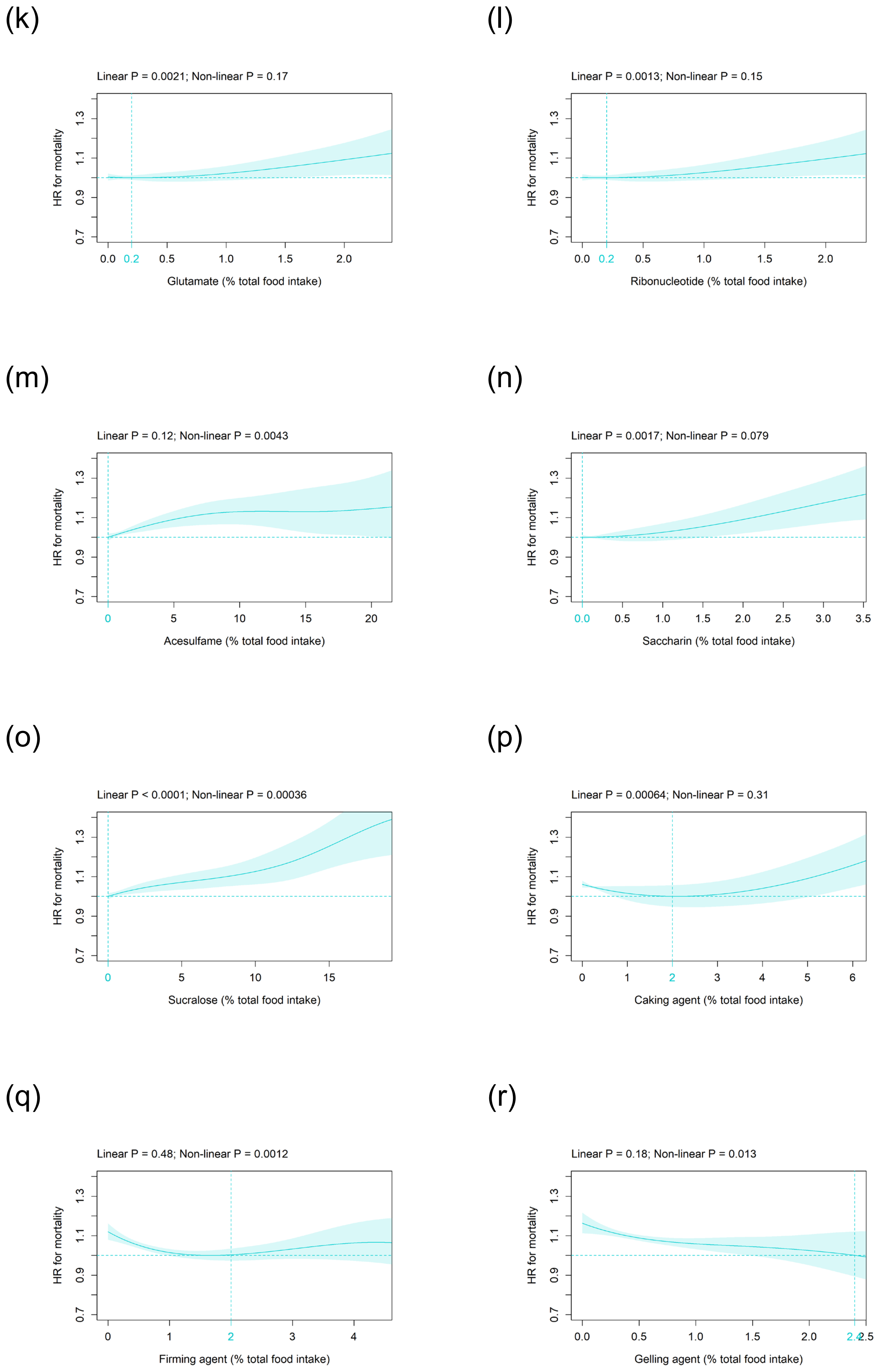

Fig. S17 - Include BMI and SBP as spline terms

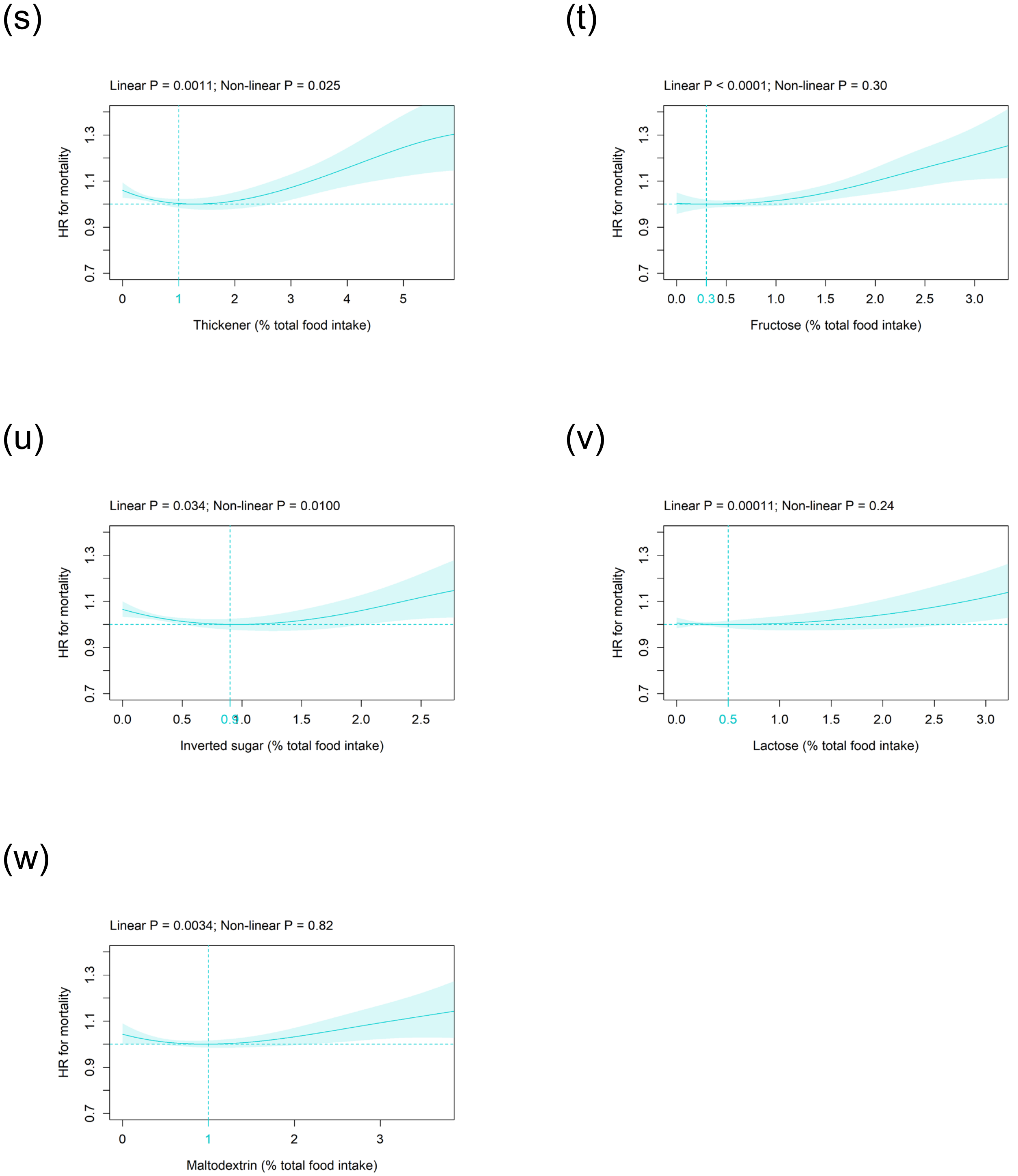

Fig. S17 - Include BMI and SBP as spline terms

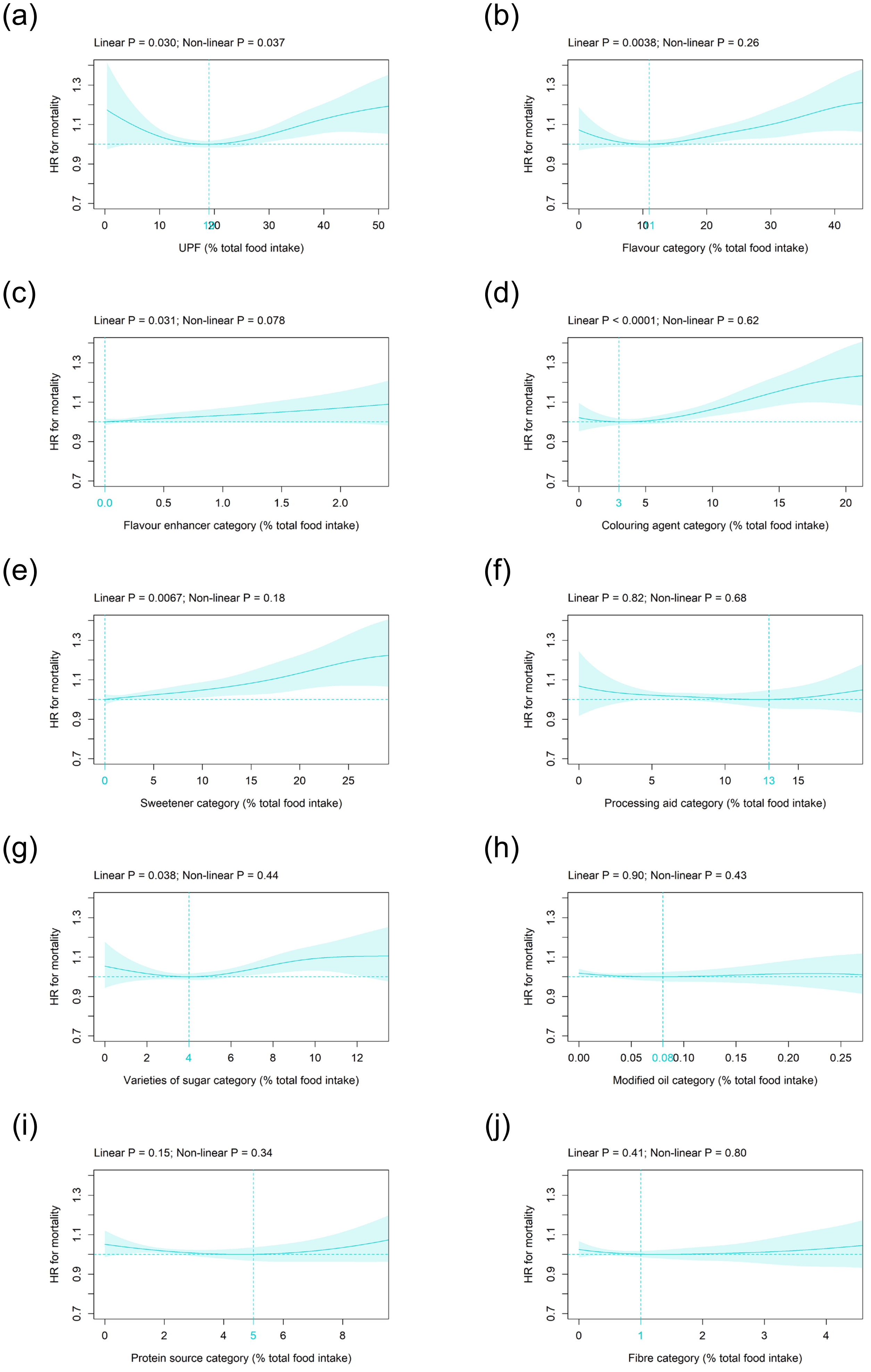

Fig. S18 - Further adjusted for assessment centre

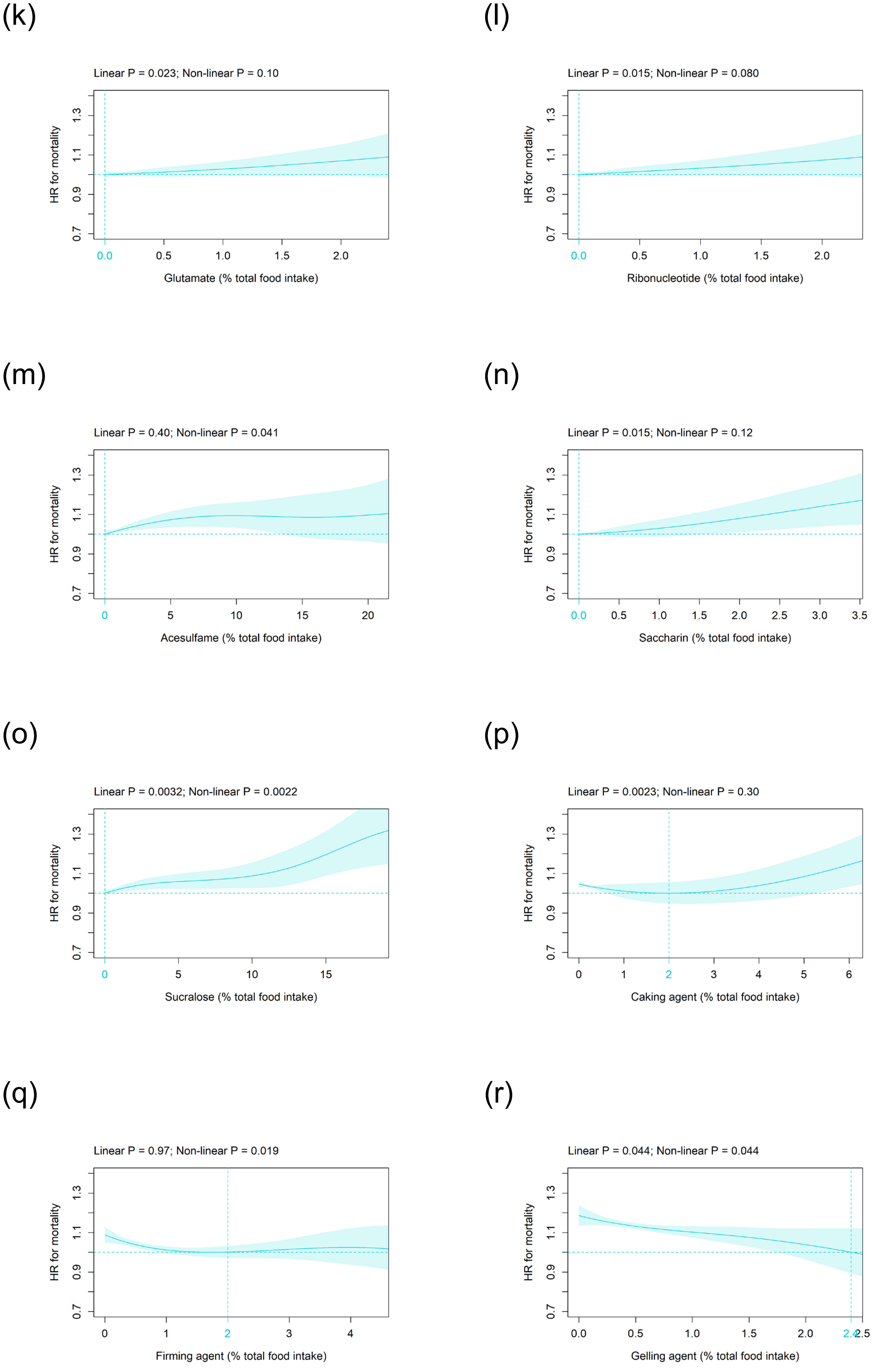

Fig. S18 - Further adjusted for assessment centre

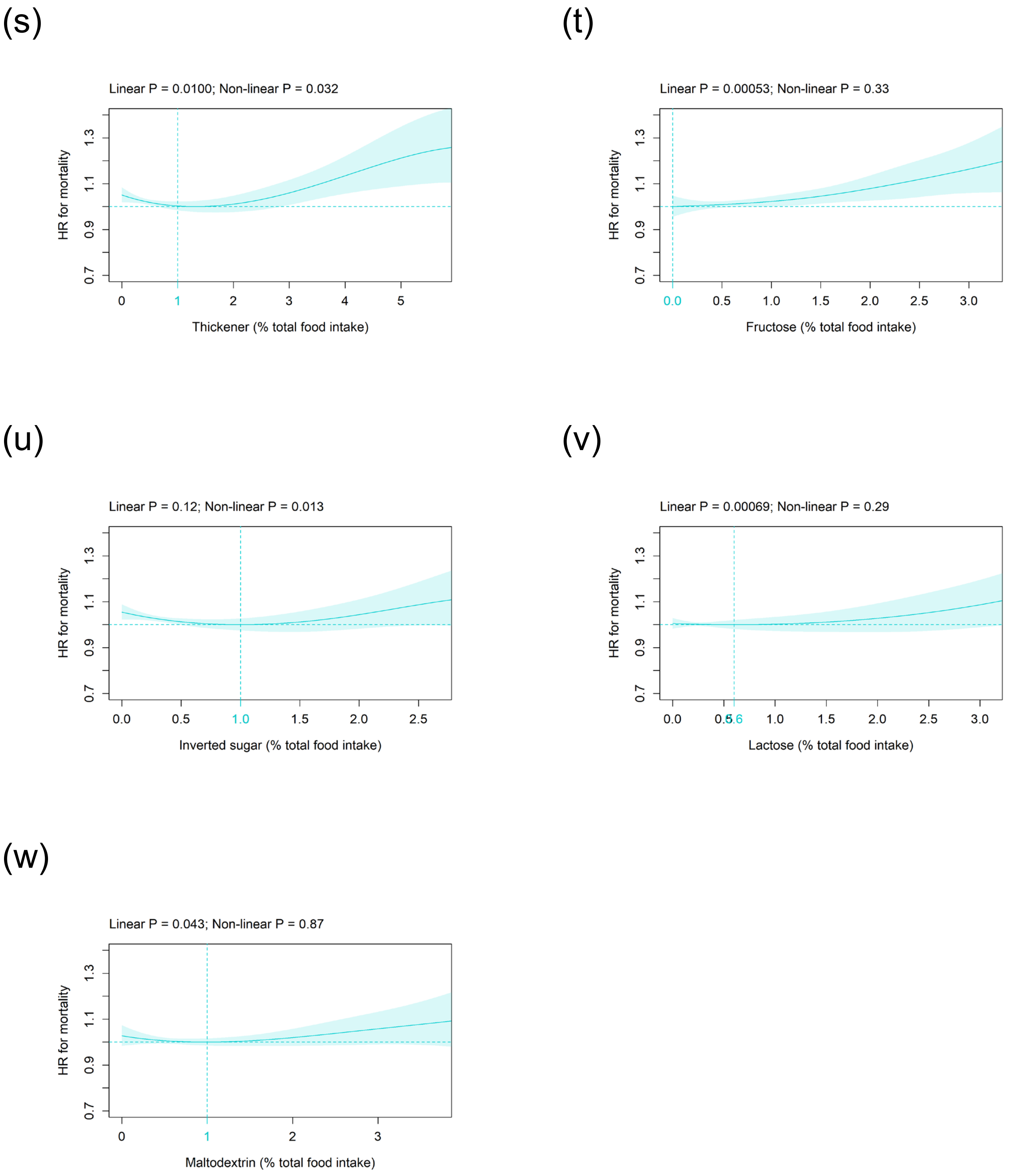

Fig. S18 - Further adjusted for assessment centre

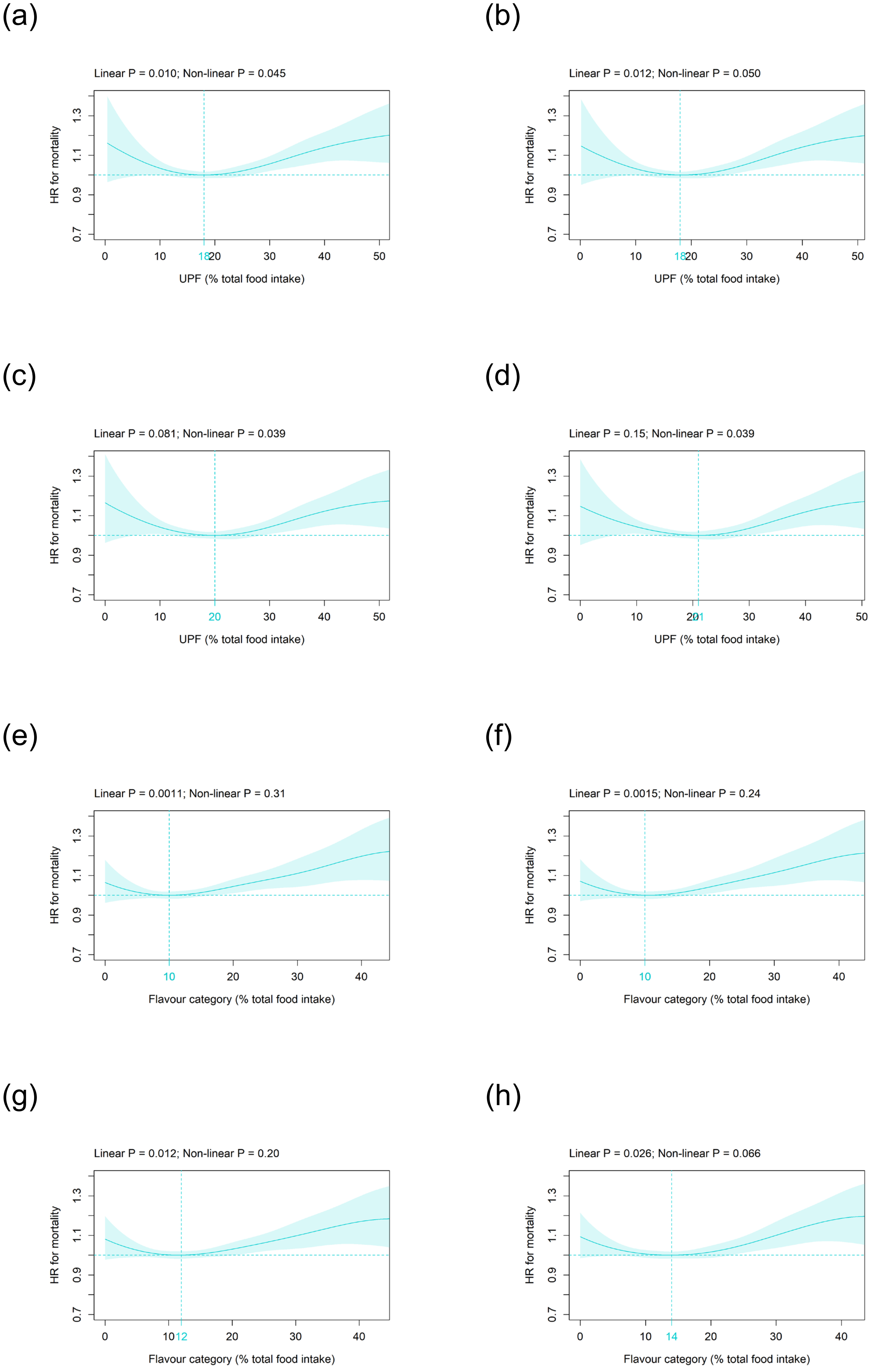

Fig. S19 - MLI with fewer food items

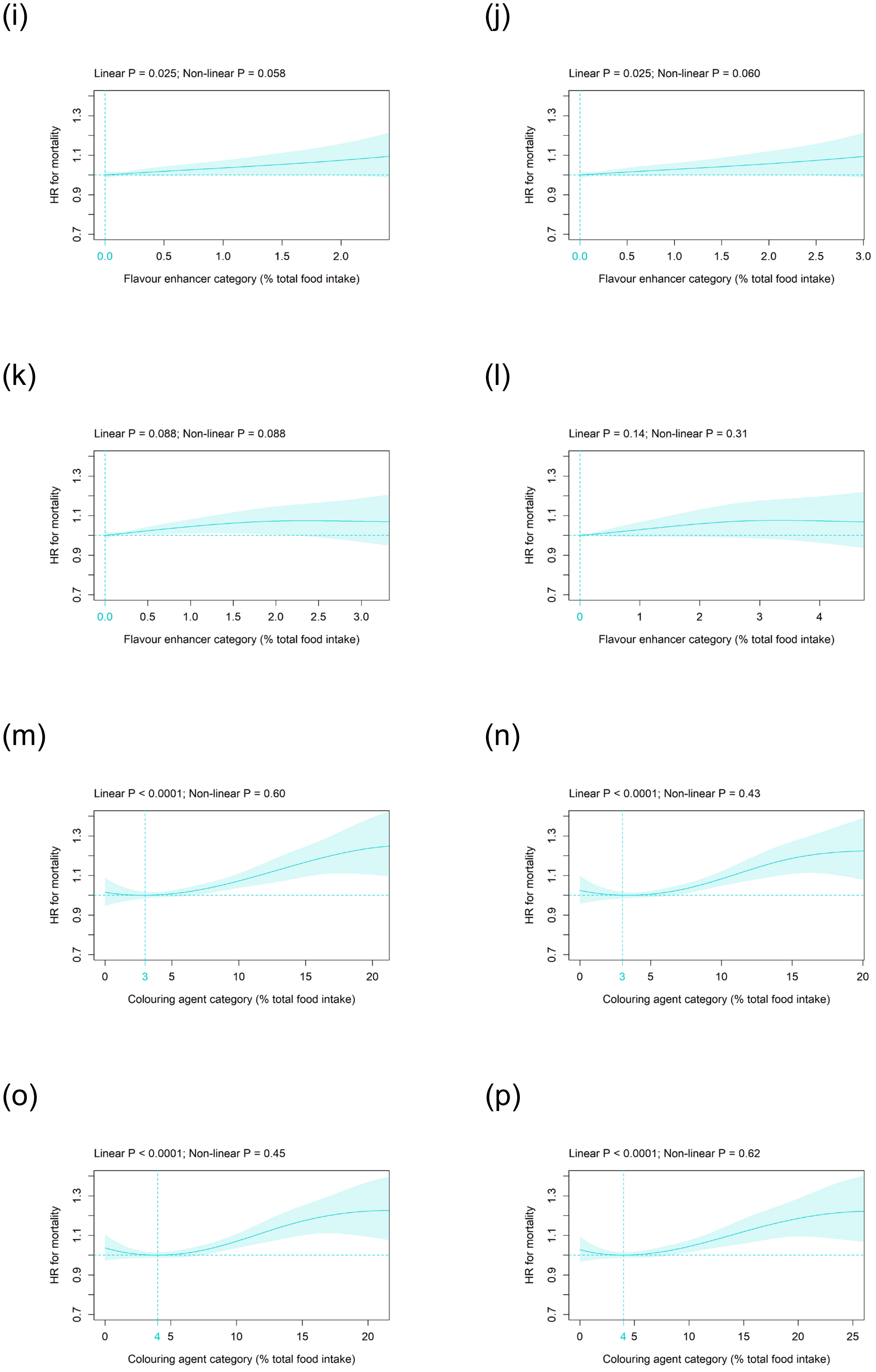

Fig. S19 - MLI with fewer food items

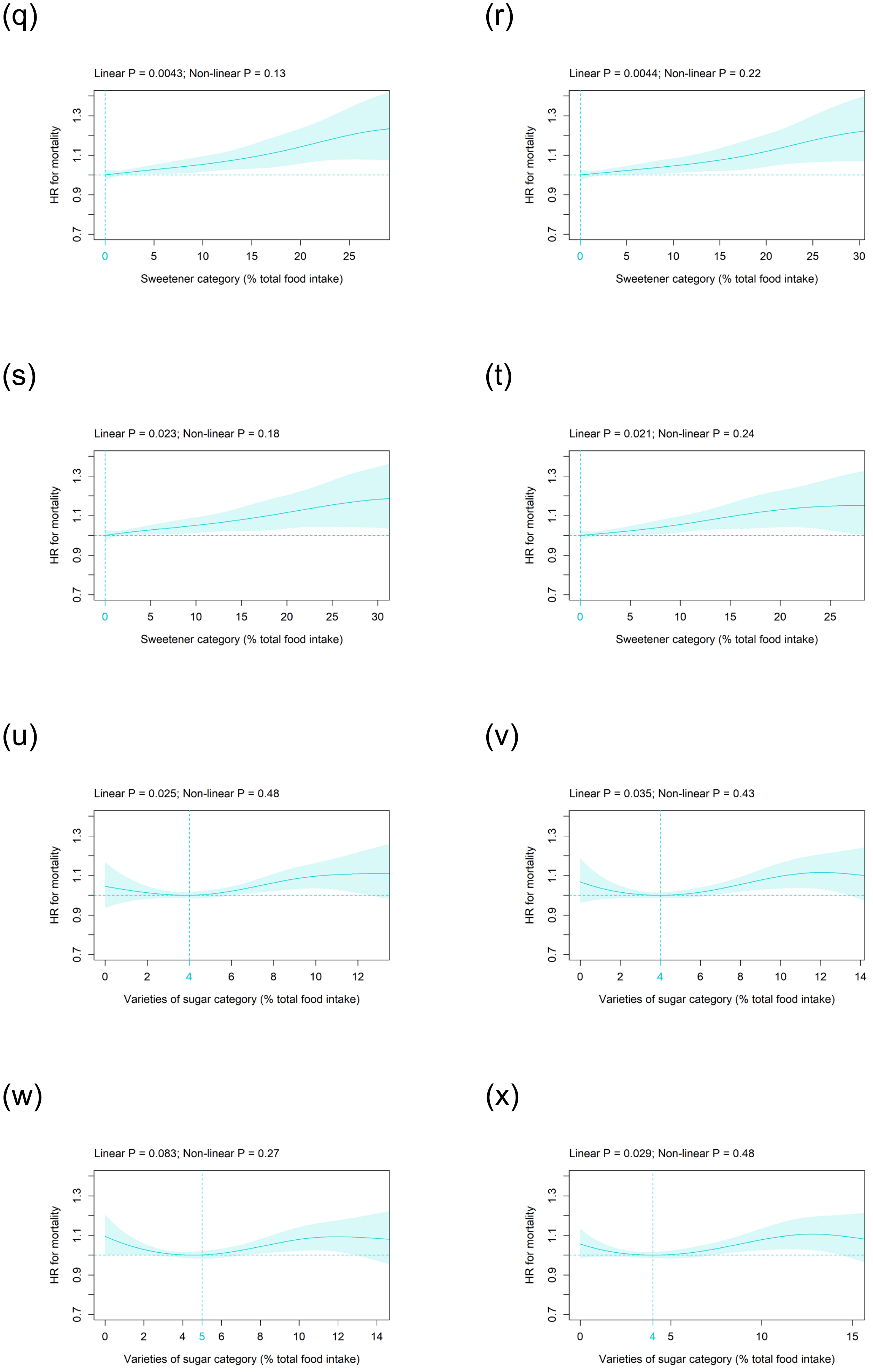

Fig. S19 - MLI with fewer food items

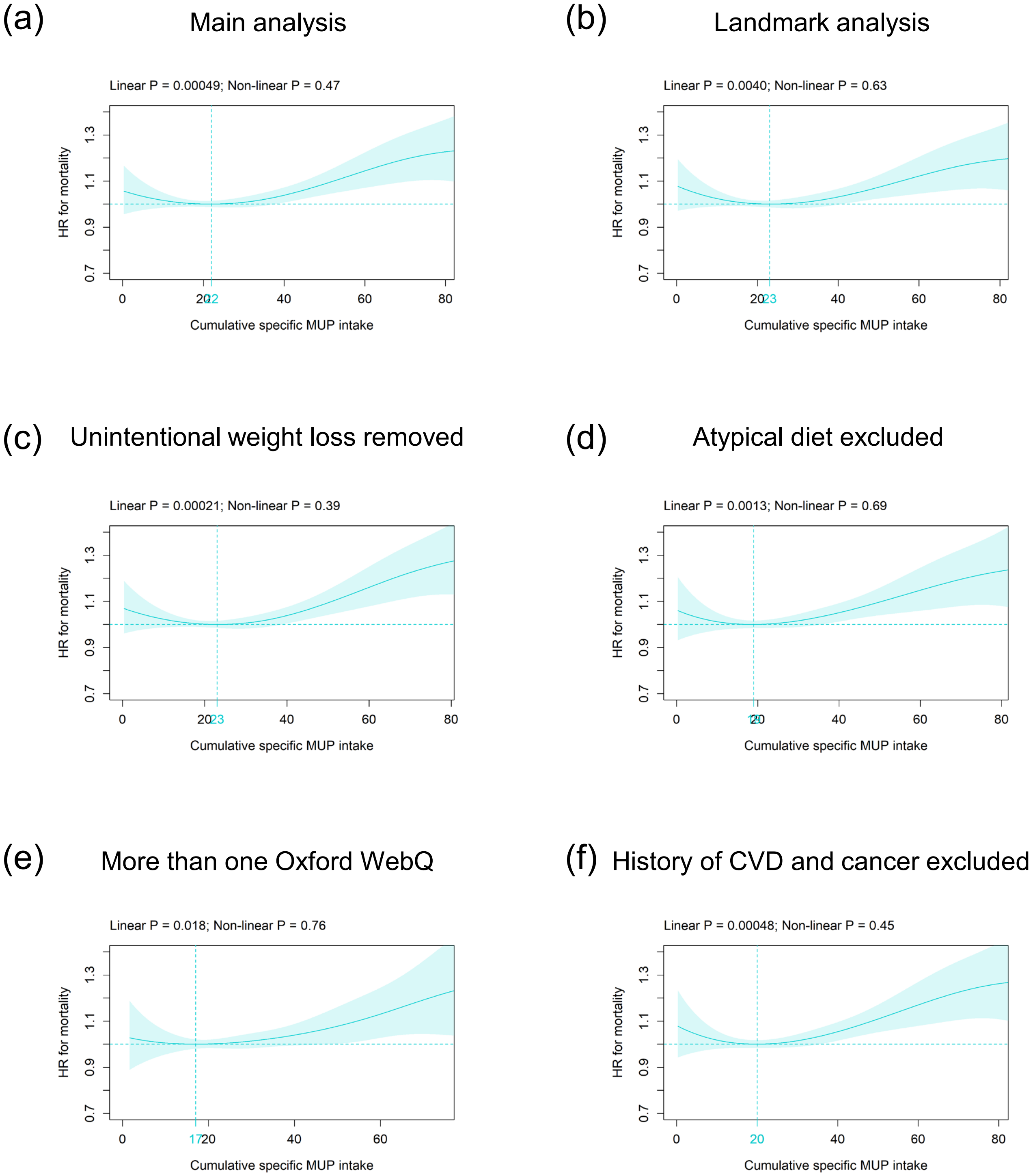

Fig. S20 - Cumulative specific MUP intake

Tab. S1. Specific MUPs and their individual compounds within the nine categories.[a]

Categories	Specific MUPs	Individual compounds
**Cosmetic additives**		
**1. Flavour**	**1. Flavour***	Flavor<br>Flavour
**2. Flavour enhancer**	**2. Glutamate**	E620 – E625<br>Glutamate<br>Glutamic acids<br>MSG
	3. Glycine	E640<br>Glycinate<br>Glycine
	4. Guanylate	E626 – E629<br>Guanylate<br>Guanylic acids
	5. Inosinate	E630 – E633<br>Inosinate<br>Inosinic acids
	6. Maltol	E636 – E637<br>Maltol
	**7. Ribonucleotide**	5'-ribonucleotides<br>E634 – E635
	8. Zinc acetate	E650<br>Zinc (di)acetate
**3. Colouring agent**	**9. Colour***	Color<br>Color stabilizer<br>Colour<br>Colour stabiliser
	10. Dye	Dye
**4. Sweetener**	**11. Acesulfame**	Acesulfame K<br>E950
	12. Advantame	Advantame<br>E969
	13. Alitame	Alitame<br>E956
	14. Aspartame	Aspartame<br>E951
	15. Aspartame-Acesulfame	Aspartame-Acesulfame<br>E962
	16. Cyclamate	Cyclamate<br>Cyclamic acid<br>E952
	17. Erythritol	E968<br>Erythritol
	18. Glycyrrhizin	E958<br>Glycyrrhizic acid<br>Glycyrrhizin
	19. Isomalt	E953<br>Isomalt
	20. Lactitol	E966<br>Lactitol
	21. Maltitol	E965<br>Maltitol
	22. Mannitol	E421<br>Mannitol

Tab. S1. Specific MUPs and their individual compounds within the nine categories.[a] (continued)

Categories	Specific MUPs	Individual compounds
**Cosmetic additives**		
**4. Sweetener**	*23. Neohesperidine*	E959<br>Neohesperidine DC
	24. Neotame	E961<br>Neotame
	25. Polyglycitol	E964<br>Polyglycitol
	**26. Saccharin**	E954<br>Saccharin
	27. Sorbitol	E420<br>Sorbitol
	28. Steviol	E960<br>Steviol glycoside
	**29. Sucralose**	E955<br>Sucralose
	30. Tagatose	E963<br>Tagatose
	31. Thaumatin	E957<br>Thaumatin
	32. Xylitol	E967<br>Xylitol
5. Processing aid	33. Bulking agent	Anti-bulking<br>Bulking agent
	**34. Caking agent**	Anti-caking agent<br>Anticaking agent
	35. Carbonating agent	Carbonating agent
	36. Emulsifier	Emulsifier<br>Emulsifying salts
	**37. Firming agent**	Firming agent
	38. Foaming agent	Anti-foaming agent<br>De-foaming agent<br>Foaming agent
	**39. Gelling agent**	Gelling agent
	40. Glazing agent	Glazing agent
	41. Humectant	Humectant
	42. Sequestrant	Sequestrant
	**43. Thickener**	Thickener
**Non-culinary ingredients**		
**6. Varieties of sugar**	44. Dextrose	Dextrose<br>Polydextrose#
	**45. Fructose**	Fructose<br>Fructose-glucose syrup#<br>Glucose-fructose syrup#<br>High-fructose corn syrup
	**46. Inverted sugar**	Inverted refiners syrup#<br>Inverted sugar syrup#<br>Invert sugar
	**47. Lactose**	Lactose
	**48. Maltodextrin**	Maltodextrin
7. Modified oil	49. Hydrogenated oil*	Hydrogenated oil
	50. Interesterified oil	Interesterified oil
8. Protein source	*51. Casein*	Casein
	52. Gluten	Gluten

Tab. S1. Specific MUPs and their individual compounds within the nine categories.[a] (continued)

Categories	Specific MUPs	Individual compounds
**Non-culinary ingredients**		
8. Protein source	53. Hydrolysed protein	Hydrolysed protein<br>Hydrolyzed protein
	54. Isolated protein	Isolated soy protein<br>Protein isolate[#]<br>Soy protein isolate
	55. Mechanically separated meat	Mechanically separated meat
	56. Whey	Dried whey[#]<br>Whey<br>Whey derivatives[#]<br>Whey permeate[#]<br>Whey powder[#]<br>Whey protein<br>Whey solids[#]
9. Fibre	57. Fibre*	Fiber<br>Fibre<br>Fibre isolate[#]<br>Insoluble fibre<br>Soluble fibre

[a]Individual compounds are based on NOVA group 4.[1, 2] [#]Indicates individual compounds which are not literally mentioned in the two publications[1, 2] but which are related to the ultra-processing compounds mentioned. A total of 37 out of 57 MUPs was detected in at least one commercial product. **Bold** indicates a significant (linear and/or non-linear P<0·05) association with all-cause mortality in the main analysis. *Italics* indicates that the MUP was not present in any commercial product. MSG, Monosodium glutamate; MUP, Marker of ultra-processing. *These MUPs are identical to the respective MUP category since either the search term is identical (i.e., flavour and fibre) or only one search term is present in at least one ingredient list (i.e., colour and hydrogenated oil) and, therefore, results are only shown for the MUP category.

References

1. Monteiro CA, Cannon G, Moubarac J-C, Levy RB, Louzada MLC, Jaime PC. The UN Decade of Nutrition, the NOVA food classification and the trouble with ultra-processing. *Public Health Nutr* 2017; **21**(1):5–17.

2. Monteiro CA, Cannon G, Levy RB et al. Ultra-processed foods: what they are and how to identify them. *Public Health Nutr* 2019; **22**(5):936–41.

Tab. S2. Example of MLI calculation for flavour for chocolate bars; UK Biobank Data Field 102260; Oxford WebQ question: "Did you eat any chocolate bars (e.g. Crunchie, Snickers) yesterday?"[a]

**Product name**	**Ingredient list**	**Flavour**
Cadbury Dairy Milk Chocolate Bar	Milk**, Sugar, Cocoa Butter, Cocoa Mass, Vegetable Fats (Palm, Shea), Emulsifiers (E442, E476), **Flavour**ings	1
Cadbury Twirl Chocolate Bars	Milk, Sugar, Cocoa Butter, Cocoa Mass, Vegetable Fats (Palm, Shea), Emulsifier (E442), **Flavour**ings	1
Mars	Sugar, Glucose Syrup, Skimmed Milk Powder, Cocoa Butter, Cocoa Mass, Sunflower Oil, Milk Fat, Palm Fat, Lactose, Whey Permeate (from Milk), Fat Reduced Cocoa, Barley Malt Extract, Emulsifier (Soya Lecithin), Salt, Egg White Powder, Milk Protein, Vanilla Extract, Milk Chocolate contains Milk Solids 14% minimum, Milk Chocolate contains Vegetable Fats in addition to Cocoa Butter	0
Kit Kat Chunky Milk Chocolate Bar	Sugar, Wheat Flour (contains Calcium, Iron, Thiamin and Niacin), Milk Powders (Whole and Skimmed), Cocoa Mass, Cocoa Butter, Vegetable Fats (Palm, Palm Kernel, Shea), Lactose and Proteins from Whey (from Milk), Whey Powder (from Milk), Emulsifier (Sunflower Lecithin), Yeast, Raising Agent (Sodium Bicarbonate), Butterfat (from Milk), Natural **Flavour**ings	1
Galaxy Ripple 4 Pack	Sugar, Cocoa Butter, Skimmed Milk Powder, Cocoa Mass, Lactose and Protein from Whey (from Milk), Vegetable Fat (Palm Fat), Whey Powder (from Milk), Milk Fat, Emulsifiers (Soya Lecithin, E476), Water, Natural **Flavour** (Vanilla Extract), Milk Chocolate contains Milk Solids 14% minimum and Cocoa Solids 25% minimum, Milk Chocolate contains Vegetable Fats in addition to Cocoa Butter	1
Cadbury Dairy Milk Freddo Bars	Milk**, Sugar, Cocoa Butter, Cocoa Mass, Vegetable Fats (Palm, Shea), Emulsifiers (E442, E476), **Flavour**ings	1
Cadbury Crunchy Bars	Sugar, Glucose Syrup, Cocoa Butter, Cocoa Mass, Skimmed Milk Powder, Whey Permeate Powder (from Milk), Palm Oil, Milk Fat, Emulsifier (E442), **Flavour**ings, Coconut Oil, Milk Chocolate: Milk Solids 14 % minimum, Contains Vegetable Fats in addition to Cocoa Butter	1
Tesco Crunchy Caramel Bars	Milk Chocolate (36%) [Sugar, Cocoa Butter**, Dried Whole Milk, Cocoa Mass**, Milk Sugar, Whey Powder (Milk), Emulsifier (Soya Lecithins), Salt, Vanilla Extract], Wheat Flour, Palm Fat, Sugar, Glucose Syrup, Condensed Skimmed Milk, Humectant (Sorbitol Syrup), Caramelised Sugar Syrup, Salt, Barley Malt Extract, Fat Reduced Cocoa Powder**, Raising Agent (Sodium Carbonates), Emulsifier (Soya Lecithins)	0
Snickers 4 Pack	Sugar, Peanuts, Glucose Syrup, Skimmed Milk Powder, Cocoa Butter, Cocoa Mass, Sunflower Oil, Palm Fat, Lactose and Protein from Whey (from Milk), Whey Powder (from Milk), Milk Fat, Emulsifier (Soya Lecithin), Salt, Coconut Oil, Egg White Powder, Natural Vanilla Extract, Milk Protein, Milk Chocolate contains Milk Solids 14% minimum, Milk Chocolate contains Vegetable Fats in addition to Cocoa Butter	0
Snickers Creamy Peanut Butter Chocolate Trio Bar	Peanuts, Sugar, Glucose Syrup, Skimmed Milk Powder, Cocoa Butter, Lactose, Invert Sugar, Cocoa Mass, Dextrose, Sunflower Oil, Whey Powder (from Milk), Milk Fat, Humectants (Glycerol, Sorbitol), Hydrogenated Soya Fat, Palm Fat, Salt, Emulsifier (Soya Lecithin), Antioxidant (E306), Starch, Natural Vanilla Extract, Milk Chocolate contains Milk Solids 14% minimum	0
	**MLI for flavour:**	**0.6**

[a]The search term flavour is indicated in **bold**. MLI, Marker Likelihood Index.

Tab. S3. Intake of specific MUPs at baseline in both the total cohort and the subgroups based on %TFI-UPF intake quintiles.[a]

Parameters in %TFI	Total cohort (n = 186,744)	Quintiles of UPF intake (%TFI)				
		0·4 to 12·9 (n = 37,349)	12·9 to 16·5 (n = 37,349)	16·5 to 20·3 (n = 37,348)	20·3 to 26·0 (n = 37,349)	26·0 to 98·7 (n = 37,349)
	Mean (SD)	Mean (SD)	Mean (SD)	Mean (SD)	Mean (SD)	Mean (SD)
Glutamate	0·3 (0·5)	0·2 (0·4)	0·3 (0·4)	0·3 (0·5)	0·4 (0·6)	0·4 (0·7)
Guanylate	0·0 (0·1)	0·0 (0·0)	0·0 (0·1)	0·0 (0·1)	0·0 (0·1)	0·0 (0·1)
Inosinate	0·0 (0·1)	0·0 (0·0)	0·0 (0·1)	0·0 (0·1)	0·0 (0·1)	0·0 (0·1)
Ribonucleotide	0·3 (0·5)	0·2 (0·4)	0·3 (0·4)	0·3 (0·5)	0·4 (0·5)	0·4 (0·7)
Acesulfame	2·3 (4·3)	0·3 (0·6)	0·8 (1·2)	1·4 (1·9)	2·5 (2·9)	6·6 (7·2)
Aspartame	1·3 (2·0)	0·3 (0·5)	0·6 (0·7)	0·9 (1·0)	1·4 (1·5)	3·2 (3·4)
Erythritol	0·0 (0·1)	0·0 (0·1)	0·0 (0·1)	0·0 (0·1)	0·0 (0·1)	0·0 (0·1)
Isomalt	0·1 (0·2)	0·0 (0·1)	0·1 (0·2)	0·1 (0·2)	0·1 (0·2)	0·1 (0·3)
Maltitol	0·1 (0·1)	0·0 (0·1)	0·0 (0·1)	0·1 (0·1)	0·1 (0·1)	0·1 (0·1)
Saccharin	0·3 (0·7)	0·1 (0·2)	0·1 (0·3)	0·2 (0·5)	0·3 (0·6)	0·6 (1·3)
Sorbitol	0·1 (0·1)	0·1 (0·1)	0·1 (0·1)	0·1 (0·1)	0·1 (0·2)	0·1 (0·2)
Steviol	0·6 (1·8)	0·2 (0·3)	0·3 (0·5)	0·4 (0·7)	0·6 (1·1)	1·6 (3·5)
Sucralose	2·3 (4·0)	0·2 (0·7)	0·7 (1·2)	1·4 (1·9)	2·6 (2·8)	6·8 (6·3)
Xylitol	0·0 (0·0)	0·0 (0·0)	0·0 (0·0)	0·0 (0·0)	0·0 (0·0)	0·0 (0·0)
Bulking agent	0·1 (0·1)	0·1 (0·1)	0·1 (0·1)	0·1 (0·1)	0·1 (0·1)	0·1 (0·2)
Caking agent	0·5 (1·2)	0·2 (0·4)	0·3 (0·7)	0·5 (0·9)	0·7 (1·3)	0·9 (2·0)
Emulsifier	5·3 (3·0)	3·1 (1·5)	4·4 (1·9)	5·3 (2·2)	6·2 (2·7)	7·6 (3·8)
Firming agent	1·1 (1·0)	0·9 (0·8)	1·1 (0·9)	1·1 (1·0)	1·1 (1·0)	1·1 (1·1)
Foaming agent	0·0 (0·0)	0·0 (0·0)	0·0 (0·0)	0·0 (0·0)	0·0 (0·0)	0·0 (0·0)
Gelling agent	0·6 (0·5)	0·4 (0·3)	0·6 (0·4)	0·6 (0·5)	0·7 (0·5)	0·8 (0·6)
Glazing agent	0·2 (0·4)	0·1 (0·3)	0·2 (0·3)	0·2 (0·4)	0·2 (0·4)	0·2 (0·5)
Humectant	0·5 (0·7)	0·3 (0·5)	0·5 (0·6)	0·5 (0·7)	0·6 (0·8)	0·7 (0·9)
Sequestrant	0·0 (0·0)	0·0 (0·0)	0·0 (0·0)	0·0 (0·0)	0·0 (0·0)	0·0 (0·1)
Thickener	0·9 (1·2)	0·4 (0·4)	0·6 (0·7)	0·9 (0·9)	1·2 (1·2)	1·7 (1·8)
Dextrose	2·6 (1·8)	1·5 (0·9)	2·1 (1·1)	2·2 (1·3)	3·0 (1·7)	4·0 (2·4)
Fructose	0·8 (0·7)	0·4 (0·3)	0·6 (0·4)	0·7 (0·5)	0·9 (0·6)	1·3 (1·0)
Inverted sugar	0·5 (0·6)	0·3 (0·4)	0·5 (0·5)	0·6 (0·5)	0·6 (0·6)	0·7 (0·7)
Lactose	0·5 (0·7)	0·2 (0·3)	0·3 (0·4)	0·4 (0·5)	0·5 (0·6)	0·7 (1·0)
Maltodextrin	0·9 (0·8)	0·7 (0·5)	0·8 (0·6)	0·9 (0·7)	1·0 (0·8)	1·2 (1·1)
Gluten	1·3 (1·2)	0·9 (0·7)	1·2 (0·9)	1·3 (1·0)	1·5 (1·2)	1·7 (1·6)
Hydrolysed protein	0·1 (0·1)	0·1 (0·1)	0·1 (0·1)	0·1 (0·1)	0·1 (0·1)	0·1 (0·2)
Isolated protein	0·0 (0·1)	0·0 (0·0)	0·0 (0·1)	0·0 (0·1)	0·0 (0·1)	0·0 (0·1)
Whey protein	1·4 (1·3)	0·7 (0·7)	1·1 (0·9)	1·4 (1·1)	1·6 (1·4)	2·0 (1·9)

[a]%TFI, Percentage total food intake; MUP, Marker of ultra-processing; SD, Standard deviation; UPF, Ultra-processed food.

Tab. S4. Baseline characteristics of included as compared to excluded participants.[a]

Parameters	Included participants (n = 186,744)	Excluded participants (n = 24,095)
Age (years)	58 (8)	59 (8)
Alcohol (g/day)		
- <1	66,643 (35·7)	12,516 (51·9)
- 1 to <8	19,958 (10·7)	2,028 (8·4)
- 8 to <16	26,317 (14·1)	2,363 (9·8)
- ≥16	73,826 (39·5)	7,188 (29·8)
BMI (kg/m²)		
- <18·5	1,071 (0·6)	73 (0·3)
- 18·5 to <25	72,940 (39·1)	4,364 (18·6)
- 25 to <30	78,358 (42·0)	8,927 (38·0)
- ≥30	34,375 (18·4)	10,137 (43·1)
Energy (kJ)	8,915 (2,248)	7,855 (3,774)
Ethnic background		
- White	180,017 (96·4)	21,114 (90·5)
- Group composed of Mixed, Asian, Black, Chinese, and other	6,727 (3·6)	2,210 (9·5)
General health status		
- Poor	4,536 (2·4)	2,126 (9·0)
- Fair	29,915 (16·0)	7,316 (31·1)
- Good	113,277 (60·7)	11,856 (50·4)
- Excellent	39,016 (20·9)	2,231 (9·5)
Highest qualification		
- None of the below	14,993 (8·0)	3,083 (13·4)
- National exams at age 16 years	28,129 (15·1)	3,657 (15·9)
- Vocational qualifications or optional national exams at ages 17 to 18 years	33,080 (17·7)	4,604 (20·0)
- Professional	29,031 (15·6)	3,556 (15·4)
- College or university	81,511 (43·7)	8,152 (35·4)
History of psychiatric disease		
- Yes	12,290 (6·6)	1,916 (8·0)
- No	174,454 (93·4)	22,179 (92·1)
Household income/year (k£)		
- <18	25,012 (13·4)	4,509 (19·0)
- 18 to <31	40,844 (21·9)	5,122 (21·6)
- 31 to <52	48,463 (26·0)	5,321 (22·4)
- 52 to <100	41,711 (22·3)	4,271 (18·0)
- ≥100	12,407 (6·6)	1,194 (5·0)
- Unknown	18,307 (9·8)	3,314 (14·0)
Physical activity (MET per week)	4,133 (2,653)	3,836 (2,961)
SBP (mmHg)	138·5 (19·4)	141·1 (18·8)
Sex		
- Female	106,958 (57·3)	9,185 (38·1)
- Male	79,786 (42·7)	14,910 (61·9)
Smoking status		
- Never	107,346 (57·5)	11,590 (51·6)
- Previous	65,959 (35·3)	8,843 (39·4)
- Current occasional	4,474 (2·4)	644 (2·9)
- <10 cigarettes per day	2,306 (1·2)	263 (1·2)
- 10 to 14 cigarettes per day	2,010 (1·1)	323 (1·4)
- 15 to 19 cigarettes per day	1,793 (1·0)	272 (1·2)
- ≥20 cigarettes per day	2,856 (1·5)	527 (2·3)
Townsend deprivation index	-1·7 (2·8)	-0·9 (3·2)

Tab. S4. Baseline characteristics of included as compared to excluded participants.[a] (continued)

UPF intake (%TFI)	20·0 (8·9)	20·7 (11·2)
MUP category intake (%TFI)		
- Flavour	13·6 (8·3)	14·7 (10·4)
- Flavour enhancer	0·3 (0·5)	0·3 (0·6)
- Colouring agent	5·6 (4·1)	6·2 (5·2)
- Sweetener	4·6 (6·0)	6·1 (8·5)
- Processing aid	7·6 (3·5)	7·1 (3·9)
- Varieties of sugar	4·7 (2·5)	4·4 (2·8)
- Modified oil	0·0 (0·1)	0·0 (0·1)
- Protein source	2·7 (1·9)	2·4 (2·1)
- Fibre	1·0 (0·9)	1·0 (1·1)

[a]Categorical variables are summarised as frequencies (percentages) and continuous variables as mean (SD). A total of 10,203 and 2,543 deaths occurred in included and excluded participants, respectively. Number of NA's are BMI: 594, ethnic background: 771, general health status: 566, highest qualification: 1,043, household income: 364, smoking status: 1,633, SBP: 177, Townsend deprivation index: 265, UPF and MUP category intake: 4. %TFI, Percentage total food intake; BMI, Body mass index; MET, Metabolic equivalent of task; MUP, Marker of ultra-processing; SBP, Systolic blood pressure; SD Standard deviation; UPF, Ultra-processed food.

Tab. S5. Association of UPF, MUP categories, and specific MUPs with all-cause mortality in all sensitivity analyses. Results are only shown for associations which were significant in the main analyses.[a]

		(1)	(2)	(3)	(4)	(5)	(6)	(7)	(8)	(9)	(10)	(11)	(12)	(13)	(14)
	Fig.	S5	S6	S7	S8	S9	S10	S11	S12	S13	S14	S15	S16	S17	S18
UPF	a	s	s	s	s	s	s	s	s	s	s	s	s	s	s
Category															
- Flavour	b	s	s	s	s	s	s	s	s	s	s	s	s	s	s
- Flavour enhancer	c	s	ns	s	ns	s	s	s	s	s	ns	ns	s	s	s
- Colouring agent	d	s	s	s	s	s	s	s	s	s	s	s	s	s	s
- Sweetener	e	s	s	s	s	s	s	s	s	s	s	s	s	s	s
- Varieties of sugar	g	ns	s	s	ns	s	ns	s	s	s	ns	ns	s	s	s
MUP															
- Glutamate	k	ns	s	s	ns	s	s	s	s	s	ns	ns	s	s	s
- Ribonucleotide	l	s	s	s	ns	s	s	s	s	s	s	s	s	s	s
- Acesulfame	m	ns	s	s	s	ns	ns	s	s	s	s	s	s	s	s
- Saccharin	n	s	s	s	s	s	s	s	s	s	s	s	s	s	s
- Sucralose	o	s	s	s	s	s	s	s	s	s	s	s	s	s	s
- Caking agent	p	s	s	s	ns	s	ns	s	s	s	s	s	s	s	s
- Firming agent	q	ns	ns	s	ns	ns	ns	s	s	s	ns	ns	s	s	s
- Gelling agent	r	s	s	ns	ns	ns	s	s	s	ns	ns	ns	ns	s	s
- Thickener	s	s	s	s	s	s	ns	s	s	s	s	s	s	s	s
- Fructose	t	s	s	s	s	s	s	s	s	s	s	s	s	s	s
- Inverted sugar	u	s	s	s	ns	s	ns	s	s	s	s	s	s	s	s
- Lactose	v	s	s	s	ns	s	s	s	s	s	s	s	s	s	s
- Maltodextrin	w	ns	s	s	ns	ns	ns	ns	s	ns	ns	ns	ns	s	s

[a]The respective supplemental figures are indicated. The following sensitivity analyses are summarised: (1) Landmark analysis, (2) Unintentional weight loss removed, (3) Atypical diet excluded, (4) More than one Oxford WebQ, (5) History of CVD and cancer excluded, (6) First Oxford WebQ only, (7) Further adjusted for diet quality, (8) WHR and height instead of BMI, (9) Energy intake removed, (10) Imputation k nearest neighbour, (11) Imputation random forrest, (12) Further adjusted for cardiometabolic disease medication, (13) Include BMI and SBP as spline terms, (14) Further adjusted for assessment centre. BMI, Body mass index; CVD, Cardiovascular disease; MUP, Marker of ultra-processing; ns, Non-significant; s, Significant, SBP, Systolic blood pressure; UPF, Ultra-processed food; WHR, Waist-to-hip ratio.

Tab. S6. Association of MUP categories and specific MUPs with all-cause mortality in all sensitivity analyses. Results are only shown for associations which were not significant in the main analyses.[a]

	(1)	(2)	(3)	(4)	(5)	(6)	(7)	(8)	(9)	(10)	(11)	(12)	(13)	(14)
Category														
- Processing aid	ns	ns	ns	ns	ns	ns	ns	ns	ns	ns	ns	ns	ns	ns
- Modified oil	ns	ns	ns	ns	ns	ns	ns	ns	ns	ns	ns	ns	ns	ns
- Protein source	ns	ns	ns	ns	ns	ns	ns	ns	ns	ns	ns	ns	s	ns
- Fibre	ns	ns	ns	ns	ns	ns	ns	ns	ns	ns	ns	ns	ns	ns
MUP														
- Guanylate	ns	ns	ns	ns	ns	ns	ns	ns	ns	ns	ns	ns	ns	ns
- Inosinate	ns	ns	ns	ns	ns	ns	ns	ns	ns	ns	ns	ns	ns	ns
- Aspartame	ns	s	ns	ns	ns	ns	ns	ns	ns	s	s	ns	s	ns
- Erythritol	s	ns	ns	ns	ns	s	ns	ns	ns	s	s	ns	s	ns
- Isomalt	ns	ns	ns	ns	ns	ns	ns	ns	ns	ns	ns	ns	ns	ns
- Maltitol	ns	ns	ns	ns	ns	ns	ns	ns	ns	s	s	ns	ns	ns
- Sorbitol	ns	ns	ns	ns	ns	ns	ns	ns	ns	ns	ns	ns	ns	ns
- Steviol	ns	ns	ns	ns	ns	ns	ns	ns	ns	ns	ns	ns	ns	ns
- Xylitol	s	ns	ns	ns	ns	s	ns	ns	ns	s	s	ns	s	ns
- Bulking agent	s	ns	ns	ns	ns	ns	ns	ns	ns	s	s	ns	s	ns
- Emulsifier	ns	ns	ns	ns	ns	ns	ns	ns	ns	ns	ns	ns	ns	ns
- Foaming agent	ns	ns	ns	ns	ns	ns	ns	ns	ns	ns	ns	ns	ns	ns
- Glazing angent	ns	ns	ns	ns	ns	ns	ns	ns	ns	ns	ns	ns	ns	ns
- Humectant	ns	ns	ns	ns	ns	ns	ns	ns	ns	ns	ns	ns	ns	ns
- Sequestrant	ns	ns	ns	ns	ns	s	ns	ns	ns	ns	ns	ns	ns	ns
- Dextrose	ns	ns	ns	ns	ns	ns	ns	ns	ns	ns	ns	ns	ns	ns
- Gluten	ns	ns	s	ns	ns	ns	ns	ns	ns	ns	ns	ns	s	ns
- Hydrolysed protein	ns	ns	ns	ns	ns	ns	ns	ns	ns	ns	ns	ns	ns	ns
- Isolated protein	s	ns	ns	ns	ns	ns	ns	ns	ns	ns	ns	ns	s	ns
- Whey protein	ns	ns	s	ns	ns	ns	ns	ns	ns	ns	ns	ns	s	ns

[a]The following sensitivity analyses are summarised: (1) Landmark analysis, (2) Unintentional weight loss removed, (3) Atypical diet excluded, (4) More than one Oxford WebQ, (5) History of CVD and cancer excluded, (6) First Oxford WebQ only, (7) Further adjusted for diet quality, (8) WHR and height instead of BMI, (9) Energy intake removed, (10) Imputation k nearest neighbour, (11) Imputation random forrest, (12) Further adjusted for cardiometabolic disease medication, (13) Include BMI and SBP as spline terms, (14) Further adjusted for assessment centre. BMI, Body mass index; CVD, Cardiovascular disease; MUP, Marker of ultra-processing; ns, Non-significant; s, Significant, SBP, Systolic blood pressure; UPF, Ultra-processed food; WHR, Waist-to-hip ratio.

Tab. S7. Association of MUP categories and specific MUPs with all-cause mortality in the main and all sensitivity analyses further adjusted for multiple testing using the Benjamini-Hochberg method for the MUP categories and specific MUPs separately. Results with a false discovery rate adjusted p-value <0·05 were considered as statistically significant and are only shown for associations which were significant in the main analyses without adjustment for multiple testing.[a]

	Main	(1)	(2)	(3)	(4)	(5)	(6)	(7)	(8)	(9)	(10)	(11)	(12)	(13)	(14)
Category															
- Flavour	s	s	s	s	s	s	s	s	s	s	s	s	s	s	s
- Flavour enhancer	s	ns	ns	s	ns	ns	ns	ns	ns	ns	ns	ns	ns	s	ns
- Colouring agent	s	s	s	s	s	s	s	s	s	s	s	s	s	s	s
- Sweetener	s	s	s	s	s	s	s	ns	s	s	s	s	s	s	s
- Varieties of sugar	s	s	ns	s	ns	s	ns	ns	ns	s	ns	ns	s	s	ns
MUP															
- Glutamate	ns	ns	ns	ns	ns	ns	ns	ns	ns	ns	ns	ns	ns	s	ns
- Ribonucleotide	ns	ns	ns	s	ns	ns	ns	ns	ns	ns	ns	ns	ns	s	ns
- Acesulfame	ns	ns	ns	ns	ns	ns	ns	ns	ns	ns	s	s	ns	ns	ns
- Saccharin	ns	s	s	s	ns	ns	ns	ns	ns	ns	ns	ns	ns	s	ns
- Sucralose	s	ns	s	ns	ns	ns	ns	ns	s	s	s	s	s	s	s
- Caking agent	s	ns	s	s	ns	ns	ns	s	s	s	ns	s	s	s	s
- Firming agent	ns	ns	ns	ns	ns	ns	ns	ns	ns	ns	ns	ns	ns	s	ns
- Gelling agent	ns	ns	ns	ns	ns	ns	ns	ns	ns	ns	ns	ns	ns	ns	ns
- Thickener	ns	ns	s	s	ns	ns	ns	ns	ns	s	ns	s	s	s	ns
- Fructose	s	ns	s	s	s	s	ns	s	s	s	s	s	s	s	s
- Inverted sugar	ns	ns	ns	ns	ns	ns	ns	ns	ns	ns	ns	ns	ns	ns	ns
- Lactose	s	s	s	s	ns	ns	ns	s	s	s	s	s	s	s	s
- Maltodextrin	ns	ns	ns	s	ns	ns	ns	ns	ns	ns	ns	ns	ns	s	ns

[a]The following sensitivity analyses are summarised: (1) Landmark analysis, (2) Unintentional weight loss removed, (3) Atypical diet excluded, (4) More than one Oxford WebQ, (5) History of CVD and cancer excluded, (6) First Oxford WebQ only, (7) Further adjusted for diet quality, (8) WHR and height instead of BMI, (9) Energy intake removed, (10) Imputation k nearest neighbour, (11) Imputation random forrest, (12) Further adjusted for cardiometabolic disease medication, (13) Include BMI and SBP as spline terms, (14) Further adjusted for assessment centre. BMI, Body mass index; CVD, Cardiovascular disease; MUP, Marker of ultra-processing; ns, Non-significant; s, Significant, SBP, Systolic blood pressure; UPF, Ultra-processed food; WHR, Waist-to-hip ratio.
